# Pharmacological Potential of Lathyrane-Type Diterpenoids from Phytochemical Sources

**DOI:** 10.3390/ph15070780

**Published:** 2022-06-23

**Authors:** Fátima Vela, Abdellah Ezzanad, Alan Christy Hunter, Antonio José Macías-Sánchez, Rosario Hernández-Galán

**Affiliations:** 1 Departamento de Química Orgánica and Instituto de Investigación en Biomoléculas [INBIO], Facultad de Ciencias, Universidad de Cádiz, Puerto Real, 11510 Cadiz, Spain; fatima.vela@uca.es (F.V.); abdellah.ezzanad@alum.uca.es (A.E.); antoniojose.macias@uca.es (A.J.M.-S.); 2 School of Pharmacy, College of Science, University of Lincoln, Lincolns LN6 7DL, UK; chunter@lincoln.ac.uk

**Keywords:** lathyrane, biological activity, diterpene, *Euphorbia*

## Abstract

Lathyrane diterpenoids are one of the primary types of secondary metabolites present in the genus *Euphorbia* and one of the largest groups of diterpenes. They are characterized by having a highly oxygenated tricyclic system of 5, 11 and 3 members. These natural products and some synthetic derivatives have shown numerous interesting biological activities with clinical potential against various diseases, such as cytotoxic activity against cancer cell lines, multi-drug resistance reversal, antiviral properties, anti-inflammatory activity and their capability to induce proliferation or differentiation into neurons of neural progenitor cells. The structure of the lathyrane skeleton could be considered privileged because its framework is able to direct functional groups in a well-defined space. The favorable arrangement of these makes interaction possible with more than one target. This review aims to highlight the evidence of lathyranes as privileged structures in medicinal chemistry. Chemical structures of bioactive compounds, the evaluation of biological properties of natural and semisynthetic derivatives, and the exploration of the mechanisms of action as well as target identification and some aspects of their targeted delivery are discussed.

## 1. Introduction

**Figure 1:**
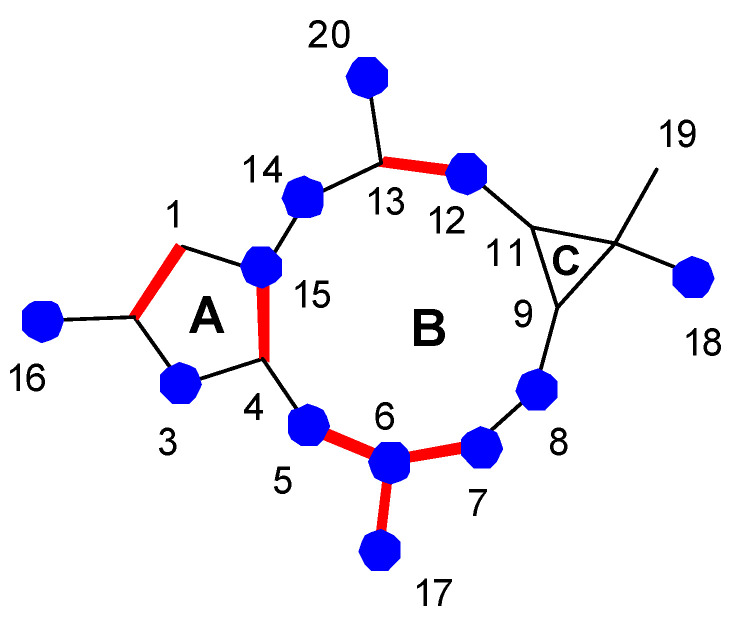
General layout of bioactive lathyrane derivatives, with no stereochemical detail. Carbocyclic rings are labeled with the letters A, B and C. Solid blue dots indicate the positions where an oxygenated functional group has been described (alcohol, ether/epoxide, ester, ketone). Broadened red lines show positions where double bonds have been described.

Lathyrane diterpenoids are one of the main chemical components present in the genus *Euphorbia* and one of the largest groups of diterpenes. They are characterized by a twenty carbon skeleton that has a highly oxygenated tricyclic system of 5, 11 and 3 members [1]. As shown in Figure 1, its structural diversity mainly arises from the modifications (redox, etherification or esterification) of the 3, 5 and 11-membered rings [2]. These compounds are usually substituted with various acyl groups; some of the most frequently found ones, from natural sources, are acetyl, benzoyl and phenylacetyl groups. Methoxyl, tiglyl or cinnamoyl groups are also quite widespread in lathyranes from natural sources. Specific functionalization patterns can be found in the Supplementary Material section (Sections S1–S11, including Tables S1–S11), where bioactive lathyranes described in the literature (up to March 2022) have been arranged according to specific structural classes. Discussions on the structure–activity relationships of these compounds are the main topic of this review.

Therapeutic applications of herbal remedies containing lathyranes trace back over thousands of years, especially in traditional Chinese medicine. They find use for the treatment of different medical disorders, such as skin diseases, migraine, edema, intestinal parasites and gonorrhea [1,3]. These natural products and some synthetic derivatives have shown numerous interesting biological activities with clinical potential such as cytotoxicity, multidrug resistance reversal (MDR) ability, antiviral properties, anti-inflammatory activity and capability to induce neural progenitor cell (NPC) proliferation or differentiation into neurons [3,4,5].

The structure of lathyrane could be considered privileged because its framework is able to direct functional groups in a well-defined space [6]. The favorable arrangement of the functional groups that decorate the lathyrane skeleton makes interaction with more than one target possible. For example, the acylation pattern is a critical factor in the reversal of MDR, where aromatic moieties are of fundamental importance [7,8]. Nevertheless, other factors such as lipophilicity or the presence of a free hydroxyl group at C-3 appear to be of significance [9,10]. Furthermore, some additional structural features, such as the fused epoxy ring, also appear to play an essential role. On the other hand, the *gem*-dimethylcyclopropane subunit contained within the lathyrane framework is important for substrate–target biological interactions, as is frequently found in bioactive diterpenes [2]; nevertheless, no clear evidence exists in full discerning its function. Therefore, there is not a single factor that determines the activity of these compounds, which depends on the balance between a set of factors.

Several reviews have been published covering diterpenes from *Euphorbia* species [11,12,13]. In 2014, Vasas and Hohmann’s review article covered all diterpenoids isolated from *Euphorbia* between 2008 and 2012, including 48 lathyrane diterpenes and their biological activities [3]. In the same year, Ferreira et al. posted a review covering metabolites from *Euphorbia* and *Momordica* that could overcome multidrug resistance [14]. The optimal activity was obtained with macrocyclic diterpenes containing jatrophane and lathyrane scaffolds. Also in 2014, Durán-Peña et al. published a review covering occurrence and biological activity of diterpenes containing a *gem*-dimethylcyclopropane subunit of specific bioactive diterpenes [2]. They demonstrated that antiviral activity, cytotoxicity against cancer cell lines and modulation of multidrug resistance (MDR) were the principal activities showed by lathyrane diterpenoids. No reviews to date have covered the phytochemical potential of lathyranes. In addition, none of these reviews was published recently. This paper aims to highlight the evidence of lathyranes as privileged structures in medicinal chemistry. Multiple aspects of the lathyranes are summarized, including the isolation of new biologically active derivatives, the evaluation of biological properties of natural and semisynthetic derivatives, and the exploration of the underlying mechanisms of action as well as target identification. Some aspects of their targeted delivery for the treatment of specific diseases are also discussed. To compose the manuscript, the authors performed a systematic search in Scifinder, Scopus, PubMed, Google Scholar and Web of Science databases. In addition, the keywords that were combined and used in the search were: lathyranes, lathyrol, jolkinol, laurifolioside, jatrogrossidion, ingol, biological activity and *Euphorbia*.

## 2. Bioactive Lathyranes

Lathyrane diterpenes have primarily been isolated from *Euphorbia* species and they accumulate in all parts of the plant. Procedures describe their extraction at room temperature by maceration and isolation through multistep separation protocols [13]. Among the biological activities shown by this group of compounds, their capability to modulate MDR [14], their cytotoxicity against cancer cell lines [13], their anti-inflammatory activity [15,16,17,18,19] and their capability to induce proliferation or differentiation of NPC stand out with potential clinical application [4,5,20]. The relevance of the activities shown by these compounds, as well as their high content in natural sources, has allowed the development of libraries of compounds through molecular derivatization, these have been directed toward the evaluation of structure–activity relationships (SAR).

Physicochemical properties of bioactive lathyranes show a tendency toward intermediate values of molecular weight (MW = 476 ± 24.9), molecular volume (MV = 497.0 ± 30.4), logarithm of octanol/water partition coefficient (log P = 6.5 ± 0.9) and topological polar surface area (TPSA = 85.4 ± 17.6) [14]. It is important to note that their relatively high lipophilicity (logP > 5, out of the range of Lipinsky’s rule-of-five) [20] constitutes a potential handicap for their use as therapeutic agents, so the use of adequate vehicles for delivery may be needed, as is discussed later.

Tables showing the chemical structures of bioactive lathyrane diterpenoids, including those described in this review, can be found in the Supplementary Material (Sections S1–S11, including Tables S1–S11). This compilation, which covers literature published until March 2022, is organized on the basis of certain structurally characteristic deacylated derivatives, their biological activities and effects, and the molecular target on which they act (where available). The compounds described in the text are labeled in the SM with the same number with which they appear in it, while those not included have been numbered consecutively as S-1 to S-100. Where available, CAS registry numbers for compounds described in Sections S1 to S11 in the Supplementary Material have been included.

## 3. Biological Activities

### 3.1. Modulation of Multidrug Resistance (MDR)

Reversion of MDR in cancer cells by lathyrane diterpenoids has been extensively studied by investigation of their ability to modulate the transport activity of P-glycoprotein (P-gp) on different tumoral cell lines [3,7,10,14,21]. Studies have shown that not only are optimal physicochemical features, particularly lipophilicity, of major importance for MDR reversal activity, but also other factors, such as specific structural characteristics, contribute to their P-gp modulating activity [14].

**Figure 2:**
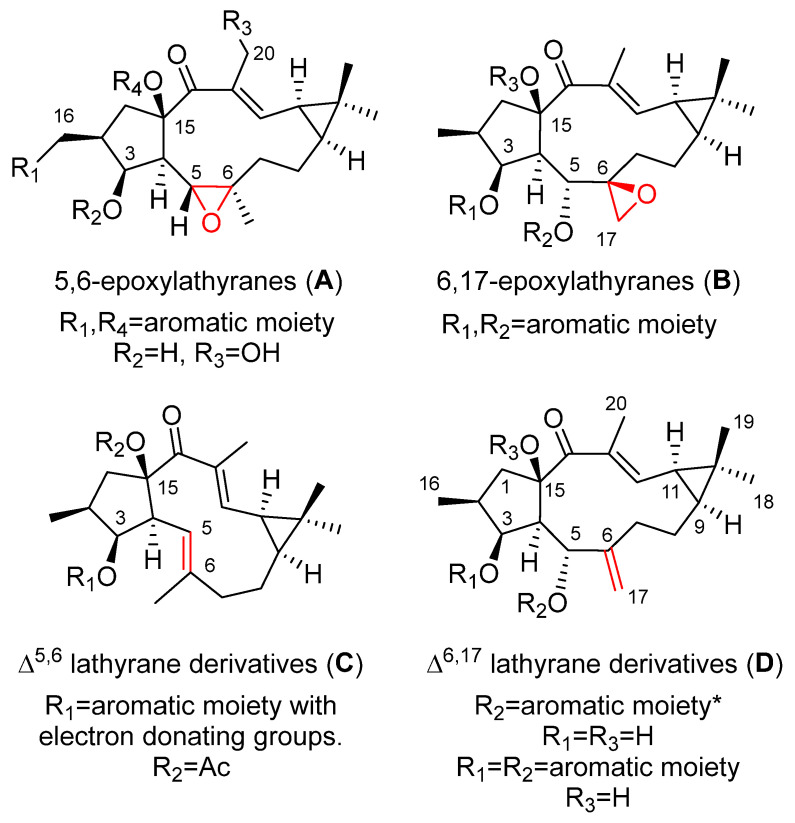
Functionalization patterns in active compounds evaluated for MDR. (**A**) Functionalization patterns in 5,6-epoxylathyranes; (**B**) Functionalization patterns in 6,17-epoxylathyranes; (**C**) Functionalization patterns in Δ^5,6^ lathyranes; (**D**) Functionalization patterns in Δ^6,17^ lathyranes. As discussed in the text, these are not absolute rules, as exceptions are observed. Both conformation of lathyrane macrocyclic scaffold and substitution patterns are determinants for MDR activity [22]. * Electron withdrawing groups and steric hindrance is detrimental for the activity of compounds evaluated against drug efflux transporters in *Candida albicans*.

Four main sets of compounds have been used to perform SAR studies: the molecules are characterized by the existence of a 5,6 or a 6,17-epoxy ring (latilagascenes A–F and jolkinol B derivatives and epoxyboetiranes, respectively; 5,6-epoxylathyranes) and 6,17-epoxylathyranes, Figure 2A,B), and those possessing an endocyclic Δ^5,6^ (jolkinol D derivatives; Δ^5,6^ lathyrane derivatives Figure 2C) or exocyclic Δ^6,17^ double bond (lathyrol derivarives; Δ^6,17^ lathyrane derivatives Figure 2D).

**Figure 3:**
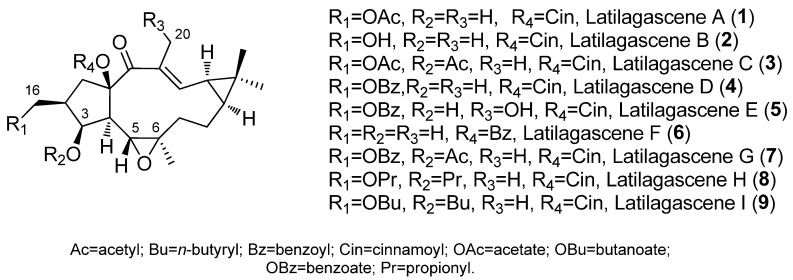
Chemical structures of latilagascene derivatives (compounds **1**–**9**) with MDR-modulating activity.

Latilagascenes A–I (**1**–**9**) (Figure 3), main representatives of the 5,6-epoxylathyranes (Figure 2A), showed to be strong modulators of P-gp efflux and exhibited concentration dependence [14]. Among them, latilagascenes D–F (**4**–**6**) were found to be the strongest modulators of P-gp when tested in human MDR1 gene-transfected mouse lymphoma cells using the rhodamine 123 exclusion test with verapamil (VRP) as the positive control [10]. Compounds **4** and **6** showed a fluorescence activity ratio R = 168.5 and 216.8 at 4 µg/mL, respectively, indicating the concentration-dependent activity of these compounds. Latilagascene E (**5**) exhibited the highest effect with R = 15.3 (4 µg/mL) while VRP showed R = 2.8 at 10 µg/mL. Comparison of the activity data demonstrated by these compounds indicated that the presence of a free hydroxyl group at C-3 is very important for this activity (Figure 3). This was demonstrated by the marked increase in activity for the latilagascene D (**4**) when compared to that of latilagascene G (**7**), which only differ by the presence of a free hydroxyl group at C-3. The substitution pattern of the pentacyclic ring also affects activity, especially the presence at C-16 of an ester group, uniquely if it is aromatic (Figure 3). For example, comparison of latilagascene B (**2**), which has two free hydroxyl groups at C-3 and C-16, and latilagacenes D (**4**) and A (**1**), which differ in the replacement of the benzoyl group at C-16 present in latilagascene D (**4**) by an acetyl group in latilagascene A (**1**), results in a decrease of activity. The presence of a hydroxyl group at C-20 also seems to be relevant for the modulation of MDR, as can be deduced from the higher activity shown by latilagacene E (**5**) when compared to that of latilagacene D (**4**). The most active 5,6-epoxylathyrane in this study was **5,** which contains two aromatic moieties at C-16 and C-15 and a free hydroxyl groups at C-3 and C-20 (Figure 3) [10]. Interestingly, a synergistic interaction was observed between doxorubicin and latilagascene B (**2**) (the most abundant of this class of diterpenes), which also bears a free OH group at C-3 and an aromatic moiety at C-15 (Figure 3). Both compounds were tested on human MDR1 gene-transfected mouse lymphoma cells, where ID_50_ for doxorubicin is 0.35 μg/mL, ID_50_ for **2** is 4.58 μg/mL, and ID_50_ for combination of both is 0.095 μg/mL, with a fractional inhibitory index of 0.292 [10].

**Figure 4:**
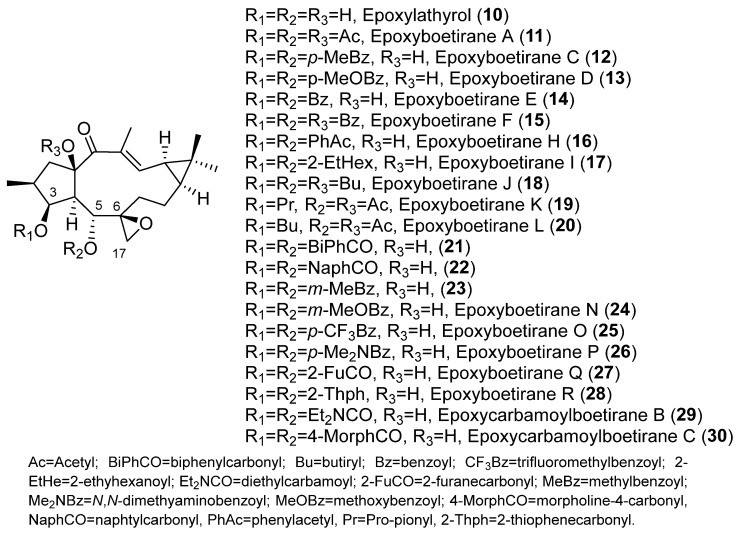
Chemical structures of 6,17-epoxyboetirane derivatives (compounds **10**–**30**) with MDR-modulating activity.

Different behavior was observed for the 6,17-epoxylathyranes (Figure 2B; see also Figure 4) in comparison with the 5,6-epoxyderivatives, where acylation pattern was of key importance, especially the presence of aromatic residues at C-3 and C-5, in P-gp modulation (Figure 2B). Interestingly, among compounds bearing aromatic moieties at C-3 and C-5, when benzoyl was replaced with phenylacetyl groups, epoxyboetiranes E (**14**), F (**15**) and H (**16**), respectively (Figure 4), increased activity was observed, suggesting that the extra methylene group may enhance the interaction with P-gp binding sites [21]. On the other hand, two of the most active compounds, epoxyboetiranes J (**18**) and L (**20**) (Figure 4), do not contain aromatic residues at these positions, which shows that, although this is important, it is not an essential factor for activity. Interaction studies with doxorubicin showed synergism for combinations with compounds **11**–**20**, with the maximum observed for epoxyboetirane K (**19**), which presented no aromatic substituents [21].

Trying to optimize epoxylathyranes as active MDR reversal agents, several compounds were isolated from the aerial parts of *E. boetica* [23]. The one obtained in greater quantity was subjected to various chemical transformations, generating a library of 16 compounds, epoxylathyrol (**10**), epoxyboetiranes (**14**, **21**–**28**) and epoxycarbamoylboetiranes B and C (**29**–**30**) (Figure 4), which were evaluated as P-gp-mediated MDR reversers at non-cytotoxic doses in L5178Y ABCB1 transfected mouse T-lymphoma cells, by the accumulation of rhodamine-123 assay [24]. Most of the tested compounds showed a strong P-gp-modulating activity and were also able to synergistically increase the cytotoxicity of doxorubicin, restoring its sensitivity by reversion of the ABCB1-MDR phenotype. Structure–activity relationship studies indicated that the presence of an aromatic ring on these structures significantly enhances the inhibition of rhodamine-123 efflux (see Figure 2B) [24].

**Figure 5:**
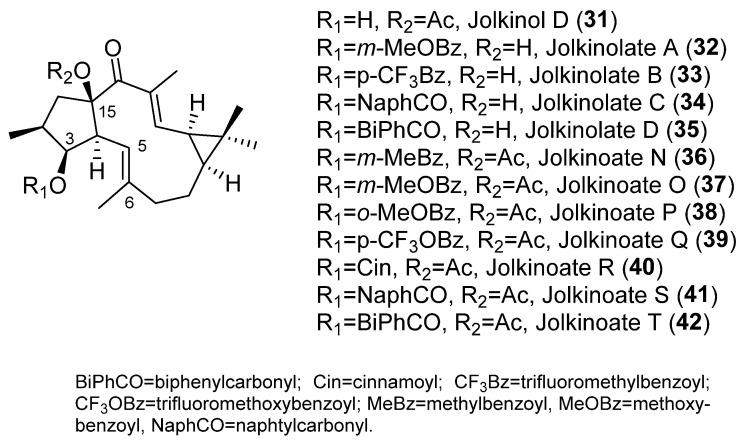
Chemical structures of jolkinol D derivatives (compounds **31**–**42**) with MDR-modulating activity.

Similar results were obtained for the jolkinol D derivatives (Figure 5), that are representative of lathyranes with a Δ^5,6^ endocyclic double bond (Figure 2C). Here, the esterification of the hydroxyl at C-3 produced an improvement in the modulating effect. For acylated alkanoyl derivatives, this increasing activity could be correlated with increasing molecular weight. However, the compounds that showed the highest activity were those with an aromatic moiety at C-3 [7], probably due to the establishment of additional hydrophobic interactions within the drug-binding pocket [7,23]. In order to investigate the effect of the presence of an aromatic residue in position C-3 of the derivatives of jolkinol D, Reis et al. generated a second generation of derivatives. Two sets of compounds were prepared, those with a free hydroxyl group at C-15 (jolkinolates A–D, **32**–**35**) (Figure 5) and those possessing an acetyl group in this position (jolkinoates N–T, **36**–**42**) (Figure 5) [8]. In both sets of compounds, electron-donating and -withdrawing groups were added to the aromatic moiety. In general, 15-acetylderivatives were more active than those possessing the C-15 free hydroxyl group and those with electron-donating groups at the aromatic moieties were the most active (Figure 2C). No significant correlations were found between the calculated physicochemical properties for these compounds and the P-gp-modulating activity, suggesting a stronger role of the sum of other structural aspects, rather than the contribution of a single physicochemical property [8]. These results reinforce the conclusion of in silico 2D and 3D QSAR studies that pointed to the conformation of the lathyrane macrocyclic scaffold and its substitution pattern (related steric and electrostatic factors) as the main determinant for MDR activity [22].

**Figure 6:**
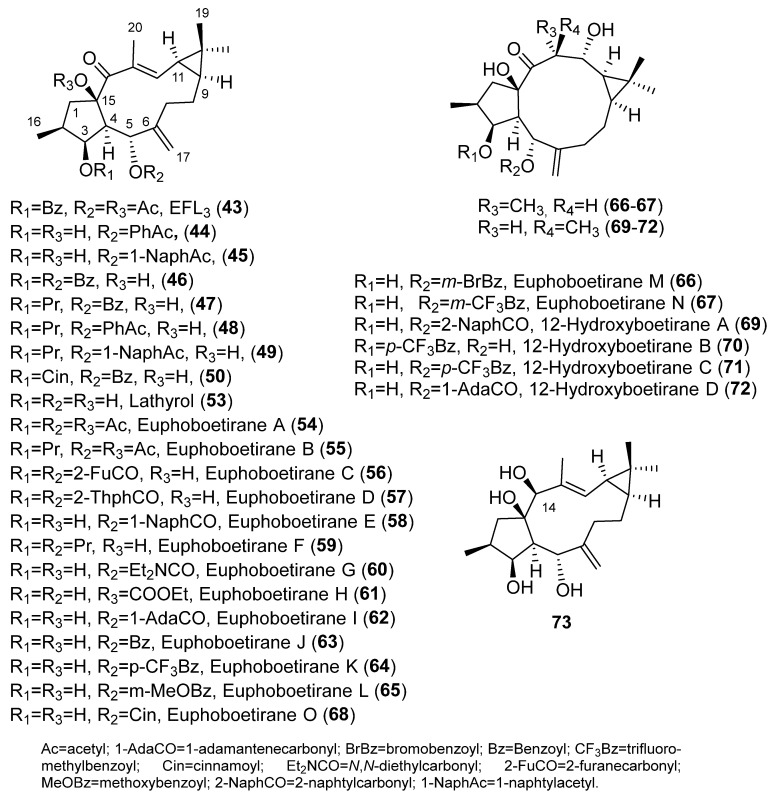
Chemical structures of bioactive lathyrol derivatives, euphoboetiranes and 12-hydroxy-boetiranes (compounds **43**–**50**, **53**–**73**) with MDR-modulating activity.

**Figure 7:**
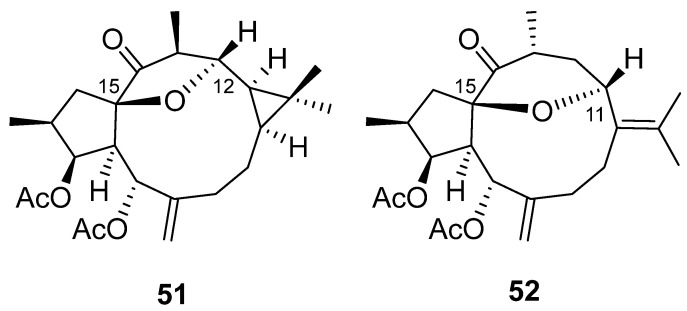
Chemical structures of compounds **51**–**52**.

Several studies on the SAR of lathyrol derivatives, which contain a Δ^6,17^ exocyclic double bond (Figure 2D), have been carried out. Jiao et al. generated a library of five series of mono or diacylated lathyranes by modifying the hydroxyl moiety of C-3, C-5 or C-15 of Euphorbia Factor L_3_ (EFL_3_, **43**) (Figure 6). This approach generated 37 compounds that were tested against breast cancer multidrug-resistant MCF-7/ADR cells that overexpress P-gp [25]. Eight derivatives **44**–**49** (Figure 6) exhibited more potent chemo-reversal ability than the positive control (VRP), and three of them (**45**, **46** and **49**) were more active than the parent structure, EFL_3_ (**43**). Comparison of the monoacylated derivatives to determine the effect of the substituent at C-5 resulted in no observed correlation between the size of aliphatic and aromatic substituents and reversal activity. Compounds with an aliphatic substituent showed poor activity profiles, while those bearing a 1-naphtylacyl or a phenylacetyl substituent at this position exhibited higher reversal fold (Figure 2D). The evaluation of lathyrol derivatives containing two ester groups at C-3 and C-5 resulted in five compounds **46**–**50** (Figure 6) exhibiting higher MDR-modulating activities than VRP. The most active compound contained two benzyl groups (**46**), resulting in 4.8 times greater effectiveness than VRP in MDR-reversal activity in MCF-7/ADR cells. It also had greater activity than all *E. lathyris* diterpenes previously evaluated [25]. A combination of propionyl and aromatic groups at C-3 and C-5, respectively, led to the second highest activity compounds (**47**, **48**, **49**). However, two bulky aromatic groups at these two positions resulted in low activity (**50**) (Figure 6). Comparing compounds with the same side chain, the activity of aliphatic substituted derivatives increased by the epoxidation of C-15 and C-12 (**51**) and decreased when the epoxy group is between C-15 and C-11 (**52**) (Figure 7) [25].

An additional study into the influence of the acylation pattern on lathyrane with an exocyclic Δ^6,17^ double bond (Figure 2D) has been undertaken by Neto et al. [26]. This group conducted a phytochemical study of *E. boetica* in which euphoboetiranes A (**54**) and B (**55**) (Figure 6) were isolated in large amounts. Starting from euphoboetirane B (**55**), 13 new derivatives, lathyrol (**53**), euphoboetiranes C–I (**56**–**62**), 12-hydroxyboetiranes A–D (**69**–**72**) and 14β-hydroxylathyrane (**73**) were prepared (Figure 6). Euphoboetirane A (**54**), euphoboetiranes C–G (**56**–**60**) and 12-hydroxyboetiranes A–C (**69**–**71**) were found to be strong P-gp modulators on L5178Y-MDR mouse lymphoma cells, when tested at 20 μM. In general, acylation of one or two of the hydroxyl groups at C-3 and C-5 (Figure 2D) of lathyrol (**53**) led to a 6 to 42-fold increase of the activity (euphoboetiranes C–G (**56**–**60**) and euphoboetirane I (**62**)). The strongest effects were exhibited by euphoboetiranes C, D and E (**56**–**58**). Conversely, reduction of carbonyl group at C-14 of lathyrol, 14β-hydroxylathyrane (**73**), led to a total loss of activity [26].

In a previous study, euphoboetiranes J–O (**63**–**68**) (Figure 6) besides epoxylathyrol (**10**) and eleven epoxyboetiranes, A (**11**), C–F (**12**–**15**), H–L (**16**–**20**) (Figure 4), were evaluated for their ability to inhibit the drug-efflux activity of Cdr1p and Mdr1p transporters of *Candida albicans* that were overexpressed in a *Saccharomyces cerevisiae* strain [27]. The products tested could be divided into three groups depending on their main structural differences: (i) those derived from epoxylathyrol, characterized by having a 6,17-epoxy function (**10**–**20**) (Figure 2B); (ii) those derived from lathyrol that contain two double bonds, Δ^6,17^ (Figure 2D) and Δ^12,13^ (**63**–**65, 68**) (Figure 6); (iii) and those characterized by the absence of the endocyclic Δ^12,13^ double bond with an extra hydroxyl function at C-12, euphoboetiranes M and N (**66**–**67**) (Figure 6). Euphoboetiranes J–O (**63**–**68**) showed the strongest inhibitory activity of Cdr1p efflux pump, while the most active compounds in *S. cerevisiae* cells overexpressing Mdr1p were epoxylahyrol (**10**) > epoxyboetirane J (**18**) > epoxyboetirane E (**14**) > epoxiboetirane D (**13**) > epoxyboetirane A (**11**). According to the results of the study, the inhibitory activity on both proteins seemed to be associated to the type of substituent at C-6 [27]. Therefore, euphoboetiranes J–O (**63**–**68**) showed a variable inhibitory potential. The highest activity was achieved for euphoboetirane J (**63**), which has an unsubstituted benzoyl moiety, while the substitution of the benzene ring with a strong electron-withdrawing effect (euphoboetirane K, **64**) seems to be detrimental to the inhibitory activity. On the other hand, steric hindrance seems to be the reason why euphoboetirane O (**68**), bearing a cinnamoyl group at C-5, showed low activity, probably due to a poor interaction with the protein binding sites. Finally, the presence of an extra hydroxyl group at C -12, euphoboetiranes M and N (**66**–**67**), did not have a significant effect on the activity. Comparison of general physicochemical properties and the inhibitory activity for the three groups of compounds indicated that a preferential log P value, between 3.11 and 4.16, is required for a good inhibitory activity of the ABC-transporter Cdr1p. Nevertheless, no significant correlations were found between the calculated physicochemical properties and AD-MDR1 inhibitory activity, suggesting that other factors, such as the particular structural features of the compound, played the strongest role [27].

**Figure 8:**
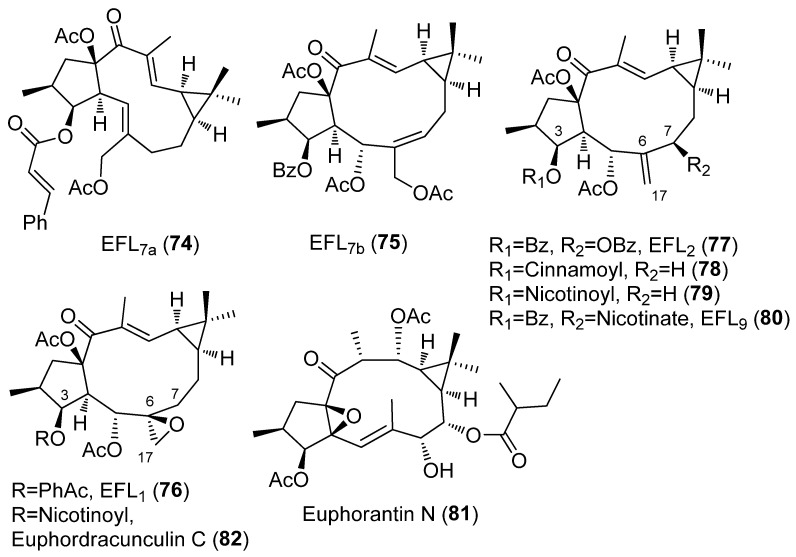
Chemical Structures of lathyranes from *E. lathyris* L. with MDR-modulating activity (compounds **74**–**82**).

A study comparing the MDR inhibition activity of different types of lathyrane diterpenoids, all of them isolated from *E. lathyris*, was conducted by Jiao et al. [28]. Six compounds, belonging to jolkinol and isolathyrol groups (EFL_7a_, **74** and EFL_7b,_ **75,** respectively, Figure 8) characterized for an endocyclic double bond, Δ^5,6^ and Δ^6,7^, respectively, to the epoxylathyrol group with a 6,17-epoxy ring (EFL_1_, **76**), and to 7-hydroxylathyrol (EFL_2_, **77**) and lathyrol groups (EFL_3_, **43** and **79**), which possess an exocyclic Δ^6,17^ double bond, (Figure 6 and Figure 8, respectively), were tested as modulators of multidrug resistance using MCF-7/ADM cell lines in vitro. According to the results, the position of the double bond at C-5 (EFL_7a_, **74**) or C-6 (EFL_7b_, **75**) and endo or exocyclic (**75** or **43** and **79**, respectively) is the key element responsible for inhibitory activity. Furthermore, when the double bond changes to epoxide (**43** to **76**), the effect decreases, although the influence of the change of position of the double bond is bigger. An additional very important factor is the substitution of C-7. The approximate sequence of different skeletons as MDR modulator was 7-hydroxylathyrol, jokinol > lathyrol >epoxylathyrol > isolathyrol [28].

Recently, Yang et al. also evaluated the reversing MDR activity of HepG2/ADR cells of twenty-three diterpenoids isolated from the seed of *Euphorbia lathyris* L. [29]. The study again included three of the four sets of characteristic lathyrane derivatives: 6,17-epoxylathyranes, and lathyranes with endocyclic Δ^5,6^ or exocyclic Δ^6,17^ double bond (Figure 8). Three of them (5,15-diacetoxy-3-nicotinoyloxylathyra-6 (17),12-dien-14-one (**79**), 5,15-diacetoxy-3-benzoyloxi-7-nicotinoyloxylathyra-6 (17),12-dien-14-one (**80**) and 3,12-O-diacetyl-8-O-(2-methyl)butyrilingol (euphorantin N, **81**)) were more potent than positive control VRP [29]. Loss of activity is observed if a 6,17-epoxide is present, as shown by the comparison of compound **79** (most potent component in above series), and its epoxide at Δ^6,17^ double bond (compound **82**), confirming the trend observed by Jiao et al. [28]. Conversely, loss of activity is also observed when the nicotinyl subtituent on C-3 in compound **79** is replaced by a cinnanoyl (compound **78**) [29].

#### Mode of Action

Resistance to anticancer drugs is a complex process that can include more than one MDR mechanism [30]. One of the most significant is the overexpression of ATP-binding cassette (ABC) transporters, a family of proteins that mediate MDR via ATP-dependent drug efflux pumps [31]. P-gp is the most typical efflux pump in the cell membrane. The principal strategy used to overcome MDR is the development of P-gp modulators that, when co-administered with an anticancer drug, avoid its efflux and prevent chemotherapy failure [32].

**Figure 9:**
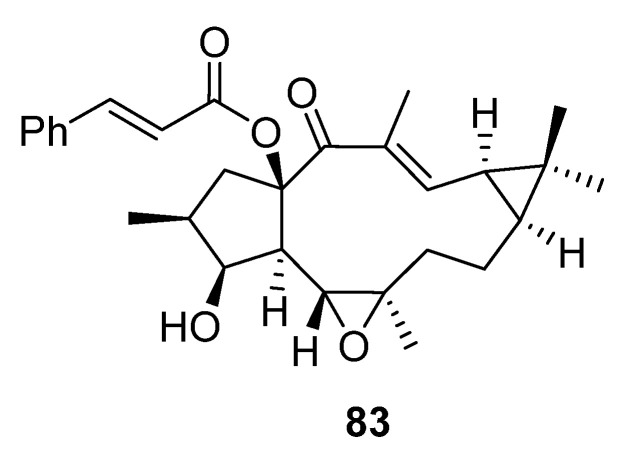
Chemical Structure of jolkinol B or EM-E-11-4 (**83**).

P-gp inhibition may proceed through competitive (direct interaction with drug-binding sites), non-competitive or allosteric (indirect inhibition of P-gp through conformational changes which inhibit activity and translocation of protein) mechanisms. For instance, EM-E-11-4 (jolkinol B, **83**) (Figure 9) does not change P-gp expression levels, but suppresses ATPase activity, which indicates a non-competitive inhibition mechanism for this lathyrane [33].

On the other hand, a recent study on the paclitaxel resistance-reversing activity of the 5,6-epoxylathyrane **83** (jolkinol B, Figure 9) has pointed at the presence of multiple mechanisms of action, where not only P-gp is involved, but also inhibition of class III β-tubulin [33]. The relevance of β-III-tubulin overexpression in connection with resistance to paclitaxel in tumors has been described [34]. β-III-tubulin has the ability to counteract the stabilizing effect of paclitaxel and other microtubule interacting agents on the microtubules dynamic nature [35], avoiding the mitotic arrest (G2/M phase arrest) which leads to cell apoptosis associated with the action of microtubule stabilizing agents such as paclitaxel [36,37]. β-III-Tubulin is capable of induction of resistance to paclitaxel and other drugs, resulting in promotion of tumor survival [36]. Compound **83** worked synergistically with paclitaxel, promoting tubulin from soluble to insoluble states and increased binding of paclitaxel to microtubules. The authors suggested that **83** may bind in the proximity of the paclitaxel binding domain, resulting in protein conformation change and enhancing paclitaxel-mediated tubulin polymerization and its binding to microtubules [33].

The potential mechanism by which compound **79** (Figure 8) regulates P-gp-dependent MDR was also studied. The results show that **79** did not influence the P-gp expression and did not inhibit the transcription and translation process, but it induced the amount of P-gp monomer in a time-dependent style [29].

**Figure 10:**
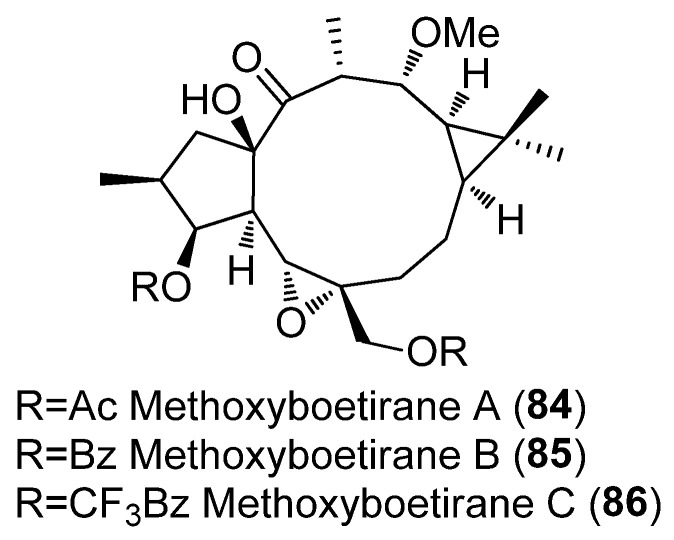
Chemical Structures of methoxyboetiranes A–C (**84**–**86**).

In a recent study aimed at considering other potential anti-MDR mechanisms of action, the epoxylathyrane derivatives, epoxylathyrol A (**10**), epoxiboetiranes (**14**, **21**–**28**), epoxycarbamoylboetiranes B and C (**29**–**30**) (Figure 4) and methoxyboetiranes A–C (**84**–**86**) (Figure 10) were investigated for their potential as collateral sensitizing compounds. This was achieved using drug-sensitive and drug-resistant sublines of human tumor gastric (EPG85-257), pancreatic (EPP-181) and colon (HT-29) cell models [38]. The compounds tested were found to be more effective against the resistant gastric cell line, resulting in epoxyboetirane P (**26**) and methoxyboetiranes B (**85**) and C (**86**) being the most promising compounds, which were additionally investigated as apoptosis inducers. The collateral sensitivity effect elicited by methoxyboetiranes **85** and **86** seemed to be due to the induction of apoptosis via caspase-3 activation [38].

### 3.2. Cytotoxic

**Figure 11:**
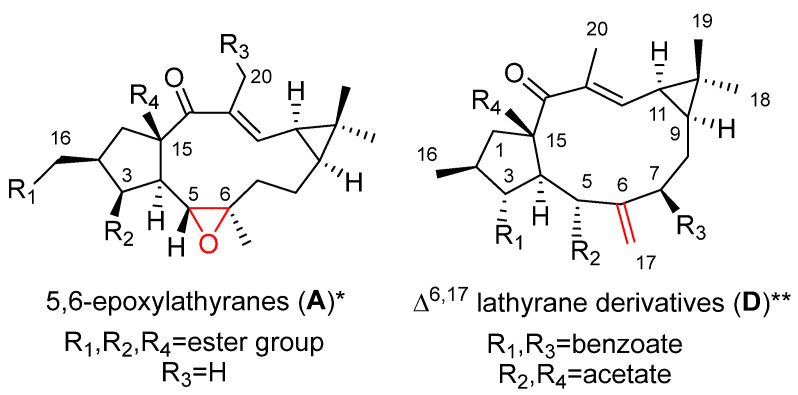
Selected functionalization patterns in active Ingol tupe diterpenes evaluated for their activity against cancer cell lines. (**A**) Functionalization pattern in 5,6-epoxylathyranes; (**D**) Functionalization pattern in Δ^6,17^ lathyranes. Due to the variety of the cell lines investigated and the reduced number of compounds explored against each cell line, no further comparison can be drawn. * Patterns for active compounds evaluated against multidrug-resistant EPG85-257RDB cells. ** Patterns for active compounds evaluated against KB-VIN cells.

The cytotoxic activity of 5,6-epoxylathyranes (Figure 11A) has been extensively studied, the substitution pattern of the A ring influences the antiproliferative activities for this group of lathyranes, as well as the necessity for the presence of a hydroxyl group at C-20.

This was observed in studies with latilagascenes B (**2**), C (**3**) and D (**4**) (Figure 3) and jolkinol B (**83**) (Figure 9) undertaken on several human cancer cell lines that were derived from three different tumor entities: gastric (EPG85-257), pancreatic (EPP85-181) and colon cancer cells (HT-29) [39]. The activity demonstrated against gastric carcinoma was found to be dependent on the individual drug-resistant phenotype. Latilagascenes C (**3**) and D (**4**) were found to be more effective than the positive control etoposide in the drug-resistant subline EPG85-257RDB. This is associated with the overexpression of the ABC transporter MDR1/Pgp, and latilagascene B (**2**) also exhibited a significant activity; however, jolkinol B (**83**) showed a moderate activity. On the other hand, latilagascenes B–D (**2**–**4**) had moderate activity in MRD EPG85-257RNOV cells associated with altered topoisomerase II expression, while jolkinol B (**83**) exhibited a significant antineoplastic activity. None of these compounds showed significant activity against any of the three sublines of the pancreatic carcinoma cells (EPP85-181), resulting in latilagascene D (**4**) being inactive. Comparable results were obtained for the colon carcinoma cells [39]. Compounds **2**–**4** and jolkinol B (**83**) only differed in their substitution pattern of the pentacyclic ring (ring A, Figure 1). Latilagascenes B (**2**) and D (**4**) and jolkinol B (**83**) have a free hydroxyl group at C-3, which is acetylated in latilagascene C (**3**). Jolkinol B (**83**) is not oxidized at C-16, while latilagascene B (**2**) has a free hydroxyl group at this position that in latilagascenes C (**3**) and D (**4**) is esterified with an acetate and benzoylate, respectively. Comparison of the results obtained in EPG85-257 cells for these compounds suggested that the esterification of hydroxyl groups at C-3 and C-16 is important for the cytotoxic activity in multidrug-resistant EPG85-257RDB cells (Figure 11), since the presence of free hydroxyl groups at these positions decreased the activity. Additionally, oxidation at C-16 seems to also be a relevant structural requirement for the activity, as Jolkinol B (**83**), containing a methyl group at C-16, was the most active compound tested in multidrug-resistant EPG85-257RNOV cells, but the least active in multidrug-resistant EPG85- 257RDB cells [39].

**Figure 12:**
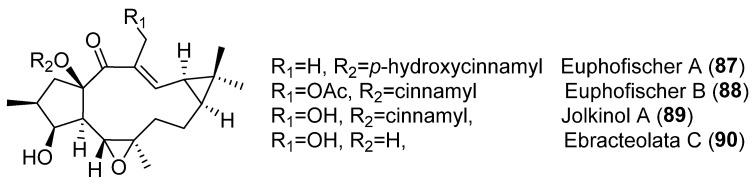
Cytotoxic lathyranes diterpenoids (**87**–**90**) from the roots of *E. fischeriana*.

The effect of Latilagacenes A–E (**1**–**5**) (Figure 3) and jolkinol B (**83**) (Figure 9) on human cytomegalovirus (CMV) IE antigen expression in lung cancer cells has also been investigated. Latilagascene E (**5**) was found to have the highest activity, while latilagascene D (**4**) was inactive [40]. Comparison of the activity of latilagascene D (**4**) and A (**1**), whose structures differ only at the ester group at C-16, suggests that the presence of the benzoyl moiety has a negative effect on the inhibitory of IE antigen expression of CMV. Comparatively, the activity of latilagascene D (**4**) and E (**5**) confirmed that the presence of a hydroxyl group at C-20 appears to be important in the antitumor promoter activity of these compounds [40]. However, the opposite result was obtained when the cytotoxic activity of another set of 5,6-epoxylathyranes, the euphofischers A (**87**) and B (**88**), jolkinol A (**89**) (Figure 12) and B (**83**) (Figure 9) and ebracteolata C (**90**) (Figure 12) was tested. They showed moderate activities against human prostate cancer cell lines C4-2B, as well as the enzalutamide-resistant cell line C4-2B/ENZR. Weak activity was observed against the human breast cancer cell line MDA-MB-231. Euphorfischer A (**87**), a rare example of a lathyrane diterpenoid featuring a 15-*p*-coumaroyl moiety and the only one that does not have a hydroxyl group at C-20, was found to be the most active, exhibiting significant toxicity against C4-2B cell line with an IC50 value of 11.3 μM [41].

In a previous study with diterpenes from *E. fischeriana*, jolkinol A (**89**) (Figure 12), the inhibitory activity on the formation of mammospheres in human breast cancer MCF-7 cells was observed [42]. Furthermore, jolkinol B (**83**) (Figure 9) was isolated from the roots of *E. ebracteolata* Hayata, and its cytotoxic activity tested against five cancer cell lines: HL-60 (human promyelocytic leukemia cell line), SMMC-7721 (human hepatocellular carcinoma cell line), A-549 (human lung cancer cell line), MCF-7 (human breast cancer cell line) and SW480 (colorectal cancer cell line), where it exhibited moderate cytotoxic effects [43].

**Figure 13:**
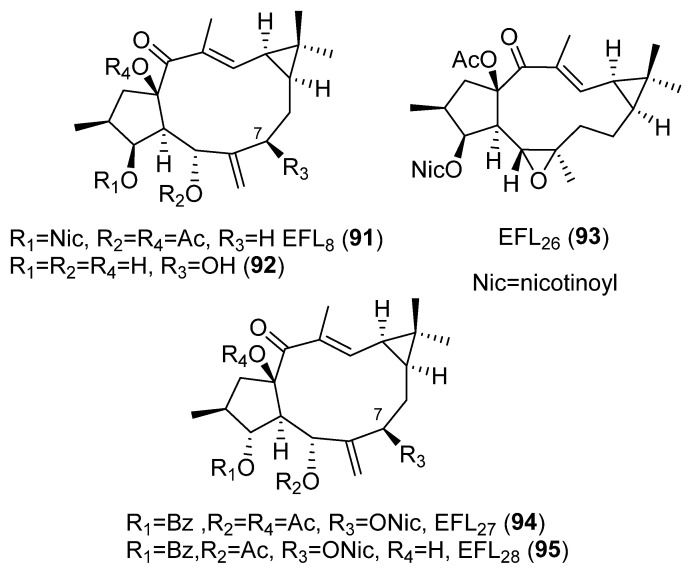
Chemical structures of compounds **91**–**95**.

Lathyrane diterpenoids isolated from *E. lathyris*, named Euphorbia factors (EF), have been subjected to several anticancer studies. Thus, among the cytotoxicity against cancer cell lines A549, MDA-MB231, KB and MCF-7, and the MDR cancer cell line KB-VIN of six compounds with different structural features, EFL_1–3_ (**76**, **77** and **43**) (Figure 6 and Figure 8), EFL_8–9_ (**91, 80**) (Figure 8 and Figure 13) and the tetraol derivarive of **91** (compound **92**) (Figure 13), EFL_9_ (**80**) exhibited the strongest activity against all tested cell lines and EFL_2_ (**77**) was found to be selective against KB-VIN, while **76** and **92** were inactive. The SAR studies revealed that the substitutions at C-3, C-5, C-7 and C-15 are critical for cytotoxicity, as well as cell type-selectivity [44]. A combination of acetate groups at C-5 and C-15 and benzoate groups at C-3 and C-7 seems to be required for selective cytotoxicity against KB-VIN (Figure 11D).

In comparison, the cytotoxic activity of EFL_1–3_ (**76**, **77** and **43**) (Figure 6 and Figure 8), EFL_7a_ (**74**) (Figure 8), EFL_8_ (**91**) and 7-hydroxylathyrol (**92**) (Figure 13), EFL_9_ (**80**) (Figure 8) and EFL_26_ (**93**) (Figure 13), together with other lathyrane-type diterpenes isolated from *E. lathyris*, EFL_27_ (**94**) and EF_28_ (**95**) (Figure 13), which are characterized for an α-orientation of their substituent at C-3, was also evaluated against several breast cancer cell lines. Results showed a strong cytotoxicity associated with **95** against the 786-0 and HepG2 cell lines. This indicated that not only the variety but also the configuration of substituent groups in this kind of compounds are of great importance for their bioactivity [45].

**Figure 14:**
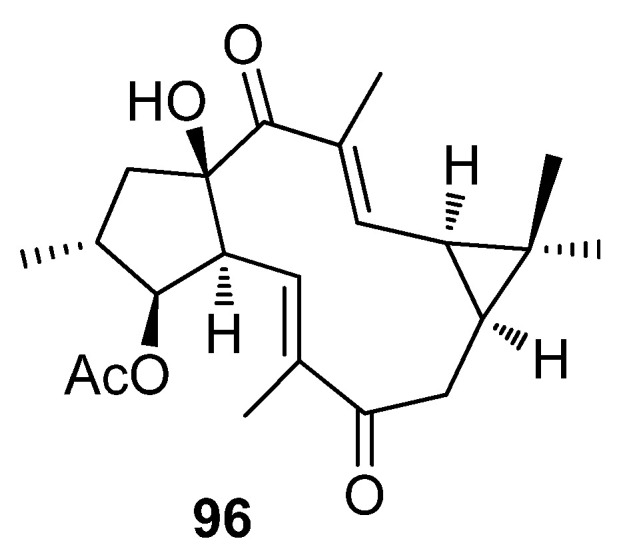
Structure of jatropodagin A (**96**).

In a recent study looking at the composition of stems of *Jatropha podagrica*, a set of lathyrane-type diterpenoids were isolated and their antitumor activities in two human osteosarcoma cell lines (MG-63 and Sais-2) were evaluated [46]. Only one, jatropodagin A (**96**, Figure 14), had significant cytotoxic activity with IC50 values of 8.08 and 14.64 μM, against Saos-2 and MG63 cell lines, respectively.

**Figure 15:**
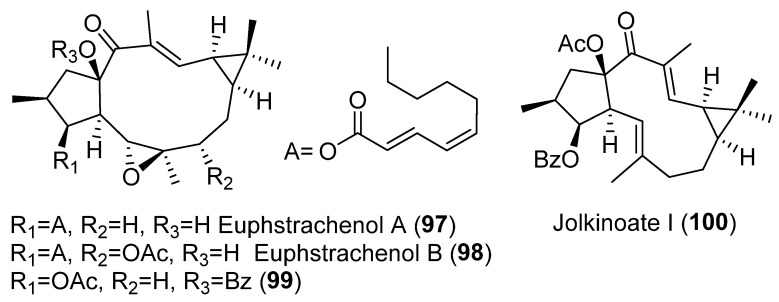
Cytotoxic lathyrane-diterpenes (**97**–**100**) from *E. stracheyi*.

A study into the composition of the perennial herb *Euphorbia stracheyi*, two new lathyrane diterpenoids, euphstrachenols A and B (**97**–**98**) and nine analogues were isolated and identified from the methanol extract of its roots (Figure 15). The evaluation of their cytotoxicity against four human cancer cell lines, HGC-27 (stomach cancer), MV4-11 (leukemia), H460 (lung cancer), Skvo3 (ovarian cancer) and a murine cell line BaF3 (lymphocyte), indicated that all of them showed cytotoxicity against H460 and Skvo3 cell lines, but only six of them, euphstracehnols A (**97**) and B (**98**), **99** (Figure 15), EFL_15_ (euphoboetirane A, **54**) (Figure 6), jolkinoate I (**100**) (Figure 15) and jolkinol B (**83**) (Figure 9) indicated moderate cytotoxicity against MV4-11 cell lines [47].

**Figure 16:**
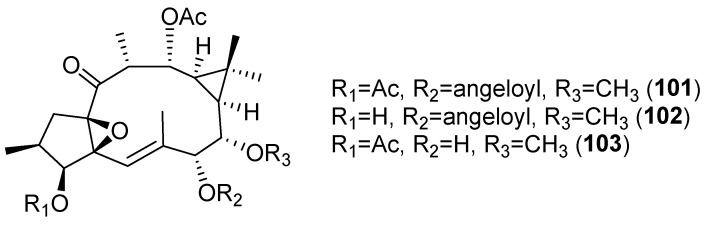
Ingol-type diterpenes (**101**–**103**) with cytotoxic activity.

The cytotoxic activity of ingol-type diterpenes has also been evaluated. 3,12-diacetyl-7-angeloyl-8-methoxyingol (**101**), 7-angeloyl-12-acetyl-8-methoxyingol (**102**) and 3,12-diacetyl-7-hydroxy-8-methoxyingol (**103**) (Figure 16), isolated from *E. nivulia*, showed significant cytotoxic activity against Colo 205, MT2 and CEM cell lines, although some ingol derivatives had little or no activity [48].

#### Mode of Action

It is well documented that there is a direct relationship between resistance to cancer chemotherapy and P-gp expression [49,50,51]. One of the approaches that is being developed to overcome resistance to anticancer drugs is development of alternative drugs without cross-resistance in cancer cells exhibiting a drug-resistant phenotype [32].

Several macrocyclic diterpenes with a latilagascene skeleton (Figure 11A) have been shown to have very strong modulation of P-gp activity in resistant cancer cells, as well as apoptosis induction activity in human MDR1 gene-transfected mouse lymphoma cells [10,40,52]. Latilagascenes A (**1**), B (**2**) and C (**3**) (Figure 3), isolated from *E. lagascae*, had the ability to inhibit rhodamine 123 efflux of human MDR1 gene-transfected mouse lymphoma cells. Latilagascene B (**2**) was tested in combination with doxorubicine and this showed a synergistic interaction in the same resistant cell line [53]. In contrast, latilagascenes C (**3**) and D (**4**) were highly effective against the drug-resistant subline EPG85-257RDB (associated with the overexpression of the ABC transporter MDR1/P-gp) derived from gastric carcinoma. These showed moderate activity in multidrug-resistant EPG85-257RNOV cells associated with altered topoisomerase II expression [39]. However, the macrocyclic lathyrane diterpene jolkinol B (**83**) (Figure 9) showed significant antineoplastic activity against this multidrug-resistant variant, suggesting that the activity of these compounds depends from the individual drug-resistant phenotype.

The mechanism of action in KB-VIN cells of EFL_1–3_ (**76**, **77** and **43**) (Figure 6 and Figure 8) and EFL_8–9_ (**91**, **80**) (Figure 8 and Figure 13) was determined. Two different modes of action seem to be present. EFL_3_ (**43**) and EFL_9_ (**80**) acted disrupting normal cell cycle progression, whereas EFL_2_ (**77**) and EFL_8_ (**91**) induced both actin filament aggregation, as well as partial disruption of microtubule networks [44].

A study into the reversal activities of EFL_1_ (**76**) (Figure 8) against ABCB1-mediated MDR and apoptosis sensitization in K562/ADR cell was conducted by Zhang et al. in 2013 [52]. EFL_1_ (**76**) elevated sensitivity to chemotherapeutical drugs in ABCB1-mediated MDR K562/ADR cells and did not affect the sensitivity of K562, KB and MCF-7 cells to chemotherapeutic agents. The results suggest that **76** combined with chemotherapeutic drugs might be useful to overcome multidrug resistance. The study of the mechanism of action revealed that the mitochondrial pathway is involved in the apoptosis induced by EFL_1_ (**76**) [52,54].

Studies on the cytotoxic activity of EFL_2_ (**77**) (Figure 8) and EFL_3_ (**43**) (Figure 6) revealed that the antiproliferative activity in vitro shown by these compounds against lung cancer A549 cells was also mediated by apoptosis induction, via a mitochondrial pathway [55,56]. Besides, treatment of A549 cells with **43** induced release of cytochrome *c* in a time-dependent manner, indicating a mitochondrially mediated pathway resulting in apoptosis, presumably via Caspase 9 activation that produces the activation of the executioner Caspase 3 [55]. Similar results were obtained in a study with **77**, in which it was also found that there was an increase in ROS generation, activation of caspase-9 and caspase-3 and the cleavage of FF, reinforcing the hypothesis that apoptosis of A549 cells is produced through a mitochondrial pathway [56]. On the other hand, EFL_2_ (**77**) has a potent effect on hepatocellular carcinoma (HCC) and the study of its mode of action suggested that **77** inhibited TGF-β-induced migration and proliferation in HCC cells through the inhibition of phosphorylation of AKT and STAT3 [57].

The apoptosis-inducing activity of latilagascene A–D (**1**–**4**, Figure 3) and jolkinol B (**83**) (Figure 9) in human MDR1 gene-transfected mouse lymphoma cells has been tested. Analysis of the observed effect allowed the conclusion that these lathyrane diterpenes can not only be considered effective anti-MDR agents, but also as apoptosis inducers, reinforcing the importance of them as antitumor agents [10].

Apoptosis also turned out to be the mode of action that mediates the antiproliferative effect of jatropodagin A (**96**) (Figure 14). This was confirmed by analyzing the morphological changes observed in saos-2 cells treated with this compound. Jatropodagin A (**96**) treatment caused significant morphological changes, including the appearance of membrane blebbing and granular apoptotic bodies [46].

**Figure 17:**
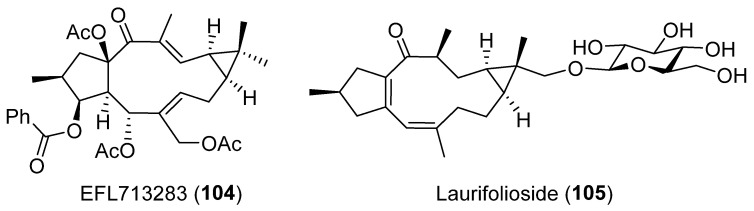
Structure of EFL713283 (**104**) and laurifolioside (**105**).

A lathyrane designated EFL713283 (**104**) (Figure 17), isolated from *E. lathyris*, showed a strong anticancer activity. Using integrated in silico methods, the possible targets of compound **104** were explored. These studies indicated that the potential target of EFL713283 (**104**) might be β-tubulin, suggesting an anticancer mechanism similar to that of Taxol. Compound **104** binds to β-tubulin favoring the formation of α, β-tubulin dimmer [58].

A different mode of cytotoxic activity was found with laurifolioside (**105**) (Figure 17). This was found to be active against human prostate cancer (PC-3) and human breast adenocarcinoma (MCF-7) cell lines. A Drug Affinity Responsive Target Stability (DARTS) strategy showed that Clathrin heavy chain 1, a protein mainly involved in selective uptake of proteins, viruses and other macromolecules at the plasma membrane of cells, is the main target for laurifolioside (**105**) [59].

### 3.3. Anti-Inflammatory Activity

Inflammation is considered to be the body’s normal response to defend itself against pathogens and injuries, but excessive inflammation can affect the normal function of tissues and organs, leading to chronic diseases and sometimes the development of cancer [60,61]. To mitigate its effects, the body activates the immune system by recruiting immune cells and antibodies [62]. Nitric oxide is a critical signaling molecule and is considered to be the regulator of many physiological mechanisms [63]. When the immune system is chronically or overly activated, NO and inflammatory mediator cytokines such as IL-1β and IL-6 are released, which have been shown to be closely related to inflammation [64]. Pharmacological research has proven that overproduction of nitric oxide (NO] is indicative of an inflammatory process. For example, NO is over-produced and secreted out of mouse macrophages in response to bacterial lipopolysaccharide (LPS) [65]. Consequently, the most general way to prove the anti-inflammatory activity of a compound is to measure its ability to inhibit NO production.

**Figure 18:**
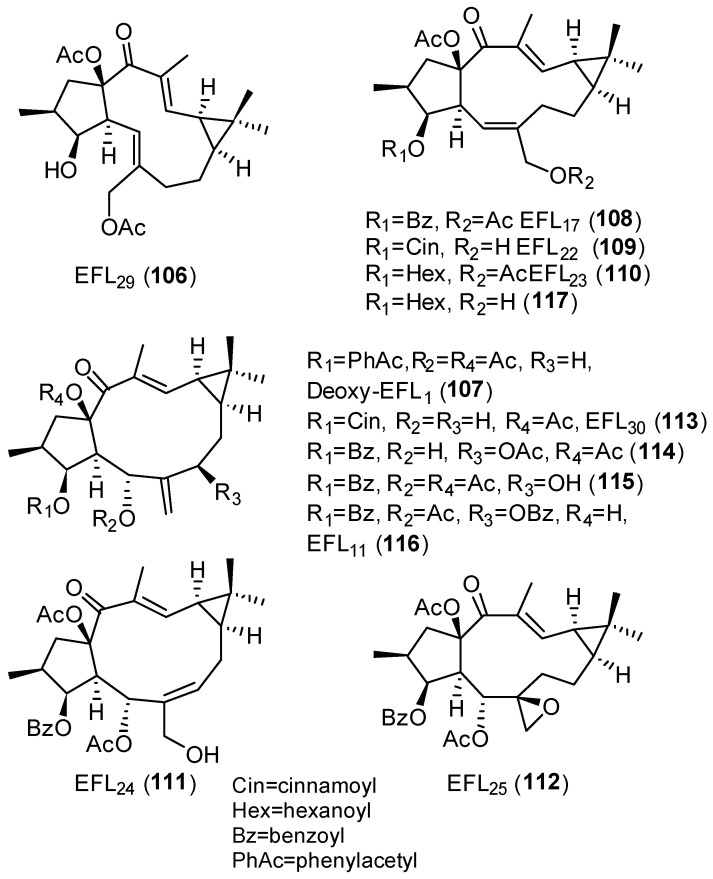
Structure of compounds **106**–**117**.

**Figure 19:**
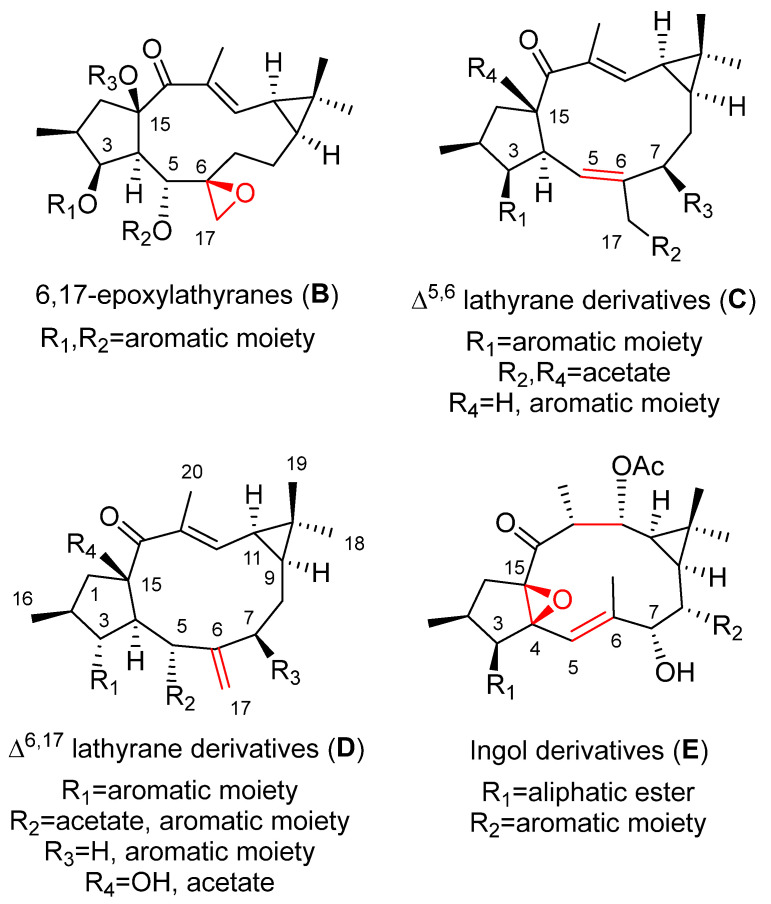
Selected functionalization patterns in biologically active compounds evaluated for their anti-inflammatory effects. (**B**) Functionalization pattern in 6,17-epoxylathyranes; (**C**) Functionalization pattern in Δ^5,6^ lathyranes; (**D**) Functionalization pattern in Δ^6,17^ lathyranes; (**E**) Functionalization pattern in ingol derivatives.

Twenty-one compounds, **11** (Figure 4), **43** (Figure 6), **74**–**77** and **80** (Figure 8), **91** and **93** (Figure 13), **89** (Figure 12) and **106**–**117** (Figure 18), belonging to several groups of lathyranes, were found to inhibit the nitric oxide production in LPS-induced RAW 264.7 macrophages; nevertheless, no significant SAR could be established [18,66]. A subsequent study by Zhang et al. did allow the establishment of some essential structural characteristics for the NO production inhibitory activity. In their study, they used three different sets of compounds bearing a distinct substitution pattern: exocyclic Δ^6,17^ double bond (see Figure 19D), endocyclic Δ^5,6^ (Figure 19C) or Δ^6,7^ double bond (isolathyrol, (**S47**, Supplementary Material, Section S6)) and endocyclic (Figure 2A) or exocyclic (Figure 19B) epoxy function [16]. Those compounds with an exocyclic Δ^6,17^ double bond (**43**, **113**–**116, 80**) (Figure 6, Figure 18 and Figure 8, respectively) were the most active, showing a significant inhibitory effect higher than those showed for those with a 5α,6β-epoxy (Figure 2A) or Δ^5,6^ (Figure 19C) or Δ^6,7^ (**S47**, Supplementary Material, Section S6) double bonds. In contrast, compounds similar to **80** without a nicotinoyl group on C-7 were inactive, indicating a nitrogen-containing aromatic group at C-7 is probably critical for the inhibition of NO production. Furthermore, the acetylation at C-15 in **80** canceled the inhibitory effect. Comparing the activity of the compounds with a Δ^5,6^ double bond, only those bearing an aliphatic moiety at C-3 and a free hydroxyl group at C-17, such as **117**, showed significant activity. Finally, compound **76** with a 6,17-epoxy moiety showed moderate inhibitory activity [16]. Compound **91**, described as active by Lee el al. [18], was found to be inactive.

**Figure 20:**
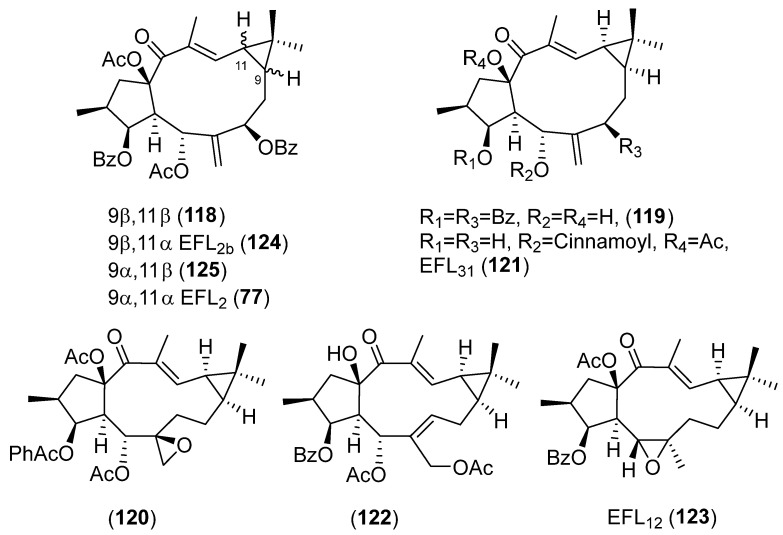
Structure of compounds **77**, **118**–**125**.

In a recent study, Zuo et al. evaluated the inhibitory activity against NO production induced by LPS in BV-2 microgial cell of twenty-one lathyrane diterpenoids [17]. Seventeen compounds, **43** (Figure 6), **74, 75** and **77** (Figure 8), **91** (Figure 13), **107**–**110** and **113** (Figure 18) and **118**–**125** (Figure 20), that demonstrated low cytotoxicity were found to be significantly active against LPS-induced NO overproduction in BV-2 microglial cells at 10 µM. Compounds **120** and **43** were the most potent (respectively, 6,17-epoxylathyrane (**B**) and Δ^6,17^ lathyrane (**D**) derivatives, Figure 19), showing an inhibitory effect approximately twice as active as the positive control resveratrol (20 µM) and reducing markedly the mRNA levels of pro-inflammatory cytokines IL6 and IL1β in LPS-stimulated BV2-cells. Structures **93** (Figure 13), **118** and **124** (Figure 20) were effective at the non-toxic concentration 1 µM, indicating that their effective concentration was lower than other tested compounds. Following the comparison of the activity shown by compounds **118**, **124**, **125** and EFL_2_ (**77**) (Figure 20), which are isomeric compounds in C-9 or/and C-11, it can be deduced that the anti-inflammatory activity depends on the configurations at C-9 and C-11 [17].

**Figure 21:**
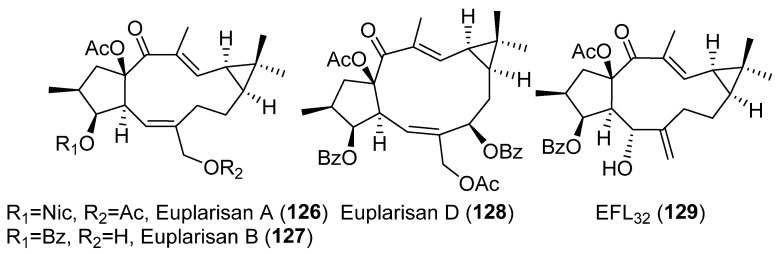
Structure of compounds **126**–**129**.

A systematic study of the anti-inflammatory activity of different types of lathyrane has been carried out by Wang et. al. [19]. Eleven new lathyranes along with ten known analogues, isolated from *E. lathyris*, were evaluated for their inhibitory activities against NO production induced by LPS in RAW264.7 macrophage cells. The tested compounds possess different patterns of substitution on the macrocyclic diterpene skeletons, including 17-hydroxyjolkinols, 17-hydroxyisolathyrols (two of them with an unusual *trans*-*gem*-dimethylcyclopropane unit), lathyrols (Figure 19D) and epoxylathyrols (Figure 19B). The most active compounds were found to be four 17-hydroxyisolathyrol derivatives, euplarisan A, B and D (**126**–**128**) (Figure 21), EFL_17_ (**108**) (Figure 18) and two lathyrols, EFL_28_ (**95**) (Figure 13) and EFL_32_ (**129**) (Figure 21). Comparison of the activity showed for all the tested compounds that the diterpenoids with an endocyclic Δ^5,6^ double bond (**108** and **128**) and a benzoate at C-3 have the highest inhibitory effect (Figure 19C) [19].

**Figure 22:**
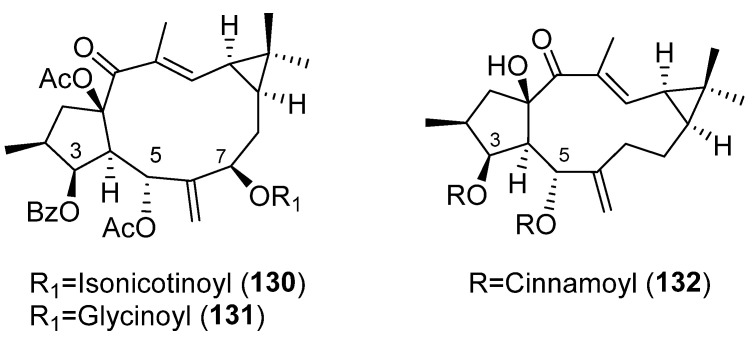
Structure of compounds **130**–**132**.

The influence of different positions and type of substitution on anti-inflammatory efficacy of EFL_3_ (**43**) (Figure 6) was explored, building a library of compounds through the allylic hydroxylation at C-7 with subsequent esterification with fatty acids, substituted benzoic acids, cinnamic acid and heterocyclic acids. In addition, compounds bearing one or two of these chains at C-3 and/or C-5 were also synthesized [15]. In the first set of compounds, when the C-7 hydroxyl group was esterified, the inhibitory activity demonstrated by many derivatives was weaker than those showed by **43,** being comparable or even better in those compounds with a nicotinic (**80**) (Figure 8), isonicotinic acids (**130**) or glycine at C-7 (**131**) (Figure 22). The second set of compounds displayed better activity than the first one. These were compounds **46** (Figure 6) and **132** (Figure 22) (Δ^6,17^ lathyrane, Figure 19D). The preliminary SAR showed that the esterification of the C-5 with aromatic groups is important for improving the anti-inflammatory activity of the lathyrol scaffold. When the benzene ring is substituted or changed into a heterocycle, the inhibitory activity is decreased. Higher activity is observed when both hydroxyl groups at C-3 and C-5 are esterified, particularly with a benzoyl or nicotinoyl group (Figure 19) [15].

**Figure 23:**
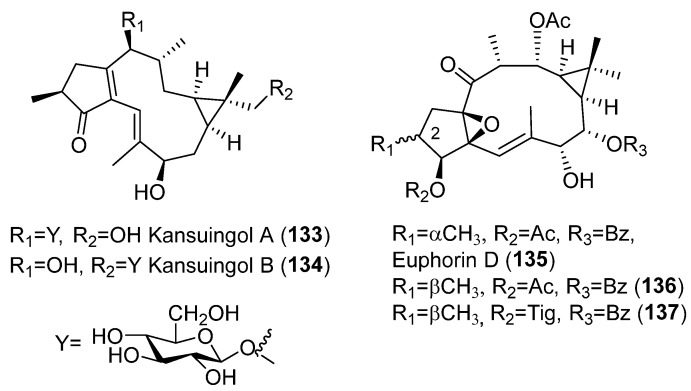
Structure of compounds **133**–**137**.

Lathyrane-type diterpene glycosides have demonstrated anti-inflammatory activity. Kansuingol A and B (**133**–**134**) (Figure 23), diterpenes isolated from the roots of *Euphorbia kansui*, were shown to be, at least in part, responsible for the anti-inflammatory effect shown by the butanol-soluble extract of the roots of this plant. This is because they potently inhibited the IL-6 production in HMC-1 cells stimulated by a combination of PMA and ionophore. Furthermore, **133** inhibited TNFα and IL6 mRNA expression level [67].

In a study for determining the anti-inflammatory potential of diterpenoids from *E. antiquorum*, three ingol-type diterpenoids (**135**–**137**) (Figure 23), ingol derivatives (Figure 19E), displayed a strong NO-inhibitory effect. Euphorin D (**135**) differs from **136** and **137** in the configuration of the methyl group at C-2 [68].

**Figure 24:**
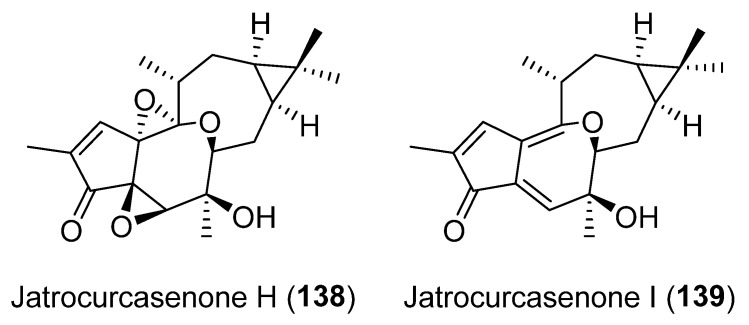
Structure of Jatrocurcasenones H and I.

Jatrocurcasenones H (**138**) and I (**139**) (Figure 24), two lathyrane diterpenoids containing an 7,14-oxygen bridged, were recently isolated from the roots of *Jatropha curcas L*., along with a number of lathyrane (jatrocurcasenones F and G and 4*Z* and 4E-jatrograrossidentation), which do not possess anti-inflammatory activity. Compounds **138** and **139** showed potent inhibitory activities against LPS-induced NO production in RAW264.7 cells, which suggests that the epoxy ring may play an important role in the activity [69].

#### Mode of Action

The ability to produce inflammatory cytokines, including IL-1β and IL-6, when cells were treated with EFL_30_ (**113**) (Figure 18) was evaluated; the results showed a significantly elevated production of them in the cell supernatant of LPS-induced RAW264.7. The investigation of the mechanism of modulation of pro-inflammatory cytokine response showed that **113** reduced the expression level of inducible nitric oxide synthase (iNOS) and NF-κB in a dose-dependent manner. In addition, compound **113** reduced the phosphorylation of IκBα and eliminates LPS-induced nuclear translocation of NF-κB. These results indicate that **113** exerts its anti-inflammatory activity by interfering with the phosphorylation of IκBα, thereby blocking the expression and nuclear translocation of NF-κB and reducing the expression of iNOS [16].

Similar results were obtained in the investigation of mode of action of EFL_2_ (**77**) (Figure 8), which showed robust inhibitory effects on the production of IL-1β and IL-6, tumor necrosis factor-α (TNF-α) and IL-8 released from LPS-stimulated RAW264.7 cells in vitro. Consistently, experiments in vivo showed that **77** exerted a potent anti-inflammatory effect by decreasing the levels of IL-1β and IL-6, TNF-α and IL-8 and myeloperoxidase (MPO) in the lung and bronchioalveolar lavage fluid. EFL_2_ (**77**) inhibition appeared to be mediated by NF-κB signaling activation, but not the MAPK pathway. Additionally, **77** decreased phosphorylation of IKK α/β and IκBα levels, and significantly suppressed p65 translocation and its DNA-binding activity [56]. In the same way, euplarisan A (**126**) (Figure 21) inhibited the generation of inflammatory cytokines, such as IL-1β and IL-6 and TNF-α. Additionally, **126** decreased the expression of the crucial proteins of inflammatory signaling pathway oxide synthase (iNOS), cyclooxygenase-2 (COX-2) and IκBα, further blocking the expression of NF-κB and nuclear translocation [19].

To explore the possible mode of NO inhibition, the binding interaction of compounds **135**–**137** (Figure 23) with iNOS and COX-2 were investigated through molecular docking studies, which revealed that the three compounds had strong interactions with the protein. Compound **136** was selected to test on iNOS/COX-2 protein expression. Treatment of LPS-stimulated BV2-2 cells with **136** produced significant decrease of iNOS and COX-2 levels, indicating that these compounds may exert their anti-inflammatory effects by down-regulation of iNOS and COX-2 protein levels [68]. The same effects were observed in the study of mode of action of compounds **138** and **139** (Figure 24), where a further differential gene expression (DGE) analysis was conducted to investigate the underlying genes targeted by jatrocurcasenone I (**139**) in LPS-induced RAW264.7 macrophages [69]. The results demonstrated that **139** has a regulatory effect on the 587 DEGs, mainly related to immune diseases, immune systems, signaling molecules and interaction and signal transduction. 24 of those DEGs were associated with the inflammatory responses, including: interkeukin and interleukin-related genes (*IL1α*, *1L1β*, *IL1f6*, *IL*-*1rn* and *IL*-*27*); chemokines (*Ccl*2, *Ccl*5, *Ccl*7, *Ccl*9, *Ccl*22 and CXcl10); intracellular signaling (*Trim*25; *Bcl*2*a*1*a*, *Dusp*1, *Dusp*2, *Ptgs*2 and *End*1); and transcription factor (*Nr*4*a*1). The molecular mechanism underlying the protection of RAW264.7 cells from inflammation could be due to the regulation of these genes.

### 3.4. Antiviral and HIV-1 Reactivation Activities

**Figure 25:**
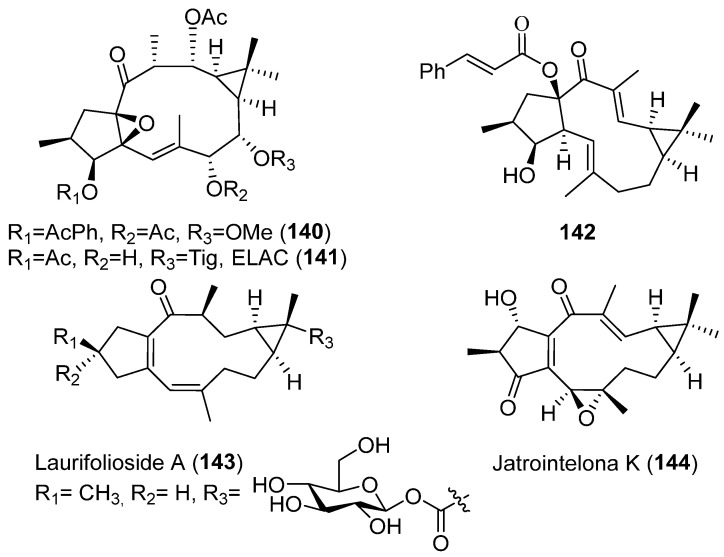
Structure of lathyranes with antiviral or HIV-1 reactivation activity.

The activity of three highly functionalized ingol-type diterpenes, characterized for the presence of a phenylacetate group between their substituents, was tested on HIV-LTR transactivation by measuring the levels of GFP as a marker of HIV promoter activation. Only one of them, 8-methoxyingol 7,12-diacetate-3-phenylacetate (**140**) (Figure 25) induced cell-cycle arrest in Jukart-LTR-GFP cells and HIV-1-LTR promoter activation [70]. Other ingol-type diterpene, 3,12-di-O-acetyl-8-*O*-tigloylingol (ELAC, **141**) (Figure 25), also showed the ability to reactivate HIV-1 latency in a concentration-dependent manner [71]. The study of its mechanism of action revealed that this activity could be mediated by PKC activation. **141** induced IκBα phosphorylation and its subsequent degradation suggesting that the activation can be induced by NF-κB. Furthermore, probably other transcription factors can probably contribute to the reactivation of HIV-1 from latency, as JNK and ERK were also phosphorylated [71].

On the other hand, some lathyrane diterpenoids have shown activity against HIV-1 replication. In the study on the activity shown by the diterpenes isolated from the roots of *E. Micractina*, 15-cinnamoyloxy-3-hydroxylathyra-5,12-dien-14-one (**142**) (Figure 25) exhibited weak activity, with an IC50 value of 8.2 μM, while the positive control zidovudine gave 0.05 μM [72]. With these precedents and encouraged by the activity displayed by related diterpenes [73], the anti-HIV activity of ethanolic extracts from *Euphorbia lathyris* seeds of different origins was evaluated [74]. Although the results showed a significant activity of all the tested extracts, the isolated diterpenoids, most of them lathyrane types, were inactive against HIV viral replication, indicating a possible synergetic effect [74].

Activity of this kind of diterpenes against other viruses has also been investigated. For example, in the phytochemical study of the buds of *Wikstroemia chamaedaphne* Meisn, it was observed that only laurifolioside A (**143**) (Figure 25), one of the six lathyrane diterpenes isolated, exhibited potential anti-hepatitis B virus activity [75].

In an investigation to determine the antifeedant and antiviral activity of diterpenoids from the fresh roots of *E. jolkinii* resulted in jolkinol A (**89**) (Figure 12) showing significant anti-respiratory syncytial virus (RSV) activity [76].

The ethyl acetate extract of the trunk bark of *Sandwithia guyanensis* showed a strong anti-chikungunya virus (CHIKV) activity. The study of the most active fractions led to the identification of 19 diterpenoids with different carbon skeletons. Only one of them, possessing a lathyrane framework and was named jatrointelone K (**144**) (Figure 25), showed a moderate anti-CHIKV activity [77].

### 3.5. Neurogenesis Promoting

**Figure 26:**
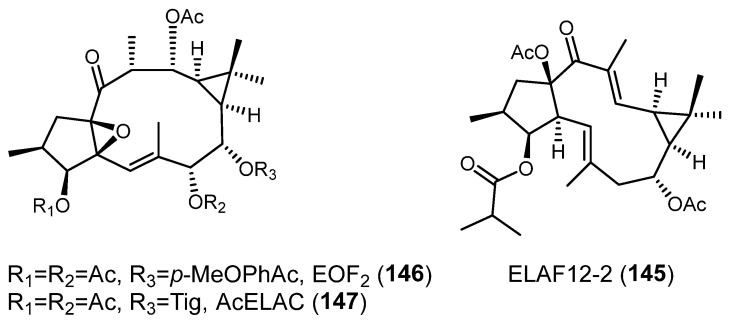
Structure of lathyrane tested on neurogenesis promotion activity.

Three studies have been carried out on the neurogenesis promotion activity of lathyrane-type diterpenes. In the first one, four lathyranes, **140**, ELAC (**141**) (Figure 25) and **145**–**146** (Figure 26) were tested [5]. The culture in a bFGF-supplement medium with the different lathyranes produces a significant increase in neurosphere size when cultured with **141**, without modifying neurosphere size. No effect was observed when treated with **145**, **146** or the acetylated derivative of **141** on C-7-OH (AcELAC, **147**) (Figure 26). Studies on mechanism of action showed that classical PKCs, especially PKCβ, are responsible for ELAC-dependent NPC proliferation. The lathyrane ELAC (**141**) also stimulated NPC proliferation in vivo [5].

In a second study, the effects of epoxyboetirane A (**11**) (Figure 4) and euphoboetirane A (**54**) (Figure 6) on NPC proliferation was evaluated. Epoxyboetirane A (**11**) resulted in an increase in the size of neurospheres in cultures stimulated with a combination of the growth factors EGF and bFGF, while no statistically significant effect was observed in cultures stimulated with either EGF or bFGF alone. Euphoboetirane A (**54**) induced a smaller increase in the neurosphere size and in the presence of both growth factors. On the other hand, the capacity to form neurospheres was not affected by any of the two compounds, since no changes were found in their number [78].

Finally, the capacity to activate PKC, to facilitate neuregulin 1 release and to promote neuroblast differentiation and survival in cultures of subventricular zone of lathyrane diterpene EOF2 (**146**) (Figure 26) was proved. Local infusion of **146** in mechanical cortical injuries induced neuroblast enrichment within the perilesional area and, when it was administered intranasally, promoted migration of neuroblasts from the subventicular zone toward the injury. The results show that the neural differentiation of NPC in neuroblasts promoted by **146** was mediated by novel PKCs, especially PKCϴ [4].

The comparison of the structures of **140**, **141** and **145**–**147** demonstrates the important role of the substituents on C-3-OH, C-7-OH and C-8-OH in the interaction with the PKCs of this class of diterpenes. Further studies are needed to determine the essential structural elements in each type of activity and how these influence the interaction with PKCs.

### 3.6. Others

#### 3.6.1. Anticholestasis

**Figure 27:**
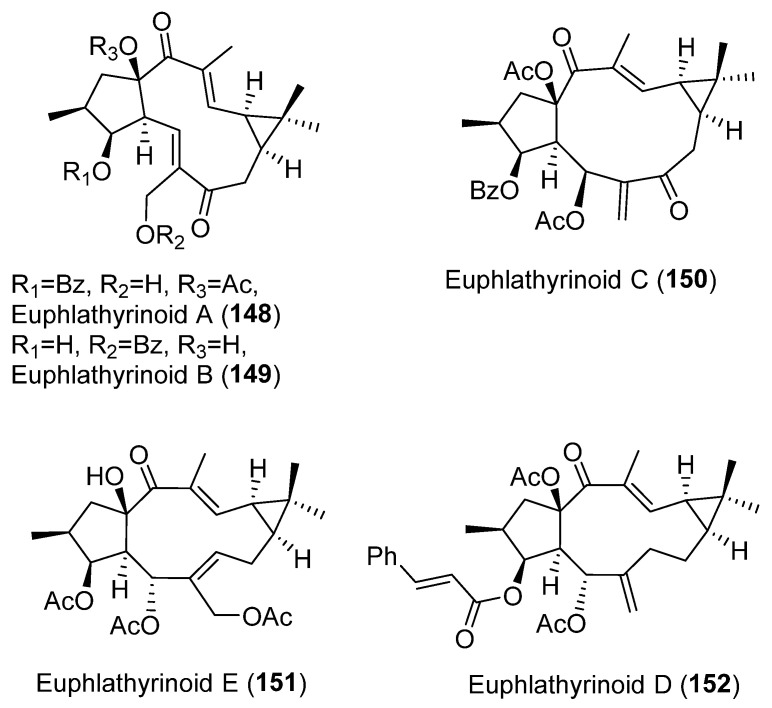
Compounds with anticholestasis activity.

Recent studies have suggested that lathyrane diterpenoids could serve as a new type of human pregnane X receptor (hPXR) agonist for future anticholestasis drug development. In a bioassay-guided isolation on *E. lathyris* extract looking for an hPXR agonistic compound, 16 lathyrane diterpenoids were isolated [79]. Five of them were novel compounds and were named euphlathyrinoid A–E (**148**–**152**) (Figure 27). Known compounds EFL_1–2_ (**76, 77**) (Figure 8), EFL_3_ (**43**) (Figure 6), EFL_8–9_ (**91**, **80**) (Figure 8 and Figure 13), EFL_11_ (**116**) (Figure 18), EFL_30_ (**113**) (Figure 18) and EFL_31_ (**121**) (Figure 20) were identified. The major components, EFL_2_ (**77**) and EFL_3_ (**43**), were subjected to chemical transformations, generating a lathyrane library containing 34 compounds that were used for a systematic hPXR agonistic activity investigation. The main structural modification included the Δ^6,17^ terminal double bond, the α,β-unsaturated ketone, the cyclopropane ring, as well as the substituents on C-3, C-5, C-7, and C-15. Three natural products, euphlathyrinoid C (**150**), EFL_30_ (**113**) and EFL_31_ (**121**), and a synthetic compound, were excluded from the hPXR agonistic screening because of their cytotoxicity on HEF293T cells. The remaining compounds were subjected to the hPXR agonistic screening, most of them showing a potent activity at the concentration of 10 μM. The most active of them were subjected to further assays, showing a dose–response-dependent activity. EFL_9_ (**80**) (Figure 8), was found to be the most active compound, this could significantly activate hPXR, as evidenced by the hPXR reporter gene activity (6.9-fold), and up-regulate the expressions of hPXR downstream key genes CYP3A4, CYP2B6, and MDR1. SAR studies revealed that acyloxy substituents on C-7, specifically a nicotinoyl group, and the presence of 14-carbonyl were essential to activity [79].

#### 3.6.2. Antibacterial

**Figure 28:**
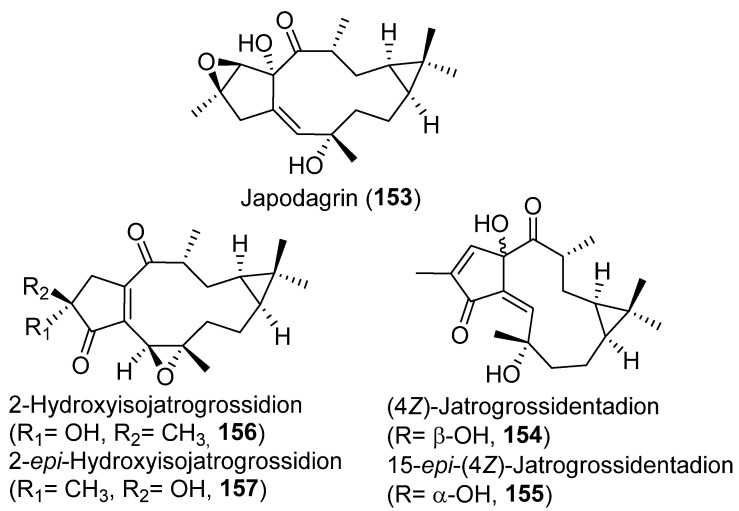
Structure of diterpenes isolated from the root of *J. podagrica* Hook.

Japodagrin (**153**) (Figure 28) is a compound with an unusual epoxy function in ring A of the lathyrane skeleton. It was isolated from the root of *Jatropha podagrica* Hook [80], a plant that had shown a range of biological activities, including antibacterial. Its antibacterial activity and that of four other known diterpenoids, 4Z-jatrogrossidentadion (**154**), 15-epi-4Z-jatrogrossidentadion (**155**), 2-hydroxyisojatrogrossidion (**156**), and 2-*epi*-hydroxyisojatrogrossidion (**157**) (Figure 28), also isolated from this plant, were tested against *Bacillus subtilis* (ATCC 6051), *Staphylococcus aureus* (ATCC 25923) *Escherichia coli* (ATCC 25922) and *Pseudomonas aeruginosa* (ATCC 27853). All of which resulted in different levels of microbial killing against gram-negative bacteria (*B. subtillus* and *S. aureus*), but were found to be inactive in disk assays against the gram-positive ones (*E.coli* and *P. aeruginosa*) [80]. All compounds were tested in standard disk assays, at 20 μg/disk doses, with streptomycin and gentamycin as positive controls. The most active compound was 2-*epi*-hydroxyisojatrogrossidion (**157**), which gave the same inhibition against *B. subtillus* and *S. aureus* than streptomycin (35 and 26 mm inhibition zones, respectively), and similar to the inhibition exerted by gentamycin against *B. subtillus* and *S. aureus* (34 and 28 mm, respectively). Compounds **156**, **155**, **154** and **153** were less active, with respective inhibition zones of 31, 17, 20 and 16 mm against *B. subtillus* and of 21, 9, 10 and 12 mm versus *S. aureus*.

#### 3.6.3. Vascular-Relaxing Activity

**Figure 29:**
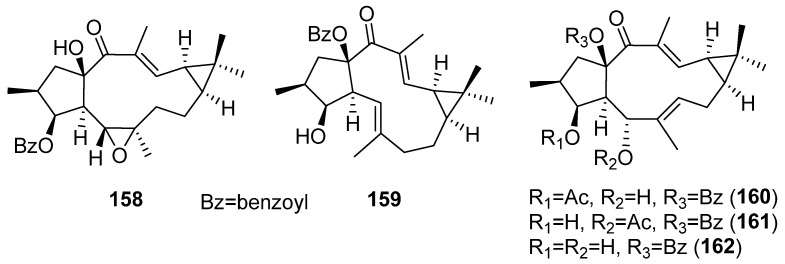
Lathyrane diterpenoids from *E. micractina* with vascular-relaxing activity.

Remarkably, vasodilatory activity was observed using a phenylephrine-induced vasoconstriction model with lathyrane diterpenoids isolated from *E. micractina* [72]. Vascular relaxation was found in compounds containing a benzyloxy group, primarily at C-15, (**99**) (Figure 15) and **158**–**162** (Figure 29), indicating an important role of this group in vascular-relaxing activity.

#### 3.6.4. Gastrointestinal Toxicity

*E. lathyris* L. is a traditional Chinese medicine. It produces several side effects, including irritation of the gastrointestinal tract, which manifests as severe diarrhea [81]. The studies carried out to identify the components responsible for this effect indicated that the diterpene EFL1 (**76**) (Figure 8) could be the main cause of the diarrhea [82]. The intestinal toxicity of **76** and the underlying mechanisms were studied using nematode *Caenorhabditis elegans* as the model. The results show that toxicity was related to intestinal oxidative damage, disorder transportation, down-regulated cell junctions, enhanced rhythm behavior, muscle contraction and injured GABAergic neurons [83]. The gastric cytotoxicity of **76** and the underlying mechanism in human gastric mucosa epithelium cells was also investigated. EFL1 (**76**) induced oxidative stress, activation of mitochondrial-mediated apoptosis in GES-1 cells and authophagy via inhibition of the PI3K/AKT/mTOR pathway [84].

#### 3.6.5. Osteoclastogenesis Inhibition

In another study carried out on the biological activity of the diterpene EFL_1_ (**76**) (Figure 8), compound **76** was proposed as a potential therapeutic agent to prevent or treat bone-related diseases caused by an excess of osteoclast, since it inhibited osteoclast differentiation by regulating cellular redox status and induced Fas-mediated apoptosis in osteoclast [85].

#### 3.6.6. Inhibition of 11β-HDS1

**Figure 30:**
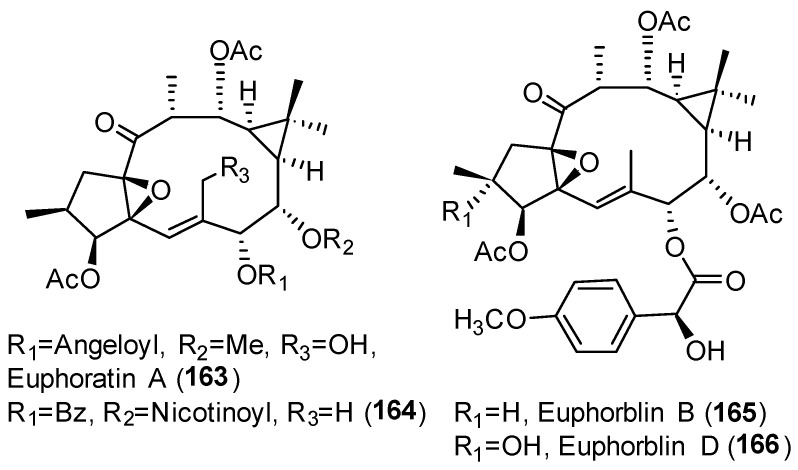
Ingol-type diterpenoids with the abilities to inhibit mouse 11β-HDS1 (**163**–**164**) and to induce lysosomal biosynthesis (**165**–**166**).

11β-Hydroxysteroid dehydrogenase Type 1 (11β-HDS1) is an attractive therapeutic target for the treatment of a number of diseases such as obesity and metabolic and cardiovascular disease [86]. Ingol type diterpenes were investigated for an inhibitory effect on human and mouse 11β-HDS1 from a set of compounds isolated from *Euphorbia antiquorum* [87]. Unfortunately, none showed inhibitory effects on human 11β-HDS1 and only three, euphorantins A (**163**) (Figure 30) and N (**81**) (Figure 8) and 3,12-diacetyl-7-benzoyl-8-nicotinylingol (**164**) (Figure 30), inhibited mouse 11β-HDS1. The cytotoxicity of these compounds against HL-60 (human premyelocytic leukemia) and A-549 (human lung adenocarcinoma) cells was also evaluated, as well as their inhibitory ability against the PTP1B enzyme, but none of them were active [87].

#### 3.6.7. Induction of Lysosomal Biosynthesis

A set of ingol-type diterpenoids, isolated from *E. resinifera*, named euphorblins A–Q and EFRL_4_ (EOF2, **146**) (Figure 26) were evaluated as inducing agents of lysosomal biosynthesis. Between them, EFRL_4_ (**146**), euphorblin B (**165**) and D (**166**) (Figure 30) proved to be promising compounds for the development of drugs for the treatment of lysosome-related diseases, as they showed high capacities to induce lysosome biosynthesis at different doses and concentrations [88].

#### 3.6.8. PGE2 Inhibition

*E. nivulia*, a succulent Euphorbiaceae found in the tropics, is known for its therapeutic properties against diseases like bronchitis and rheumatism [81]. The phytochemical study of the latex of this plant afforded five ingol-type diterpenes which were tested for the lipo-polysaccharide (LPS)-induced PGE_2_ inhibition activity. Only that with a free hydroxyl group on C-3, 7-angeloyl-12-acetyl-8-methoxyingol (**102**) (Figure 17) showed significant PGE_2_ inhibition, the IC_50_ value (0.003 µM) being less than that of the reference compound celecoxib (0.050 μM) [89].

## 4. Drug Delivery

As discussed above, lathyranes are a class of diterpenes which have a range of intriguing biological activities that are ripe for exploitation, yet, in striking contrast with other well developed diterpenoids such as taxanes, very few have made it into the clinic. This may be due to the side effect profiles of the drugs, the complexity of formulating them into dosage forms that are compatible with a range of routes for drug delivery, as well as the $1400 million [90] cost of bringing a small molecule drug to market. That said, the potential of these compounds in the treatment of cancer and neurological disease suggests a significantly untouched resource in commercially rich fields of clinical treatment.

The oral route for delivery of pharmaceuticals is the most widely used and accepted [91]. In order to get an idea of the druglikeness of lathyranes, Lipinski’s rule of 5 [20], originally based on physicochemical profiles of phase 2 drugs, can be applied. This rule (of thumb) predicts that poor drug absorption or permeation for a molecule across the gastrointestinal tract is less likely if there are more than 5 hydrogen bond donors, 10 hydrogen bond acceptors, the molecular weight is greater than 500 and the calculated Log P (ClogP) is greater than 5. This clearly indicates that for oral drug delivery, many diterpenoid lathyranes have good druglikeness, as they are relatively small and lipophilic. However, the rule does not take into account gut anatomy and physiology, which includes, for example, the coverage of the gastrointestinal wall lining with a barrier of mucous that has to be diffused through, or the presence of active transporters, as well as the efficiency, first pass metabolism in the liver, all of which directly affect the pharmacokinetic profile of the drug. This led to a preference for clinically used diterpenoids, such as the anticancer drugs Paclitaxel and Docetaxel, to be formulated for the intravenous route. However, new approaches are being considered to avoid the invasive nature of the intravenous route, for example, currently in clinical trial is a novel oral formulation of the anticancer drug, Docetaxel. It is co-administered with cytochrome P450 3A4 and the P-gp inhibitor ritonavir; this strategy has demonstrated increased oral bioavailability [92].

Diterpenoid formulation to a medicinal product can be complex and lengthy. Paclitaxel was first isolated from the Pacific Yew (*Taxus brevifolia*) in 1971, yet was not approved for medical use until 1993. Originally solubilized in 75% polyethylene glycol, it was found that activity of Paclitaxel was significantly reduced. This was resolved using cremophor EL, a polyethoxylated caster oil as a non-ionic solubilizer and emulsifier. In aqueous solution and by implication in serum, Cremophor EL forms star shaped micelles. These macromolecular structures have been problematic in triggering a part of the human innate immune system called the Complement system [93]. Activation of the complement system causes hypersensitivity reactions, which at worst can result in patient death [94]. Mechanistically, it is the excipient cremophor EL and not the drug that causes the clinically relevant toxicity. Mechanistically, these side effects are driven by portions of the cremophor EL’s hydrophobic components incorporating into lipoproteins [95,96]. This results in loss of hydrophobic character in the micelles, resulting in the formation of droplets of 100–300 nm in size and it is these that are believed to trigger the complement system. These types of reactions have brought focus onto the immunological interactions of nano-carrier vehicles with the immune system [97]. This research is enabling safer design and has produced a range of carriers with far greater performance in terms of stealth characteristics for avoidance of the immune system, thus providing safer intravenous systems for potential inclusion of lathyranes. These include a wide range of formulations, including pegylated liposomes [98], cubosomes [99], dendrimers [100], albumin nanoparticles [101], as well as complement inhibitors [102].

Recently, the phytochemical lathyrane ELAC (**141**) (Figure 25) has been found to promote endogenous neurogenesis in adult CD1 mouse brains [5]. As the active pharmacophores do not fit into the ‘rule of 5′ in terms of excess hydrogen bond donors, it is unlikely that it is absorbed across the gastrointestinal tract readily. Also, its lipophilicity is such that it is not high enough to diffuse across the barrier, and structurally would also be potentially prone to the effects of efflux pumps such as P-gp. A number of strategies have recently been developed that may facilitate the uptake of lathyranes, most likely using the intravenous route for drug delivery to the brain via the blood brain barrier (BBB). For example, self-assembled nanoligand drug carriers based on phage display peptide target cerebral endothelial cells through transferrin receptor-reaching neurons and microglial cells [100], without damage to the BBB. Significantly, more invasive methods have been developed to open the BBB in situ to drugs. This includes techniques such as magnetic resonance-guided focused ultrasound. This technique transiently permeabilises the BBB and was applied in a recent human trial for opening in eloquent primary motor cortex [103]. Another intriguing method for drug delivery to the brain has been reported where transient breakdown of the BBB can be achieved via modulation of the UNC5B receptor [104] that controls BBB integrity. If the time frame of the opening of the brain endothelial cells can be effectively controlled, this may facilitate a less invasive method for opening the BBB to small molecules such as lanthyranes. An alternative route is the intra-nasal, where the drug non-invasively bypasses the BBB following transport along the olfactory and trigeminal nerves [105]. The primary process of drug absorption is through the mucus and is ideal for lipophilic and low molecular weight drugs (<400 Da), even with poor stability in fluids [106]. Significant advances are being made in this area of drug delivery, which seems well suited to the diversity of centrally acting lathyranes and may offer a much safer route that does not affect the homeostatic condition of the brain.

Clearly, there is significant potential for lathyranes to be used orally. The problem is that this results in the non-site-specific delivery of the drug to the whole body. Nanocarriers have the advantage in the case of current solid tumour treatment of site-specific targeting. This reduces the side-effect profile of the drug, such as cardiotoxicity associated with administration of free doxorubicin. Therefore, nano-carriers offer an excellent targeting system for administration of lathyranes with anti-cancer activity in the future.

## 5. Conclusions

This review demonstrates the importance of lathyranes as privileged structures in drug design. The ability of the lathyrane framework to direct the functional groups that decorate its skeleton in a well-defined space makes the interaction of these compounds with various targets possible, turning them in potential therapeutic agents to prevent or treat different types of diseases, even though no clinical applications have yet been described.

Modulation of multidrug resistance (MDR) has enough studies to draw some generalized structure/activity correlations. Four groups of lathyrane derivatives have been shown to promote reversal of MDR (Figure 2), where functionalization patterns are relevant. For 5,6-epoxy-lathyranes (see **A** in Figure 2), presence of aromatic moieties at C-15 and C-16, as well as free hydroxyl groups at C-3 and C-20 is required. On the other hand, active 6,17-epoxy lathyranes (see **B** in Figure 2) and Δ^6,17^ double bond lathyranes (see **D** in Figure 2) bear aromatic moieties at C-5 and C-20. Presence of an aromatic moiety at C-3 also plays a key role in activity in Δ^5,6^ double bond lathyranes (see **C** in Figure 2). The mechanism of action for most of the above-mentioned compounds seems to involve inhibition of P-gp drug efflux pump, but it is not the only one involved, as shown by jolkinol B (**83**).

For cytotoxic activity, less clear-cut tendencies can be drawn. 5,6-epoxylathyranes Figure 11A) with ester groups at C3, C-15 and C-16 have shown activity against multidrug-resistant EPG85-257RDB cells, through apoptosis induction. On the other hand, Δ^6,17^ double bond lathyrane derivatives (Figure 11D) with aromatic moieties at C-3 and C-7 and acetate groups at C-5 and C-15 have been described as active against KB-VIN cells. For these compounds, two different modes of action seem to be present. EFL_3_ (**43**) and EFL_9_ (**80**) acted disrupting normal cell cycle progression, whereas EFL_2_ (**77**) and EFL_8_ (**91**) induced both actin filament aggregation, as well as partial disruption of microtubule networks.

Several structural variants of the lathyrane skeleton have been evaluated for anti-inflammatory activity, inhibition of NO production out of mouse macrophages in response to bacterial lipopolysaccharide (LPS) being the standard test. 6,7-Epoxy-lathyranes (Figure 19B) with aromatic moieties at C-3 and C-5 induce this effect, as well as Δ^5,6^ double bond lathyrane derivatives (Figure 19C) with a wide variety of substituents. Compounds with a Δ^6,17^ double bond (19D) have also proved to be active, such as EFL_30_ (**113**). Regulation of iNOS and COX-2 protein levels seems to be involved in the mode of action of the above compounds, as well as in ingol derivative **136** (Figure 19E).

Further biological activities, such as antiviral, antibacterial, vascular-relaxing, gastrointestinal toxicity, osteoclastogenesis inhibition, inhibition of 11β-HDS1, induction of lysosomal activity and PGE2 inhibition have been described, with very few results to draw general conclusions. On the other hand, neurogenesis-promoting experiments have shown that ingol derivatives with a free hydroxyl group at C-7, such as ELAC (**141**), promote neural progenitor cell (NPC) proliferation in a classical PKC-dependent manner. Finally, anticholestasis evaluation of lathyrane derivatives, through agonist interaction with the human pregnane X receptor (hPXR), shows that Δ^6,17^ double bond lathyrane derivatives were active as long as they bear acyloxy groups at C-7 and a carbonyl group at C-14 is present.

Currently, a preference for intravenous administration is observed in clinically used diterpenoids. However, significant advances are being made in the area of drug delivery, which may offer a much safer and non-invasive route for administration of lathyrane-type diterpenes.

## 6. Forward-Looking Outlook and Recommendations

The current trend for publications (Web of Science) on the subject of lathyranes is increasing. Continuation of this upward trend will reveal new and as of yet unexpected biological activity. For example, induction of neurogenesis for potential application in brain disorders. Much of the current work is focused on inhibition of efflux pumps and it is expected that these molecules are very likely to have other biological activities as well. A combination of drug efflux pump inhibition and cytotoxic activity could prove a viable clinical tool against chemotherapeutically resistant brain tumours such as glioma. Alternatively, one can readily envisage a delivery system having two molecules to bring about efflux inhibition and the other to invoke cytotoxicity.

The lack of lathyranes in clinical use, in spite of extraordinary potential, suggests that there is a significant rate-limiting step to the clinic. In part, this may be due to toxicity; however, in the case of those molecules that have acceptable profiles for use in humans, formulation to a medicinal form is problematic. It would therefore be worthwhile to apply the nanotechnology that has made paclitaxel so effective in treating metastatic breast cancer and HIV Karposi sarcoma. Although the majority of pharmaceutical companies may not have used carriers such as liposomes, micelles and albumin, application of this technology to the delivery of lathyranes may circumvent problems of solubility that was inherent in the 30-year time frame to formulate paclitaxel. Embracing the nano approach may now be economically viable because of the unique biological activity inherent in many of the lathyrane diterpenes and open up the potential of these compounds for medicinal use.

## Data Availability

No new data were created or analyzed in this study. Data sharing is not applicable to this article.

## Supplementary Materials

The following supporting information can be downloaded at: https://www.mdpi.com/article/10.3390/ph15070780/s1: Section S1: Bioactive lathyrol derivatives; Table S1: Bioactive lathyrol derivatives: biological activities, observed effects and molecular targets; Section S2: Bioactive 7-hydroxylathyrol and 7-oxylathyrol derivatives; Table S2: Bioactive 7-hydroxylathyrol and 7-oxolathyrol derivatives: biological activities, observed effects and molecular targets; Section S3: Bioactive 12-hydroxylathyrol derivatives; Table S3: Bioactive 12-hydroxylathyrol derivatives: biological activities, observed effects and molecular targets; Section S4: Bioactive 12,15-epoxylathyrol derivatives; Table S4: Bioactive 12,15-epoxylathyrol derivatives: biological activities, observed effects and molecular targets; Section S5: Bioactive 6,17-epoxylathyrol derivatives; Table S5: Bioactive 6,17-epoxylathyrol derivatives: biological activities, observed effects and molecular targets; Section S6: Bioactive isolathyrol derivatives; Table S6: Bioactive isolathyrol derivatives: biological activities, observed effects and molecular targets; Section S7: Bioactive jolkinol derivatives; Table S7: Bioactive jolkinol derivatives: biological activities, observed effects and molecular targets; Section S8: Bioactive 15-deacyljolkinol B derivatives; Table S8: Bioactive 15-deacyljolkinol B derivatives: biological activities, observed effects and molecular targets; Section S9: Bioactive laurifolioside derivatives; Table S9: Bioactive laurifolioside derivatives: biological activities, observed effects and molecular targets; Section S10: Bioactive jatrogrossidion derivatives; Table S10: Bioactive jatrogrossidion derivatives: biological activities, observed effects and molecular targets; Section S11: Bioactive ingol derivatives; Table S11: Bioactive ingol derivatives: biological activities, observed effects and molecular targets. References [107,108,109,110,111,112,113,114,115,116,117,118,119,120,121,122,123,124,125,126,127] are cited in the supplementary materials.
