# Supplementary material for: Pharmacological Potential of Lathyrane-Type Diterpenoids from Phytochemical Sources
Source: Pharmaceuticals (Basel). 2022 Jun 23;15(7):780. doi: 10.3390/ph15070780 (PMC9318715; doi:10.3390/ph15070780)
Supplement: Supplementary file 1 [file pharmaceuticals-15-00780-s001.zip › pharmaceuticals-1745823-supplementary.pdf]

Supplementary Material

Pharmacological potential of lathyrane-type diterpenoids from phytochemical sources

Fátima Vela,[ɸ] Abdellah Ezzanad,[ɸ] Antonio J. Macías-Sánchez[ɸ], A. Christy Hunter[¥], Rosario Hernández-Galán[ɸ],*

[ɸ] Departamento de Química Orgánica and Instituto de Investigación en Biomoléculas (INBIO), Facultad de Ciencias, Universidad de Cádiz, 11510 Puerto Real, Cádiz (Spain).
[¥] School of Pharmacy, College of Science, University of Lincoln, Lincoln, United Kingdom

Table of contents

Section S1. Bioactive lathyrol (**53**) derivatives..**S2**

Table S1. Bioactive lathyrol (**53**) derivatives: biological activities, observed effects and molecular targets (where available)..**S5**

Section S2. Bioactive 7-hydroxylathyrol (**92**) and 7-oxolathyrol derivatives..**S10**

Table S2. Bioactive 7-hydroxylathyrol (**92**) and 7-oxolathyrol derivatives: biological activities, observed effects and molecular targets (where available)..**S11**

Section S3. Bioactive 12-hydroxylathyrol derivatives..**S14**

Table S3. Bioactive 12-hydroxylathyrol derivatives: biological activities, observed effects and molecular targets (where available)..**S15**

Section S4. Bioactive 12,15-epoxylathyrol derivatives..**S16**

Table S4. Bioactive 12,15-epoxylathyrol derivatives: biological activities, observed effects and molecular targets (where available)..**S17**

Section S5. Bioactive 6,17-epoxylathyrol (epoxylathyrol (**10**)) derivatives..**S19**

Table S5. Bioactive 6,17-epoxylathyrol (epoxylathyrol (**10**)) derivatives: biological activities, observed effects and molecular targets (where available)..**S21**

Section S6. Bioactive isolathyrol (**S47**) derivatives..**S28**

Table S6. Bioactive isolathyrol (**S47**) derivatives: biological activities, observed effects and molecular targets (where available)..**S29**

Section S7. Bioactive jolkinol (**S62**) derivatives..**S31**

Table S7. Bioactive jolkinol (**S62**) derivatives: biological activities, observed effects and molecular targets (where available)..**S35**

Section S8. Bioactive 15-deacyl jolkinol B (**S68**) derivatives..**S40**

Table S8. Bioactive 15-deacyl jolkinol B (**S68**) derivatives: biological activities, observed effects and molecular targets (where available)..**S42**

Section S9. Bioactive laurifolioside (**105**) derivatives..**S46**

Table S9. Bioactive laurifolioside (**105**) derivatives: biological activities, observed effects and molecular targets (where available)..**S47**

Section S10. Bioactive jatrogrossidion (**S78**) derivatives..**S48**

Table S10. Bioactive jatrogrossidion (**S78**) derivatives: biological activities, observed effects and molecular targets (where available)..**S49**

Section S11. Bioactive ingol (**S87**) derivatives..**S52**

Table S11. Bioactive ingol (**S87**) derivatives: biological activities, observed effects and molecular targets (where available)..**S55**

Section S1. Bioactive lathyrol (**53**) derivatives.

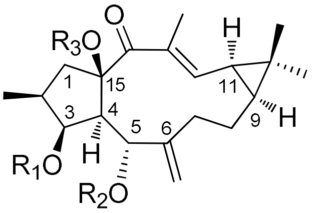

43, 53-54, 91, 107, 114, 122, 130, 153, S1

Compound	R_1	R_2	R_3	CAS RN	Common name
**43**	Bz	Ac	Ac	218916-52-0	Euphorbia factor L_3
**53**	H	H	H	34420-19-4	Lathyrol
**54**	Ac	Ac	Ac	1613699-95-8	Euphorbia factor L_{15}
**91**	Nic	Ac	Ac	218916-53-1	Euphorbia factor L_8
**107**	PhAc	Ac	Ac	247099-01-0	Deoxy-Euphorbia factor L_1
**113**	Cin	H	Ac	2293060-22-5	Euphorbia factor L_{30}
**121**	H	Cin	Ac	2293060-23-6	Euphorbia factor L_{31}
**129**	Bz	H	Ac	-	Euphorbia factor L_{32}
**152**	Cin	Ac	Ac	2750897-06-2	Euphlathyrinoid D
**S1**	Salicyloyl	Ac	Ac	1613699-97-0	Euphorbia factor L_{16}

Ac = acetyl; Bz = benzoyl; Cin = cinammoyl; PhAc = phenylacetyl; Nic = nicotinoyl.

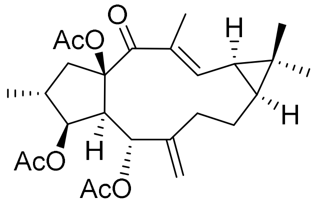

2-*epi*-Lathyrol triacetate (**S2**)
(CAS RN: 489459-81-6)

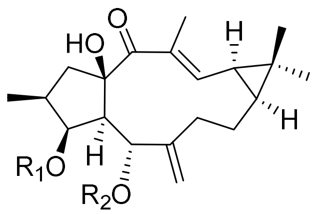

44-50, 133, S3-S20

Compound	R1	R2	CAS RN	Compound	R1	R2	CAS RN
**44**	H	PhAc	1708993-46-7	**46**	Bz	Bz	1708993-56-9
**45**	H	NpAc	1708993-49-0	**47**	Pr	Bz	1708993-61-6
**S3**	H	Ac	1708993-38-7	**48**	Pr	PhAc	1708993-62-7
**S4**	H	Prop	1708993-39-8	**49**	Pr	NpAc	1708993-63-8
**S5**	H	*n*-Val	1708993-40-1	**50**	Cin	Bz	1708993-65-0
**S6**	H	*n*-Hex	1708993-41-2	**132**	Cin	Cin	2473033-45-1
**S7**	H	*i*-Bu	1708993-42-3	**S13**	Ac	Ac	35556-35-5
**S8**	H	*i*-Val	1708993-43-4	**S14**	Pr	Pr	1708993-52-5
**S9**	H	Nic	1708993-44-5	**S15**	*n*-Bu	*n*-Bu	1708993-53-6
**S10**	H	Bz	1708993-45-6	**S16**	*n*-Hex	*n*-Hex	1708993-55-8
**S11**	H	PhPr	1708993-47-8	**S17**	PhAc	PhAc	1708993-58-1
**S12**	H	Cin	1708993-48-9	**S18**	PhPr	PhPr	1708993-59-2
				**S19**	NpAc	NpAc	1708993-60-5
				**S20**	Bz	NpAc	1708993-64-9

Ac = acetyl; *i*-Bu = isobutyroyl; *n*-Bu = *n*-butyroyl; Bz = benzoyl; Cin = cinammoyl; *n*-Hex = hexanoyl; Nic = nicotinoyl; NpAc = 1-naphthylacetyl; PhAc = phenylacetyl; PhPr = 3-phenylpropionyl; Pr = propionyl; *i*-Val = 3-methylbutanoyl; *n*-Val = *n*-valeryl.

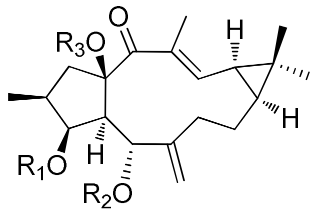

55-65, 68

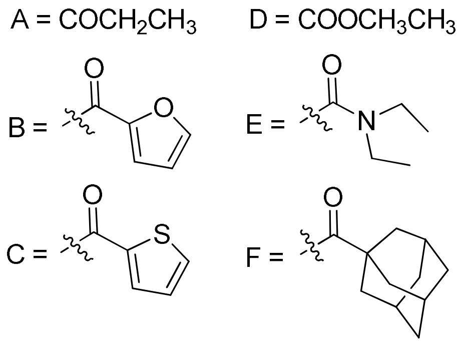

Compound	R_1	R_2	R_3	CAS RN	Common name
**55**	A	Ac	Ac	-	Euphoboetirane B
**56**	B	B	H	-	Euphoboetirane C
**57**	C	C	H	-	Euphoboetirane D
**58**	H	Naph	H	-	Euphoboetirane E
**59**	A	A	H	-	Euphoboetirane F
**60**	H	E	H		Euphoboetirane G
**61**	H	H	D		Euphoboetirane H
**62**	H	F	H		Euphoboetirane I
**63**	H	Bz	H	1708993-45-6	Euphoboetirane J
**64**	H	*p*-CF_3Bz	H	2101545-30-4	Euphoboetirane K
**65**	H	*m*-MeOBz	H	2101545-32-6	Euphoboetirane L
**68**	H	Cin	H	1708993-48-9	Euphoboetirane O
**78**	Cin	Ac	Ac	-	-
**79**	Bz	Nic	Ac		

Ac = acetyl; Bz = benzoyl; Cin = cinnamoyl; Naph= 2-naphthoyl., Nic= nicotinoyl

Table S1. Bioactive lathyrol (**53**) derivatives: biological activities, observed effects and molecular targets (where available).

Comp.	Name(s); (CAS RN)	Activity	Effect	Target
**43**	Euphorbia factor L_3. EFL_3. 5,15-di-*O*-acetyl-3-*O*-benzoyllathyrol. (218916-52-0).	Cytotoxic.[1,2]	- Apoptosis induction.<br>- Disruption normal cell cycle progression.<br>- Actin filament aggregation.<br>- Partial interference of the microtubule network.	Mitocondrial pathway.
		Anti-inflammatory.	Inhibition of NO production.[3,4,5,6]	
		MDR modulator.	Overexpresion P-glycoprotein.[7,8]	P-gp.
**54**	Euphorbia factor L_{15}. EFL_{15}. 3,5,15-Tri-*O*-acetyllathyrol. Euphoboetirane A. (1613699-95-8).	MDR reversal activity.	Strong inhibitory activity of Cdr1 efflux pump.[9]	
			Inhibition of efflux-pump activity.[10]	P-gp.
		NPC proliferation ability.	Increase the size of heurospheres in a	

[1] Zhang, J.Y.; Liang, Y.J.; Chen, H.B.; Zheng, L.S.; Mi, Y.J.; Wang, F.; Zhao, X.Q.; Wang, X.K.; Zhang, H.; Fu, L.W. Structure identification of euphorbia factor L_3 and its induction of apoptosis through the mitochondrial pathway. *Molecules* **2011**, *16*, 3222–3231, doi:10.3390/molecules16043222.

[2] Teng, Y.N.; Wang, Y.; Hsu, P.L.; Xin, G.; Zhang, Y.; Morris-Natschke, S.L.; Goto, M.; Lee, K.H. Mechanism of action of cytotoxic compounds from the seeds of *Euphorbia lathyris*. *Phytomedicine* **2018**, *41*, 62–66, doi:10.1016/j.phymed.2018.02.001.

[3] Zuo, Q.; Mu, H.-Y.; Gong, Q.; Ding, X.; Wang, W.; Zhang, H.-Y.; Zhao, W.-M. Diterpenoids from the seeds of *Euphorbia lathyris* and their effects on microglial nitric oxide production. *Fitoterapia* **2021**, *150*, 104834, doi:https://doi.org/10.1016/j.fitote.2021.104834.

[4] Wang, W.; Wu, Y.; Li, C.; Yang, Y.; Li, X.; Li, H.; Chen, L. Synthesis of New Lathyrane Diterpenoid Derivatives from *Euphorbia lathyris* and Evaluation of Their Anti-Inflammatory Activities. *Chem. Biodivers.* **2020**, *17*, 1–8, doi:10.1002/cbdv.201900531.

[5] Zhang, C.Y.; Wu, Y.L.; Zhang, P.; Chen, Z.Z.; Li, H.; Chen, L.X. Anti-inflammatory Lathyrane Diterpenoids from *Euphorbia lathyris*. *J. Nat. Prod.* **2019**, *82*, 756–764, doi:10.1021/acs.jnatprod.8b00600.

[6] Lee, J.W.; Jin, Q.; Jang, H.; Kim, J.G.; Lee, D.; Kim, Y.; Hong, J.T.; Lee, M.K.; Hwang, B.Y. Lathyrane-Type Diterpenoids from the Seeds of *Euphorbia lathyris* L. with Inhibitory Effects on NO Production in RAW 264.7 Cells. *Chem. Biodivers.* **2018**, *15*, 1–7, doi:10.1002/cbdv.201800144.

[7] Jiao, W.; Wan, Z.; Chen, S.; Lu, R.; Chen, X.; Fang, D.; Wang, J.; Pu, S.; Huang, X.; Gao, H.; et al. Lathyrol diterpenes as modulators of P-glycoprotein dependent multidrug resistance: Structure-activity relationship studies on Euphorbia factor L_3 derivatives. *J. Med. Chem.* **2015**, *58*, 3720–3738, doi:10.1021/acs.jmedchem.5b00058.

[8] Li, X.; Hu, J.; Wang, B.; Sheng, L.; Liu, Z.; Yang, S.; Li, Y. Inhibitory effects of herbal constituents on P-glycoprotein in vitro and in vivo: Herb–drug interactions mediated via P-gp. *Toxicol. Appl. Pharmacol.* **2014**, *275*, 163–175, doi:https://doi.org/10.1016/j.taap.2013.12.015.

[9] Mónico, A.; Nim, S.; Duarte, N.; Rawal, M.K.; Prasad, R.; Di Pietro, A.; Ferreira, M.J.U. Lathyrol and epoxylathyrol derivatives: Modulation of Cdr1p and Mdr1p drug-efflux transporters of Candida albicans in *Saccharomyces cerevisiae* model. *Bioorganic Med. Chem.* **2017**, *25*, 3278–3284, doi:10.1016/j.bmc.2017.04.016.

[10] Neto, S.; Duarte, N.; Pedro, C.; Spengler, G.; Molnár, J.; Ferreira, M.J.U. Effective MDR reversers through phytochemical study of *Euphorbia boetica*. *Phytochem. Anal.* **2019**, *30*, 498–511, doi:10.1002/pca.2841.

Comp.	Name(s); (CAS RN)	Activity	Effect	Target
			dose-dependent manner when proliferation was stimulated by the growth factor EFG and bFGF.[11]	
**91**	Euphorbia factor L_8. EFL_8. 5,15-di-O-acetyl-3-O-nicotinoyllathyrol. (218916-53-1).	Cytotoxic.[2]	- Disruption normal cell cycle progression.<br>- Actin filament aggregation.<br>- Partial interference of the microtubule network.	
		Anti-inflammatory.	Inhibition of NO production.[6]	
**107**	Deoxy-Euphorbia factor L_1. $DEFL_1$. 5,15-di-O-acetyl-3-O-phenylacetyllathyrol. (247099-01-0).	Cytotoxic.	- Cytotoxic activity against lung cancer A549 cells.<br>- Wound healing of A549 cells in a concentration-dependent manner.[12]	Induction of apoptosis, involving of ROS increase, decrease of membrane potential, release of cytochrome c and activity raise of caspase 9 and 3 (mitochondrial pathway).
			Cytotoxic activity against MCF-7 cancer cell line.[15]	
		Anti-inflammatory.	Inhibition of NO production.[6]	
**113**	Euphorbia factor L_{30} EFL_{30}. 15-O-acetyl-3-O-cinnamoyllathyrol.	Cytotoxic.	Cytotoxicity on HEF293T cells.[13]	
			Low cytotoxic activity against MCF-7 cancer cell line (SAR).[14]	

[11] Flores-Giubi, E.; Geribaldi-Doldán, N.; Murillo-Carretero, M.; Castro, C.; Durán-Patrón, R.; MacÍas-Sánchez, A.J.; Hernández-Galán, R. Lathyrane, Premyrsinane, and Related Diterpenes from *Euphorbia boetica*: Effect on in Vitro Neural Progenitor Cell Proliferation. *J. Nat. Prod.* **2019**, *82*, 2517–2528, doi:10.1021/acs.jnatprod.9b00343.

[12] Zhang, J.; Huang, W.; Sun, H.; Liu, Y.; Zhao, X.; Tang, S.; Sun, M.; Wang, S.; Li, J.; Zhang, L.; et al. Structure Identification and In Vitro Anticancer Activity of Lathyrol-3-phenylacetate-5,15-diacetate. *Molecules* 2017, *22*, 1412. doi:10.3390/molecules22091412.

[13] Huang, D.; Wang, R.-M.; Li, W.; Zhao, Y.-Y.; Yuan, F.-Y.; Yan, X.-L.; Chen, Y.; Tang, G.-H.; Bi, H.-C.; Yin, S. Lathyrane Diterpenoids as Novel hPXR Agonists: Isolation, Structural Modification, and Structure–Activity Relationships. *ACS Med. Chem. Lett.* **2021**, *12*, 1159–1165, doi:10.1021/acsmedchemlett.1c00277.

[14] Wang, Q.; Zhen, Y.Q.; Gao, F.; Huang, S.; Zhou, X.L. Five New Diterpenoids from the Seeds of *Euphorbia lathyris*. *Chem. Biodivers.* **2018**, *15*, 9–16, doi:10.1002/cbdv.201800386.

Comp.	Name(s); (CAS RN)	Activity	Effect	Target
	(2*S*,3S,4*S*,5*R*,9*S*,11*R*,15*R*)-15-Acetoxy-3-cinnamoyloxy-5-hydroxylathyra-6(17),12*E*-dien-14-one. (2293060-22-5).	Anti-inflammatory.	Inhibition of NO production.[5]	
**121**	Euphorbia factor L_{31} EFL_{31}. (2293060-23-6).	Cytotoxic.	Cytotoxicity on HEF293T cells.[13]	
		Anti-inflammatory.	Inhibition of NO production.[3]	
**129**	Euphorbia factor L_{32} EFL_{32}.	Anti-inflammatory.[42]	Inhibitory effect on NO production in LPS-induced RAW264.7 macrophage cells.	
**152**	Euphlathyrinoid D. 5,15-Di-O-acetyl-3-*O*-cinnamoyllathyrol. (2750897-06-2).	Anti-cholestasis.	Activation of Phase I, Phase I metabolism and phase III efflux transporters.[13]	Pregnane X receptor (PXR).
**S1**	Euphorbia factor L_{16}. EFL_{16}. 5,15-di-*O*-acetyl-3-*O*-salicyloyllathyrol. (1613699-97-0).	Cytotoxic.	Cytotoxic activity against C-6 cancer cell line.[15]	
**S2**	2-*epi*-Lathyrol triacetate. (2*R**,3*S**,4*R**,5*R**,9*S**,11*S**,15*R**)-3,5,15-Triacetoxy-14-oxolathyra-6(17),12*E*-diene. (489459-81-6).	MDR Modulation.	Inhibition of the efflux-pump activity.[16]	P-gp.
			MDR modulation on colon cancer cells.[17]	
**44**	5-*O*-Phenylacetyllathyrol. (1708993-46-7).	MDR modulation.	Inhibition the efflux-pump activity.[7]	P-gp.
**45**	5-*O*-(1-Naphtyl)-acetyllathyrol. (1708993-49-0).			
**S3**	5-*O*-Acetyllathyrol. (1708993-38-7).	MDR modulation.	Inhibition the efflux-pump activity.[7]	P-gp.
**S4**	5-*O*-Propionyllathyrol. (1708993-39-8).			
**S5**	5-*O*-*n*-Valeryllathyrol.			

[15] Lu, J.; Li, G.; Huang, J.; Zhang, C.; Zhang, L.; Zhang, K.; Li, P.; Lin, R.; Wang, J. Lathyrane-type diterpenoids from the seeds of *Euphorbia lathyris*. *Phytochemistry* **2014**, *104*, 79–88, doi:10.1016/j.phytochem.2014.04.020.
[16] Sousa, I.J.; Ferreira, M.J.U.; Molnár, J.; Fernandes, M.X. QSAR studies of macrocyclic diterpenes with P-glycoprotein inhibitory activity. *Eur. J. Pharm. Sci.* **2013**, *48*, 542–553, doi:10.1016/j.ejps.2012.11.012.
[17] Engi, H.; Vasas, A.; Rédei, D.; Molnár, J.; Hohmann, J. New MDR modulators and apoptosis inducers from *Euphorbia* species. *Anticancer Res.* **2007**, *27*, 3451–3458.

Comp.	Name(s); (CAS RN)	Activity	Effect	Target
	(1708993-40-1).			
**S6**	5-*O*-*n*-Hexanoyllathyrol. (1708993-41-2).			
**S7**	5-*O*-Isobutyroyllathyrol. (1708993-42-3).			
**S8**	5-*O*-(3-Methyl)butanoyllathyrol. (1708993-43-4).			
**S9**	5-*O*-Nicotinoyllathyrol. (1708993-44-5).			
**S10**	5-*O*-Benzoyllathyrol. (1708993-45-6).			
**S11**	5-*O*-(3-Phenyl)-propionyllathyrol. (1708993-47-8).			
**S12**	5-*O*-Cinnamoyllathyrol. (1708993-48-9).			
**46**	3,5-di-*O*-Benzoyllathyrol. (1708993-56-9).	MDR modulation.	Inhibition the efflux-pump activity.[7]	P-gp.
**47**	3-*O*-Propanoyl-5-*O*-benzoyllathyrol. (1708993-61-6).			
**48**	3-*O*-Propanoyl-5-*O*-phenylacetyllathyrol. (1708993-62-7).			
**49**	3-*O*-Propanoyl-5-*O*-(1-naphtyl)-acetyllathyrol. (1708993-63-8).			
**50**	3-*O*-Cinnamoyl-5-*O*-benzoyllathyrol. (1708993-65-0).			
**132**	3,5-Di-*O*-cinnamoyllathyrol. (2473033-45-1).	Anti-inflammatory.	Inhibition of NO production.[4]	
**S13**	3,5-di-*O*-Acetyllathyrol. (35556-35-5).	MDR modulation.	Inhibition the efflux-pump activity.[7]	P-gp.
**S14**	3,5-di-*O*-Propionyllathyrol. (1708993-52-5).			

Comp.	Name(s); (CAS RN)	Activity	Effect	Target
**S15**	3,5-di-*O*-*n*-Butanoyllathyrol. (1708993-53-6).			
**S16**	3,5-di-*O*-*n*-Hexanoyllathyrol. (1708993-55-8).			
**S17**	3,5-di-*O*-Phenylacetyllathyrol. (1708993-58-1).			
**S18**	3,5-di-*O*-(3-phenyl)-propanoyllathyrol. (1708993-59-2).			
**S19**	3,5-di-*O*-(1-Naphtyl)-acetyllathyrol. (1708993-60-5).			
**S20**	3-*O*-Benzoyl-5-*O*-(1-naphtyl)-acetyllathyrol. (1708993-64-9).			
**55**	Euphoboetirane B.	MDR reversal activity.	Inhibition of efflux-pump activity.[10]	P-gp.
**56**	Euphoboetirane C.			
**57**	Euphoboetirane D.			
**58**	Euphoboetirane E.			
**59**.	Euphoboetirane F.			
**60**	Euphoboetirane G.			
**61**	Euphoboetirane H.			
**62**	Euphoboetirane I.			
**63**	Euphoboetirane J. (1708993-45-6).	MDR reversal activity.	Strong inhibitory activity of Cdr1 efflux pump.[9]	
**64**	Euphoboetirane K. (2101545-30-4).			
**65**	Euphoboetirane L. (2101545-32-6).			
**68**	Euphoboetirane O. (1708993-48-9).			
**78**	(7*R*)-5,15-Di-*O*-acetyl-3-*O*-cinnamoyl-7-hydroxylathyrol.	MDR reversal ability.	Inhibition of the efflux-pump activity.[18]	P-gp.
**79**	(7*R*)-5,15-Di-*O*-acetyl-3-*O*-nicotinoyl-7-hydroxylathyrol.			

[18] Yang, T.; Wang, S.; Li, H.; Zhao, Q.; Yan, S.; Dong, M.; Liu, D.; Chen, X.; Li, R. Lathyrane diterpenes from Euphorbia lathyris and the potential mechanism to reverse the multi-drug resistance in HepG2/ADR cells. *Biomed. Pharmacother.* 2020, *121*, 109663, doi:10.1016/j.biopha.2019.109663.

Section S2. Bioactive 7-hydroxylathyrol (**92**) and 7-oxolathyrol derivatives.

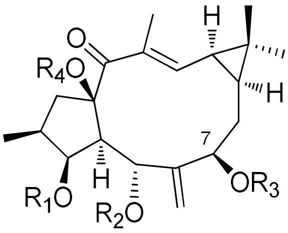

77-80, 92, 95, 115-117, 120, 131-132, S21

Compound	R_1	R_2	R_3	R_4	CAS RN	Common name
**77**	Bz	Ac	Bz	Ac	218916-51-9	Euphorbia factor L_2
**80**	Bz	Ac	Nic	Ac		Euphorbia factor L_9
**92**	H	H	H	H	34208-98-5	7-Hydroxylathyrol
**95**	Bz	Ac	Nic	H	2688832-08-6	Euphorbia factor L_{28}
**114**	Bz	H	Ac	Ac	2359682-49-6	-
**115**	Bz	Ac	H	Ac	2359682-50-9	-
**116**	Bz	Ac	Bz	H	850560-45-1	Euphorbia factor L_{11}.
**119**	Bz	H	Bz	H	-	-
**130**	Bz	Ac	Isonic	Ac	2561486-24-4	-
**131**	Bz	Ac	Glyc	Ac	2688017-77-6	-
**S21**	Bz	H	Bz	Ac	-	-

Ac=Acetyl; Bz= Benzoyl; Cin=Cinnamoyl; Isonic = isonicotinyl; Glyc = glycinoyl; Nic=nicotinyl; *p*-CF_3Bz = *p*-trifluoromethylbenzoyl; *m*-MeOBz = *m*-methoxybenzoyl; PhAc=phenylacetate.

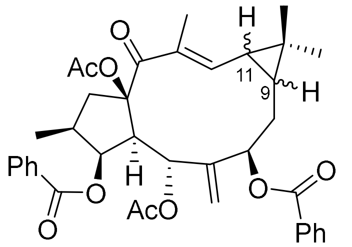

9β,11β (**118**)
9β,11α, EFL_{2b} (**124**)
(CAS RN: 2570893-48-8)

9α,11β (**125**)
9α,11α EFL_2 (**77**)
(CAS RN: 218916-51-9)

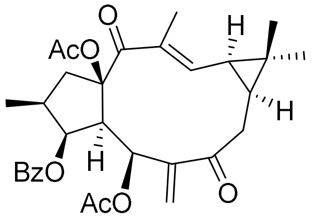

Euphlathyrinoid C (**150**)
(CAS RN: 2750897-03-9)

Table 2. Bioactive 7-hydroxylathyrol (**92**) and 7-oxolathyrol derivatives: biological activities, observed effects and molecular targets (where available).

Comp.	Name(s); (CAS RN)	Activity	Effect	Target
**77**	Euphorbia factol L_2. EFL2. (7*R*)-5,15-di-*O*-acetyl-3,7-di-*O*-benzoyl-7-hydroxylathyrol. (218916-51-9).	MDR modulation.	Inhibition the efflux-pump activity.	P-gp.
		Anti-inflammatory.	Inhibition of NO production.[6,19]	
			Decreasing of the levels of IL-1 b, IL-6, TNF-a, IL-8 and MPO in the lung and bronchioalveolar lavage fluid.[19]	NF- B
		Cytotoxic.	Inhibition of TGF-β-induced cell growth and migration of hepatocellular carcinoma.[20]	AKT/STAT3.
			Apoptosis.[21]	Mitocondrial pathway.
		Anti-cholestasis.	Activation of Phase I, Phase I metabolism and phase III efflux transporters.[13]	Pregnane X receptor (PXR).
**80**	Euphorbia factor L_9. EFL9. (7*R*)-5,15-di-*O*-acetyl-3-*O*-benzoyl-7-*O*-nicotinoyl-hydroxylathyrol. (129393-28-8).	Anti-cholestasis.	Activation of Phase I, Phase I metabolism and phase III efflux transporters.[13]	Pregnane X receptor (PXR).
		Anti-inflammatory.	Inhibition of NO production.[4,5,6]	
		Cytotoxic.[2]	Disruption normal cell cycle progression. Actin filament aggregation. Partial interference of the microtubule network.	
		MDR reversal ability	Inhibition of the efflux-pump activity.[18]	P-gp.
**95**	Euphorbia factor L_{28}.	Antiproliferative.	Cytotoxicity to the breast cancer 786-0 and	

[19] Zhang, Q.; Zhu, S.; Cheng, X.; Lu, C.; Tao, W.; Zhang, Y.; William, B.C.; Cao, X.; Yi, S.; Liu, Y.; et al. Euphorbia factor L_2 alleviates lipopolysaccharide-induced acute lung injury and inflammation in mice through the suppression of NF-κB activation. *Biochem. Pharmacol.* **2018**, *155*, 444–454, doi:10.1016/j.bcp.2018.07.025.

[20] Lu, F.; Huayun, Z.; Weiwei, T.; Li, L.; Xin, S.; Ming, Z.; Dongdong, S. Euphorbia factor L_2 inhibits TGF-β-induced cell growth and migration of hepatocellular carcinoma through AKT/STAT3. *Phytomedicine* **2019**, *62*, 152931, doi:10.1016/j.phymed.2019.152931.

[21] Lin, M.; Tang, S.; Zhang, C.; Chen, H.; Huang, W.; Liu, Y.; Zhang, J. Euphorbia factor L_2 induces apoptosis in A549 cells through the mitochondrial pathway. *Acta Pharm. Sin. B* **2017**, *7*, 59–64, doi:10.1016/j.apsb.2016.06.008.

Comp.	Name(s); (CAS RN)	Activity	Effect	Target
	EFL28. (2688832-08-6).		liver cancer HepG2 cell lines.[22]	
**114**	(2*S*,3*S*,4*S*,5*R*,7*R*,9*S*,11*R*,15*R*)-7,15-Diacetoxy-3-benzoyloxy-5-hydroxylathyra-6(17),12*E*-dien-14-one. (2359682-49-6).	Anti-inflammatory.	Inhibition of NO production.[5]	
**115**	(2S,3S,4R,5R,7R,9S,11R,15R)-5,15-Diacetoxy-3-benzoyloxy-7-hydroxylathyra-6(17),12E-dien-14-one. (2359682-50-9).	Anti-inflammatory.	Inhibition of NO production.[5]	
		MDR modulator	Inhibition of the efflux-pump activity.[23]	P-gp
**116**	Euphorbia factor L_{11}. EFL11. (7*R*)-5-*O*-acetyl-3,7-di-*O*-benzoyl-7-hydroxylathyrol. (850560-45-1).	Anti-inflammatory.	Inhibition of NO production.[5]	
**118**	(7*R*,9*R*,11*S*)-5,15-di-*O*-Acetyl-3,7-di-*O*-benzoyl-17-hydroxylathyrol.	Anti-inflammatory.	Inhibition of NO production.[3]	
**119**	(7*R*)-3,7-di-*O*-Benzoyl-17-hydroxylathyrol.	Anti-inflammatory.	Inhibition of NO production.[3]	
**124**	Euphorbia factor L_{2b}. EFL_{2b}. (7*R,*9*R*)-5,15-di-*O*-Acetyl-3,7-di-*O*-benzoyl-17-hydroxylathyrol. (2570893-48-8).	Cytotoxic.	Inhibitory effect against acute myeloid leukemia U937 cell line.[24]	
		Anti-inflammatory.	Inhibition of NO production.[3]	
**125**	(7*R*,11*S*)-3,7-di-*O*-Benzoyl-17-hydroxylathyrol.	Anti-inflammatory.	Inhibition of NO production.[3]	
**130**	(7*R*)-5,15-di-*O*-acetyl-3-*O*-benzoyl-	Anti-inflammatory.	Inhibition of NO production.[4]	

[22] Wang, J.X.; Wang, Q.; Zhen, Y.Q.; Zhao, S.M.; Gao, F.; Zhou, X.L. Cytotoxic lathyrane-type diterpenes from seeds of *Euphorbia lathyris*. *Chem. Pharm. Bull.* **2018**, *66*, 674–677, doi:10.1248/cpb.c17-00946.
[23] Jiao, W.; Dong, W.; Li, Z.; Deng, M.; Lu, R. Lathyrane diterpenes from *Euphorbia lathyris* as modulators of multidrug resistance and their crystal structures. *Bioorganic Med. Chem.* **2009**, *17*, 4786–4792, doi:10.1016/j.bmc.2009.04.041.
[24] Li, L.; Huang, J.; Lyu, H.; Guan, F.; Li, P.; Tian, M.; Xu, S.; Zhao, X.; Liu, F.; Paetz, C.; et al. Two lathyrane diterpenoid stereoisomers containing an unusual: trans-gem -dimethylcyclopropane from the seeds of *Euphorbia lathyris*. *RSC Adv.* **2021**, *11*, 3183–3189, doi:10.1039/d0ra10724g.

Comp.	Name(s); (CAS RN)	Activity	Effect	Target
	7-*O*-isonicotinoyl-hydroxylathyrol. (2561486-24-4).			
**131**	(7*R*)-5,15-di-*O*-acetyl-3-*O*-benzoyl-7-*O*-glycinoyl-hydroxylathyrol. (2688017-77-6).			
**150**	Euphlathyrinoid C. (2750897-03-9).	Cytotoxic.	Cytotoxicity on HEF293T cells.[13]	
**S21**	(7*R*)-15-*O*-Acetyl-3,7-di-*O*-benzoyl-7-hydroxylathyrol.	MDR reversal ability.	Inhibition of the efflux-pump activity.[18**Error! Bookmark not defined.**]	P-gp.

Section S3. Bioactive 12-hydroxylathyrol derivatives.

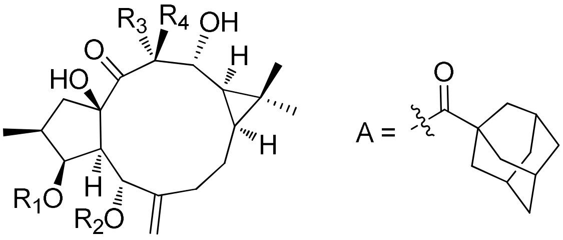

66-67, 69-72

Compound	R_1	R_2	R_3	R_4	CAS RN	Common name
**66**	H	*m*-BrBz	CH_3	H	2101545-34-8	Euphoboetirane M
**67**	H	*m*-CF_3Bz	CH_3	H	2101545-36-0	Euphoboetirane N
**69**	H	Naph	H	CH_3	-	12-Hydroxyboetirane A
**70**	*p*-CF_3Bz	H	H	CH_3	-	12-Hydroxyboetirane B
**71**	H	*p*-CF_3Bz	H	CH_3	-	12-Hydroxyboetirane C
**72**	H	A	H	CH_3	-	12-Hidroxyboetirane D

m-BrBz = *m*-bromobenzoyl; *m*-CF_3Bz = *m*-trifluoromethylbenzoyl; *p*-CF_3Bz = *p*-trifluoromethylbenzoyl; Naph = 2-naphthoyl.

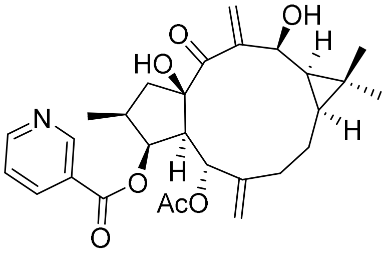

S22

Table S3. Bioactive 12-hydroxylathyrol derivatives: biological activities, observed effects and molecular targets (where available).

Comp.	Name(s); (CAS RN)	Activity	Effect	Target
**69**	12-Hydroxyboetirane A.	MDR reversal activity.	Inhibition of efflux-pump activity.[10]	P-gp.
**70**	12-Hydroxyboetirane B.			
**71**	12-Hydroxyboetirane C.			
**72**	12-Hydroxyboetirane D.			
**66**	Euphoboetirane M. (2101545-34-8).	MDR reversal activity.	Strong inhibitory activity of Cdr1 efflux pump.[9]	
**67**	Euphoboetirane N. (2101545-36-0).	MDR reversal activity.[9]	- Strong inhibitory activity of Cdr1 efflux pump.<br>- Synergistic effect in the AD-CDR1 yeast strain by reducing the effective concentration of fluconazole as much as 52-fold.<br>- Overexpression the Cdr1p transporter.	
**S22**	5,15-diacetoxy-3-nicotinoyloxy-12-hydroxylathyra-6(17),13(20)-dien-14-one	MDR reversal ability.	Inhibition of the efflux-pump activity.[18]	P-gp.

Section S4. Bioactive 12,15-epoxylathyrol derivatives.

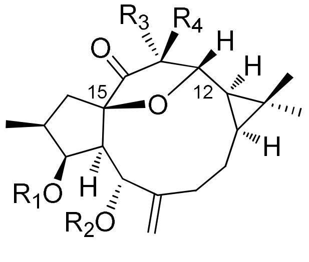

S(23-33)

Compound	R_1	R_2	R_3	R_4	CAS RN
**S23**	Bz	Bz	CH_3	H	1708993-67-2
**S24**	PhAc	PhAc	CH_3	H	1708993-68-3
**S25**	PhPr	PhPr	CH_3	H	1708993-69-4
**S26**	Ac	Ac	H	CH_3	1708993-70-7
**S27**	*n*-Bu	*n*-Bu	H	CH_3	1708993-71-8
**S28**	*n*-Hex	*n*-Hex	H	CH_3	1708993-72-9
**S29**	Bz	Bz	H	CH_3	1708993-73-0
**S30**	PhAc	PhAc	H	CH_3	1708993-74-1
**S31**	PhPr	PhPr	H	CH_3	1708993-75-2
**S32**	Cin	Cin	H	CH_3	1708993-76-3
**S33**	NpAc	NpAc	H	CH_3	1708993-77-4

Ac = acetyl; *n*-Bu = n-butyroyl; Bz = benzoyl; Cin = cinammoyl; *n*-Hex = hexanoyl; NpAc = 1-naphthylacetyl; PhAc = phenylacetyl; PhPr = 3-phenylpropionyl.

Table S4. Bioactive 12,15-epoxylathyrol derivatives: biological activities, observed effects and molecular targets (where available).

Comp.	Name(s); (CAS RN)	Activity	Effect	Target
**S23**	(2*S*,3*S*,4*R*,5*R*,9*S*,11*R*,12*S*,13*R*,15*R*)-3,5-di-*O*-Benzoyl-12,15-epoxylathyra-6(17)-en-14-one. (1708993-67-2).	MDR modulation.	Inhibition the efflux-pump activity.[7]	P-gp.
**S24**	(2*S*,3*S*,4*R*,5*R*,9*S*,11*R*,12*S*,13*R*,15*R*)-3,5-di-*O*-Phenylacetyl-12,15-epoxylathyra-6(17)-en-14-one. (1708993-68-3).			
**S25**	(2*S*,3*S*,4*R*,5*R*,9*S*,11*R*,12*S*,13*R*,15*R*)-3,5-di-*O*-Phenylpropionyl-12,15-epoxylathyra-6(17)-en-14-one. (1708993-69-4).			
**S26**	(2*S*,3*S*,4*R*,5*R*,9*S*,11*R*,12*S*,13*S*,15*R*)-3,5-di-*O*-Acetyl-12,15-epoxylathyra-6(17)-en-14-one. (1708993-70-7).			
**S27**	(2*S*,3*S*,4*R*,5*R*,9*S*,11*R*,12*S*,13*S*,15*R*)-3,5-di-*O*-Butyryl-12,15-epoxylathyra-6(17)-en-14-one. (1708993-71-8).			
**S28**	(2*S*,3*S*,4*R*,5*R*,9*S*,11*R*,12*S*,13*S*,15*R*)-3,5-di-*O*-Hexanoyl-12,15-epoxylathyra-6(17)-en-14-one. (1708993-72-9).			
**S29**	(2*S*,3*S*,4*R*,5*R*,9*S*,11*R*,12*S*,13*S*,15*R*)-3,5-di-*O*-Benzoyl-12,15-epoxylathyra-6(17)-en-14-one. (1708993-73-0).			
**S30**	(2*S*,3*S*,4*R*,5*R*,9*S*,11*R*,12*S*,13*S*,15*R*)-3,5-di-*O*-Phenylacetyl-12,15-epoxylathyra-6(17)-en-14-one. (1708993-74-1).			
**S31**	(2*S*,3*S*,4*R*,5*R*,9*S*,11*R*,			

Comp.	Name(s); (CAS RN)	Activity	Effect	Target
	12*S*,13*S*,15*R*)-3,5-di-*O*-Phenylpropionyl-12,15-epoxylathyra-6(17)-en-14-one. (1708993-75-2).			
**S32**	(2*S*,3*S*,4*R*,5*R*,9*S*,11*R*,12*S*,13*S*,15*R*)-3,5-di-*O*-Cinnamoyl-12,15-epoxylathyra-6(17)-en-14-one. (1708993-76-3).	MDR modulation.	Inhibition the efflux-pump activity.[7]	P-gp.
**S33**	(2*S*,3*S*,4*R*,5*R*,9*S*,11*R*,12*S*,13*S*,15*R*)-3,5-di-*O*-(1-Naphtyl)acetyl-12,15-epoxylathyra-6(17)-en-14-one. (1708993-77-4).			

Section S5. Bioactive 6,17-epoxylathyrol (epoxylathyrol (**10**)) derivatives.

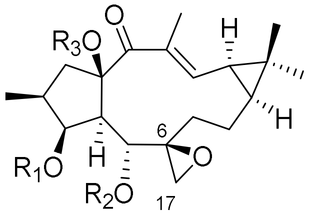

10-20, 76, 82, 112, 121, S(40-44)

Compound	R1	R2	R3	CAS RN	Common Name
**10**	H	H	H	304021-51-0	Epoxylathyrol
**11**	Ac	Ac	Ac	1637571-63-1	Epoxyboetirane A
**S40**	3-MeBu	Ac	Ac	1637571-64-2	Epoxyboetirane B
**12**	*p*-MeBz	*p*-MeBz	H	1637571-65-3	Epoxyboetirane C
**13**	*p*-MeOBz	*p*-MeOBz	H	1637571-66-4	Epoxyboetirane D
**14**	Bz	Bz	H	1637571-67-5	Epoxyboetirane E
**15**	Bz	Bz	Bz	1637571-68-6	Epoxyboetirane F
**S41**	Cin	H	H	1637571-69-7	Epoxyboetirane G
**16**	PhAc	PhAc	H	1637571-70-0	Epoxyboetirane H
**17**	2-EtHe	2-EtHe	H	1637571-71-1	Epoxyboetirane I
**18**	Bu	Bu	Bu	1637571-72-2	Epoxyboetirane J
**19**	Pr	Ac	Ac	1443058-39-6	Epoxyboetirane K
**20**	Bu	Ac	Ac	1637571-38-5	Epoxyboetirane L
**S42**	Bz	Ac	Ac	303174-98-3	Epoxyboetirane M
**76**	PhAc	Ac	Ac	76376-43-7	Euphorbia factor L_1
**82**	Nic	Ac	Ac	2056283-85-1	Euphordracunculin C
**112**	Bz	Ac	Ac	303174-98-3	Euphorbia factor L_{25}.
**120**	PhAc	Ac	Ac	-	-
**S43**	PhAc	H	Ac	-	-
**S44**	Nic	Ac	Ac	-	-

Ac = acety; Bu = butiryl; Bz = benzoyl; 2-EtHe = 2-ethylhexanoyl; 3-MeBu = 3-methylbutyryl; *p*-MeBz = *p*-methylbenzoyl; *p*-MeOBz = *p*-methoxybenzoyl; Nic = nicotinoyl; PhAc = phenylacetyl.

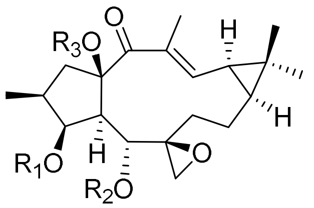

21-30, S45

Compound	R1	R2	R3	CAS RN	Common name
**21**	BiPhCO	BiPhCO	Ac	1809418-74-3	-
**22**	NaphCO	NaphCO	H	1809418-75-4	-
**23**	*m*-MeBz	*m*-MeBz	H	1809418-76-5	-
**24**	*m*-MeOBz	*m*-MeOBz	H	1809418-77-6	Epoxyboetirane N
**25**	*p*-CF_3Bz	*p*-CF_3Bz	H	1809418-78-7	Epoxyboetirane O
**26**	*p*-Me_2NBz	*p*-Me_2NBz	H	1809418-79-8	Epoxyboetirane P
**27**	2-FuCO	2-FuCO	H	1809418-80-1	Epoxyboetirane Q
**28**	2-ThPheCO	2-ThPheCO	H	1809418-81-2	Epoxyboetirane R
**S45**	1-AdCO	1-AdCO	H	1809418-82-3	Epoxyboetirane S
**29**	Et_2NCO	Et_2NCO	H	1809418-86-7	Epoxycarbamoylboetirane B
**30**	4-MorphCO	4-MorphCO	H	1809418-87-8	Epoxycarbamoylboetirane C

BiPhCO = biphenylcarbonyl; Et_2NCO=diethylcarbamoyl; 4-MorphCO = morpholine-4-carbonyl; NaphCO = Naphtylcarbonyl; *m*-MeBz = *m*-methylbenzoyl; *m*-MeOBz = *m*-methoxybenzoyl; *p*-CF_3Bz = *p*-trifluoromethylbenzoyl; *p*-Me_2NBz = *p*-dimethyaminobenzoyl; 2-FuCO = 2-furanecarbonyl; 2-ThPh = 2-thiophenecarbonyl; 1-AdCO = 1-adamantanecarbonyl.

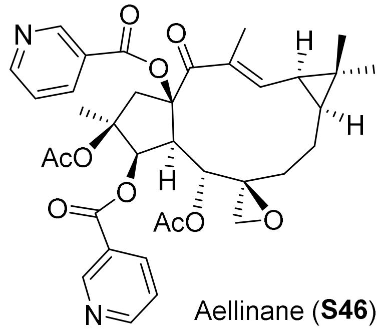

Aellinane (**S46**)

Table S5. Bioactive 6,17-epoxylathyrol (epoxylathyrol (**10**)) derivatives: biological activities, observed effects and molecular targets (where available).

**Comp.**	**Name(s); (CAS RN)**	**Activity**	**Effect**	**Target**
**10**	Epoxylathyrol. (304021-51-0).	MDR reversal ability	Inhibition of drug-efflux activity of Cdr1 and Mdr1p transporters of *Candida albicans* overexpressed in a *Saccharomyces cerevisiae* strain.[9]	
**11**	Epoxyboetirane A. 3β,5α,15β-triacetoxy-6(17)-epoxylathyr-12*E*-en-14-one. (1637571-64-1).	Anti-inflammatory.	Inhibition of NO production.[6]	
		MDR reversal ability.	Inhibition of the efflux-pump activity.[25,26]	
			Inhibition of drug-efflux activity of Cdr1 and Mdr1p transporters of *Candida albicans* overexpressed in a *Saccharomyces cerevisiae* strain.[9]	
		NPC proliferation ability.[11]	Increase the size of heurospheres in a dose-dependent manner when proliferation was stimulated by the growth factors EFG and bFGF.	
**S40**	Epoxyboetirane B. 5α,15β-diacetoxy-3β-(3-methyl)butanoyloxy-6(17)-epoxylathyr-12*E*-en-14-one. (1637571-64-2).	MDR reversal ability.	Inhibition of the efflux-pump activity.[26]	P-gp.
**12**	Epoxyboetirane C. 3β,5α-di(4-methyl-benzoyloxy)-15β-hydroxy-6(17)-epoxylathyr-12*E*-en-14-one. (1637571-65-3).	MDR reversal ability.	Inhibition of the efflux-pump activity.[26]	P-gp.
**13**	Epoxyboetirane D.		Inhibition of drug-efflux activity of Cdr1	

[25] Baptista, R.; Ferreira, R.J., Dos Santos, Daniel, J.; Fernandes, M.; Ferreira, M. Optimizing the macrocyclic diterpenic core toward the reversal of multidrug resistance in cancerNo Title. *Future Med. Chem.* **2016**, *8*, 629–645.

[26] Vieira, C.; Duarte, N.; Reis, M.A.; Spengler, G.; Madureira, A.M.; Molnár, J.; Ferreira, M.J.U. Improving the MDR reversal activity of 6,17-epoxylathyrane diterpenes. *Bioorganic Med. Chem.* **2014**, *22*, 6392–6400, doi:10.1016/j.bmc.2014.09.041.

Comp.	Name(s); (CAS RN)	Activity	Effect	Target
	3β,5α-di(4-methoxy-benzoyloxy)-15β-hydroxy-6(17)-epoxylathyr-12*E*-en-14-one. (1637571-66-4).		and Mdr1p transporters of *Candida albicans* overexpressed in a *Saccharomyces cerevisiae* strain.[9]	
**14**	Epoxyboetirane E. 3β,5α-dibenzoyloxy-15β-hydroxy-6(17)-epoxylathyr-12*E*-en-14-one. (1637571-67-5).	MDR reversal ability.	Inhibition of the efflux-pump activity.[26,27]	P-gp.
			Inhibition of drug-efflux activity of Cdr1 and Mdr1p transporters of *Candida albicans* overexpressed in a *Saccharomyces cerevisiae* strain. [9]	
		MDR-selective antiproliferative activity, mostly in drug-resistant gastric sublines.[27]	- Enhancement of the cytotoxicity of doxorubicin in a synergistic mode. -Restoration sensitivity by reversion of the ABCD1_MDR phenotype.	
**15**	Epoxyboetirane F. 3β,5α,15β-tribenzoyloxy-6(17)-epoxylathyr- 12*E*-en-14-one. (1637571-68-6).	MDR reversal ability.	Inhibition of the efflux-pump activity. [26]	P-gp
			Inhibition of drug-efflux activity of Cdr1 and Mdr1p transporters of *Candida albicans* overexpressed in a *Saccharomyces cerevisiae* strain. [9]	
**S41**	Epoxyboetirane G. 3β–cinnamoyloxy-5α, 15β-dihydroxy-6(17)-epoxylathyr-12*E*-en-14-one. (1637571-69-7).	MDR reversal ability.	Inhibition of the efflux-pump activity. [26]	P-gp.
**16**	Epoxyboetirane H. 15β-hydroxy-3β,5α-diphenylacetyl-6(17)-epoxylathyr-12E-en-14-one. (1637571-70-0).	MDR reversal ability.	Inhibition of the efflux-pump activity. [26]	P-gp.
			Inhibition of drug-efflux activity of Cdr1 and Mdr1p transporters	

[27] Reis, M.A.; Matos, A.M.; Duarte, N.; Ahmed, O.B.; Ferreira, R.J.; Lage, H.; Ferreira, M.J.U. Epoxylathyrane Derivatives as MDR-Selective Compounds for Disabling Multidrug Resistance in Cancer. *Front. Pharmacol.* **2020**, *11*, 1–11, doi:10.3389/fphar.2020.00599.

Comp.	Name(s); (CAS RN)	Activity	Effect	Target
**17**	Epoxyboetirane I. 3β,5α-di(2-ethylhexanoyl)- 15β-hydroxy-6(17)-epoxylathyr-12*E*-en-14-one. (1637571-71-1).		of *Candida albicans* overexpressed in a *Saccharomyces cerevisiae* strain. [9]	
**18**	Epoxyboetirane J. 3β,5α,15β-tributanoyloxy-6(17)-epoxylathyr-12*E*-en-14-one. (1637571-72-2).	MDR reversal ability.	Inhibition of the efflux-pump activity transporters of Candida albicans overexpressed in a Saccharomyces cerevisiae strain. [26]	P-gp.
			Inhibition of Cdr1p efflux pump.[9]	
**19**	Epoxyboetirane K. 3β-propionyloxy-5α,15β-diacetoxy-6,17- epoxy-lathyra-12*E*-en-14-one. (1443058-39-6).	MDR reversal ability.	Inhibition of the efflux-pump activity. [26]	P-gp.
			-Synergistic effect with fluconazole in the AD-CDR1 yeast strain. -Overexpression the Cdr1p transporter -Ability to reduce the effective concentration of the fluconazole by 23-fold. [9]	
		Antiinflamatory	Inhibition of NO production.[28]	
**20**	Epoxyboetirane L. (1443058-38-5).	Antiinflamatory	Inhibition of NO production.[28]	
		MDR reversal ability	Inhibition of the efflux-pump activity. [26]	P-gp
			Inhibition of drug-efflux activity of Cdr1 and Mdr1p transporters of *Candida albicans* overexpressed in a *Saccharomyces cerevisiae* strain. [9]	
**S42**	Epoxyboetirane M. (303174-98-3).	MDR reversal ability	Inhibition of the efflux-pump activity. [26]	P-gp

[28] Xu, J.; Jin, D.Q.; Song, H.; Guo, Y.; He, Y. Lathyrane diterpenes from *Euphorbia prolifera* and their inhibitory activities on LPS-induced NO production. *Fitoterapia* **2012**, *83*, 1205–1209, doi:10.1016/j.fitote.2012.06.014.

Comp.	Name(s); (CAS RN)	Activity	Effect	Target
		Antiinflamatory	Inhibition of NO production.[28]	
			Inhibition of NO production in LPS-induces RAW 264,7 macrophages.[6]	
**76**	Euphorbia Factor L_1. EFL1. (6*S*)-5,15-di-*O*-acetyl-6,17-epoxy-3-*O*-phenylacetyl-lathyrol. (76376-43-7)	Anticancer.[15,29]	Cytotoxicity to KB, KBv200, MCF-7, C6 and MCF-7/ADR cells.	
		MDR modulator	Inhibition of the efflux-pump activity.[23]	P-gp
		MDR reversal ability.[30,31]	- Apoptosis induction.<br>- Elevated sensitivity to chemo-therapeutical drug.<br>- Apopotosis sensitization induced.	VCR mitocondrial pathway.
		Intestinal toxicity.	Intestinal impairment Induction.[32]	Gene expression and GABAergic neurons.
			- Oxidative stress.<br>- Mitochondria-mediated apoptosis.<br>- Autophagy<br>- Inhibition of the PI3K/AKT/mTOR pathway.[33]	Human proteins of apoptotic pathway and PI3K/AKT/mTOR pathway.
**82**	Euphordracunculin C. (2056283-85-1).	Anti-inflammatory.	Inhibition of NO production.[6]	

[29] Zhang, J.Y.; Zhang, C.; Chen, H.B.; Fu, L.W.; Tao, Y.W.; Zheng, X.Q.; Cao, Z.M.; Zhong, Y.F.; Yu, L.H. Assignments of ^{1}H and ^{13}C NMR signals of Euphorbia factor L_1 and investigation of its anticancer activity in vitro. *J. Med. Plants Res.* **2010**, *4*, 335–338, doi:10.5897/JMPR09.473.

[30] Zhang, J.Y.; Mi, Y.J.; Chen, S.P.; Wang, F.; Liang, Y.J.; Zheng, L.S.; Shi, C.J.; Tao, L.Y.; Chen, L.M.; Chen, H.B.; et al. Euphorbia factor L_1 reverses ABCB1-mediated multidrug resistance involving interaction with ABCB1 independent of ABCB1 downregualtion. *J. Cell. Biochem.* **2011**, *112*, 1076–1083, doi:10.1002/jcb.23021.

[31] Zhang, J.Y.; Lin, M.T.; Yi, T.; Tang, Y.N.; Fan, L.L.; He, X.C.; Zhao, Z.Z.; Chen, H.B. Apoptosis sensitization by euphorbia factor L_1 in ABCB1-mediated multidrug resistant K562/ADR cells. *Molecules* **2013**, *18*, 12793–12808, doi:10.3390/molecules181012793.

[32] Zhu, A.; Ji, Z.; Zhao, J.; Zhang, W.; Sun, Y.; Zhang, T.; Gao, S.; Li, G.; Wang, Q. Effect of Euphorbia factor L_1 on intestinal barrier impairment and defecation dysfunction in *Caenorhabditis elegans*. *Phytomedicine* **2019**, *65*, doi:10.1016/j.phymed.2019.153102.

[33] Zhu, A.; Sun, Y.; Zhong, Q.; Yang, J.; Zhang, T.; Zhao, J.; Wang, Q. Effect of euphorbia factor L_1 on oxidative stress, apoptosis, and autophagy in human gastric epithelial cells. *Phytomedicine* **2019**, *64*, 152929, doi:10.1016/j.phymed.2019.152929.

Comp.	Name(s); (CAS RN)	Activity	Effect	Target
**112**	Euphorbia factor $L_{25.}$ EFL25. Epoxyboetirane M. 5, 15-di-*O*-acetyl-3-benzoyl-6,17-epoxylathyrol. (303174-98-3).			
**S43**	15-acetoxy-3-phenylacetoxy-6,17-epoxy-5-hidroxylathyran-12-en-14-one.	MDR reversal ability	Inhibition of the efflux-pump activity.[18]	P-gp
**120**	5,15-diacetoxy-3-phenylacetoxy-6,17-epoxylathyran-12-en-14-one.			
**S44**	5,15-diacetoxy-3-nicotinoyloxy-6,17-epoxylathyran-12-en-14-one.			
**21**	Epoxiboetirane K. 3β,5α-dibiphenyl-carbonyloxy-15β–hydroxy-6,17-epoxylathyra-12*E*-en-14-one. (1809418-74-3).	MDR reversal ability.	- Modulation of ABCB1-mediated multidrug resistance in Hyman colon adenocarcinoma and mouse T-lymphoma cells. [34]<br>- Synergistic interaction with doxorubicin.[34]<br>- Collateral sensitive effect.[27]	P-gp.
**22**	Epoxiboetirane L. 3β,5α-dinaphtyl-carbonyloxy-15β–hydroxy-6,17-epoxylathyra-12*E*-en-14-one. (1809418-75-4).			
**23**	Epoxiboetirane M. 3β,5α-di-*m*-methylbenzyloxy-15β–hydroxy-6,17-epoxylathyra-12*E*-en-14-one. (1809418-76-5).			
**24**	Epoxyboetirane N. 3β,5α-di(*m*-methoxybenzoylxy)-15β–hydroxy-6,17-			

[34] Matos, A.M.; Reis, M.; Duarte, N.; Spengler, G.; Molnár, J.; Ferreira, M.J.U. Epoxylathyrol Derivatives: Modulation of ABCB1-Mediated Multidrug Resistance in Human Colon Adenocarcinoma and Mouse T-Lymphoma Cells. *J. Nat. Prod.* **2015**, *78*, 2215–2228, doi:10.1021/acs.jnatprod.5b00370.

Comp.	Name(s); (CAS RN)	Activity	Effect	Target
	epoxylathyra-12*E*-en-14-one. (1809418-77-6).			
**25**	Epoxyboetirane O. 3β,5α-di-(*p*-trifluoromethylbenzoylxy)-15β–hydroxy-6,17-epoxylathyra-12*E*-en-14-one. (1809418-78-7).			
**26**	Epoxyboetirane P. 3β,5α-di-(*p*-dimethyaminobenzoylxy)-15β–hydroxy-6,17- epoxylathyra-12*E*-en-14-one. (1809418-79-8).			
**27**	Epoxyboetirane Q. 3β,5α-di-(furan-2-carbonyloxy*)*-15β–hydroxy-6,17-epoxylathyra-12*E*-en-14-one. (1809418-80-1).			
**29**	Epoxycarbamoylboetirane B. (1809418-86-7).			
**30**	Epoxycarbamoylboetirane C. (1809418-87-8).			
**28**	Epoxyboetirane R. 3β,5α- di-(thiophene-2-carbonyloxy*)*--15β–hydroxy-6,17-epoxylathyra-12*E*-en-14-one. (1809418-81-2).	MDR reversal ability.[34]	- Modulation of ABCB1-mediated multidrug resistance in Human colon adenocarcinoma and mouse T-lymphoma cells.<br>-Synergistic interaction with doxorubicin.<br>-Stimulation of the basal ABCB1 ATPase acivity.	P-gp.
**S45**	Epoxyboetirane S. 3β,5α- bis(1-adamantenecarboxilate)-15β–hydroxy-6,17- epoxylathyra-12*E*-en-14-one. (1809418-82-3).		- Modulation of ABCB1-mediated multidrug resistance in human colon adenocarcinoma and mouse T-lymphoma cells.<br>-Synergistic interaction with doxorubicin.	

Comp.	Name(s); (CAS RN)	Activity	Effect	Target
**S46**	Aellinane.<br>2β,5α-Diacetyl-3β,15β-dinicotinyl-14-oxolathyra-12(13*E*)-ene-6(17)-epoxide.	Apoptosis inductor.	-Induces apoptosis in ovarian cancer cell lines.[35]	Mitochondrial pathway.<br>Induction of caspase-6.

[35] Nabatchian, F.; Moradi, A.; Aghaei, M.; Ghanadian, M.; Jafari, S.M.; Tabesh, S. New 6(17)-epoxylathyrane diterpene: aellinane from *Euphorbia aellenii* induces apoptosis via mitochondrial pathway in ovarian cancer cell line. *Toxicol. Mech. Methods* **2017**, *27*, 622–630, doi:10.1080/15376516.2017.1347735.

Section S6. Bioactive isolathyrol (**S47**) derivatives.

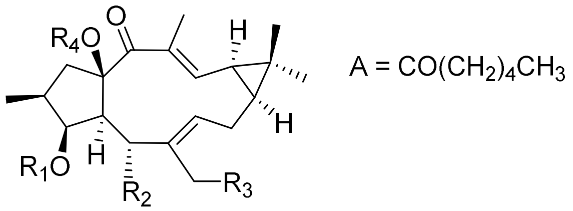

75, 104, 111, 123, 152, 161-3, S(47-50)

Compound	R1	R2	R3	R4	CAS RN	Common name
**75**	Bz	OAc	OAc	Ac	93550-95-9	Euphorbia factor L_{7b}
**104**	Bz	OAc	OAc	Ac	2170110-68-4	Euphorbia factor L713283
**111**	Bz	OAc	H	Ac	1613700-13-2	Euphorbia factor L_{24}
**122**	Bz	OAc	OAc	H	2351821-78-6	-
**151**	Ac	OAc	OAc	H	2750897-07-3	Euphlathyrinoid E
**160**	Ac	OH	H	Bz	1303589-64-1	-
**161**	H	OAc	H	Bz	1303589-67-4	-
**162**	H	OH	H	Bz	158782-02-6	-
**S47**	H	H	H	H	88202-35-1	Isolathyrol
**S48**	Nic	OAc	OAc	Ac	-	-
**S49**	OH	H	OAc	Ac	-	-
**S50**	A	H	OH	Ac	-	-

Ac = acetyl; Bz = benzoyl; Nic = nicotinoyl; OAc = acetoxy.

Table S6. Bioactive isolathyrol (**S47**) derivatives: biological activities, observed effects and molecular targets (where available).

Comp.	Name(s); (CAS RN)	Activity	Effect	Target
**75**	Euphorbia factor L_{7b}. EFL$_{7b}$. 5,15,17-tri-O-acetyl-3- O-benzoyl-5,17-dihydroxyisolathyrol. (93550-95-9).	Anti-inflammatory.	Inhibition of NO production.[3,6]	
		MDR modulator.	Inhibition the efflux-pump activity.[7]	P-gp
**104**	Euphorbia factor L713283. EFL713283. (2170110-68-4).	Anticancer.	Bind to β-tubulin favoring the formation of αβ-tubulin dimmer.[36]	β-Tubulin.
**111**	Euphorbia factor L_{23}. EFL$_{24}$. 15-*O*-acetyl-3-*O*-benzoyl-5,17-dihydroxyisolathyrol. (1613700-13-2).	Anti-inflammatory.	Inhibition of NO production.[6]	
**122**	(2*S*,3*S*,4*R*,5*R*,9*S*,11*R*,15*R*)-3-benzoyloxy-5,17-diacetoxy-15-hydroxy-14-oxolathyra-6*E*,12*E*-diene. (2351821-78-6).	Anti-inflammatory.	Inhibition of NO production.[5]	RAW 264.7
**151**	Euphlathyrinoid E. 3,5,15-Tri-*O*-acetylisolathyrol (2750897-07-3)	Anti-cholestasis.	Activation of Phase I, Phase I metabolism and phase III efflux transporters.[13]	Pregnane X receptor (PXR).
**160**	3-*O*-Acetyl-15-*O*-benzoylisolathyrol. (1303589-64-1).	Vascular-relaxing	Vascular-relaxing activities against phenylephrine (PE)-induced vasoconstriction.[37]	
**161**	5-*O*-Acetyl-15-*O*-benzoylisolathyrol. (1303589-67-4).			
**162**	15-*O*-benzoylisolathyrol. (158782-02-6).			
		Cytotoxic.	Cytotoxicity to human oral epidermoid carcinoma (KB) and its navelbine-selected ABCB1	

[36] Chang, S.; He, H. Q.; Kong, R.; Xie, Z. J.; Hu, J. P. Study on Molecular Recognition between Euphorbia Factor L713283 and β -Tubulin via Molecular Simulation Methods. *J. Chem.* **2015**, *2015*. Article id: 879238; 13 pp, doi: 10.1155/2015/879238.

[37] Tian, Y.; Xu, W.; Zhu, C.; Lin, S.; Li, Y.; Xiong, L.; Wang, S.; Wang, L.; Yang, Y.; Guo, Y.; et al. Lathyrane Diterpenoids from the Roots of *Euphorbia Micractina* and Their Biological Activities. *J. Nat. Prod.* **2011**, *74* (5), 1221–1229; doi: 10.1021/np2001489.

Comp.	Name(s); (CAS RN)	Activity	Effect	Target
			overexpressiing (KBv200) cell lines.[38]	
**152**	Euphlathyrinoid E. 3,5,15-Tri-*O*-acetylisolathyrol (2750897-07-3)	Anti-cholestasis.	Activation of Phase I, Phase I metabolism and phase III efflux transporters.[13]	Pregnane X receptor (PXR).
**S48**	5,15,17-triacetoxy-3 -nicotinoyloxylathyra-6,12-dien-14-one	MDR reversal ability.	Inhibition of the efflux-pump activity.[18]	P-gp.
**S49**	15,17-diacetoxy-3-hydroxylathyra-5, 12-dien-14-one			
**S50**	15,17-diacetoxy-3-hexanoyloxylathyra-5, 12-dien-14-one			

[38] Gao, J.; Chen, Q. Bin; Liu, Y. Q.; Xin, X. L.; Yili, A.; Aisa, H. A. Diterpenoid Constituents of *Euphorbia Macrorrhiza*. *Phytochemistry* **2016**, *122*, 246–253; doi: 10.1016/j.phytochem.2015.12.003.

Section S7. Bioactive jolkinol (**S62**) derivatives.

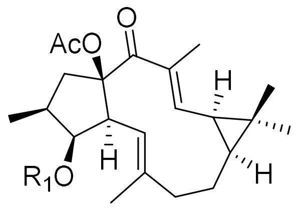

100, S(51-61)

Compound	R1	CAS RN	Common name
**S51**	Ac	1303589-52-7	Jolkinoato A
**S52**	Pr	1422351-40-3	Jolkinoate B
**S53**	Bu	1422351-43-6	Jolkinoate C
**S54**	Dodec	1422351-44-7	Jolkinoate D
**S55**	2-MePen	1422351-45-8	Jolkinoate E
**S56**	2-EtBu	1422351-46-9	Jolkinoate F
**S57**	2-EtHex	1422351-47-0	Jolkinoate G
**100**	Bz	1422351-48-1	Jolkinoate I
**S58**	*p*-MeBz	1422351-49-2	Jolkinoate J
**S59**	*p*-MeOBz	1422351-50-5	Jolkinoate K
**S60**	*m*-CF_3Bz	1422351-51-6	Jolkinoate L
**S61**	$PhSO_2$	1422351-52-7	Jolkinoate M

Ac = acetyl; Bu = butanoyl; Bz = benzoyl; Dodec = dodecanoyl; 2-EtBu = 2-ethylbutyl; 2-EtHe = 2-ethylhexanoyl; *p*-MeBz = *p*-methylbenzoyl; *p*-MeOBz = *p*-methoxybenzoyl; 2-MePen = 2-methylpentanoyl; *m*-CF_3Bz = *m*-trifluoromethylbenzoyl; $PhSO_2$ = phenylsulfone; Pr = propanoyl.

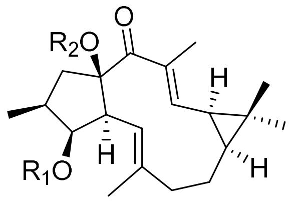

31-42, 143, 160, S62-S65

Compound	R_1	R_2	CAS RN	Common name
**S62**	H	H	62820-24-0	Jolkinol
**31**	H	Ac	62820-14-8	Jolkinol D
**32**	*m*-MeOBz	H	2093947-05-6	Jolkinolate A
**33**	*p*-CF_3Bz	H	2093947-06-7	Jolkinolate B
**34**	Naph	H	2093947-07-8	Jolkinolate C
**35**	BiPh	H	2093947-08-9	Jolkinolate D
**36**	*m*-MeBz	Ac	2093946-95-1	Jolkinoate N
**37**	*m*-MeOBz	Ac	2093946-96-2	Jolkinoate O
**38**	o-MeOBz	Ac	2093946-97-3	Jolkinoate P
**39**	*p*-CF_3Bz	Ac	2093946-98-4	Jolkinoate Q
**40**	Cin	Ac	2093946-99-5	Jolkinoate R
**41**	Naph	Ac	2093947-00-1	Jolkinoate S
**42**	BiPh	Ac	2093947-01-2	Jolkinoate T
**142**	H	Cin	1303589-56-1	-
**159**	H	Bz	1303589-58-3	-
**S63**	Adam	Ac	2093947-02-3	Jolkinoate U
**S64**	EtOCO	Ac	2093947-03-4	jolkinocarbonate A
**S65**	PhOCO	Ac	2093947-04-05	jolkinocarbonate B

Ac = acetyl; Adam = 1-adamantanecarbonyl; BiPh= p-phenylbenzoyl; Bz = benzoyl; *p*-CF_3Bz = *p*-trifluoromethylbenzoyl; Cin = cinnamoyl; EtOCO = ethoxycarbonyl; *m*-MeBz = *m*-methylbenzoyl; *m*-MeOBz = *m*-methoxybenzoyl; *o*-MeOBz = *o*-methoxybenzoyl; Naph= 2-naphthoyl; PhOCO = phenoxycarbonyl.

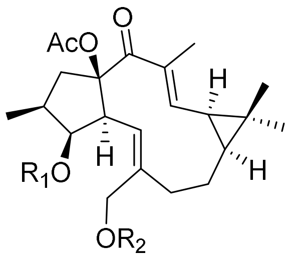

74, 106, S66

Compound	R_1	R2	CAS RN	Common name
**74**	cinnamoyl	Ac	93550-94-8	Euphorbia factor L_{7a}
**106**	H	Ac	2269487-20-7	Euphorbia factor L_{29}
**S66**	$CO(CH_2)_4CH_3$	H	496776-71-7	Euphorbia factor L_{10}

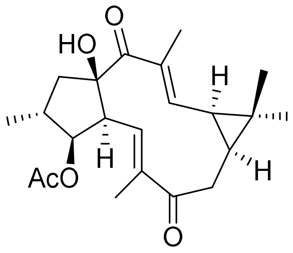

Jatropodagin A (**96**)

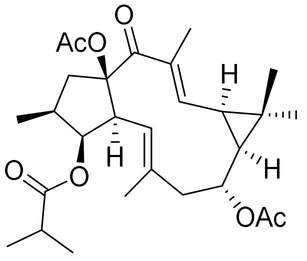

ELAF12-2 (**145**)

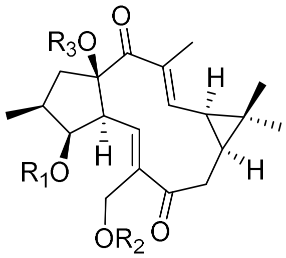

R_1=Bz, R_2=H, R_3=Ac,
Euphlathyrinoid A (**148**)
R_1=H, R_2=Bz, R_3=H,
Euphlathyrinoid B (**149**)

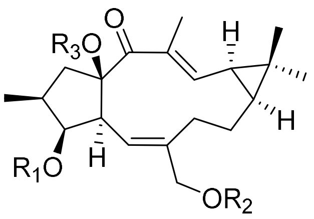

108-110, 118, 127-128, S67

Compound	R_1	R_2	R_3	CAS RN	Common name
**108**	Bz	Ac	Ac	1613699-99-2	Euphorbia factor L_{17}
**109**	Cin	H	Ac	1613700-09-6	Euphorbia factor L_{22}
**110**	n-Hex	Ac	Ac	1613700-11-0	Euphorbia factor L_{23}
**117**	n-Hex	H	Ac		-
**126**	Nic	Ac	Ac	2677056-94-7	Euplarisan A
**127**	Bz	H	Ac	2677056-95-8	Euplarisan B
**S67**	PhAc	Ac	Ac	1613700-01-8	Euphorbia factor L_{18}

Ac = acetyl; Bz = benzoyl; Cin = cinnamoyl; n-Hex = hexanoyl; PhAc = phenylacetoyl; Nic = nicotinoyl.

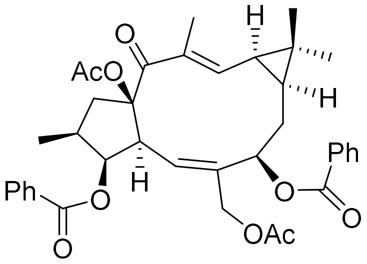

Euplarisan D (**128**)
(CAS RN: 2677056-97-0)

Table S7. Bioactive jolkinol (**S62**) derivatives: biological activities, observed effects and molecular targets (where available).

**Comp.**	**Name(s); (CAS RN)**	**Activity**	**Effect**	**Target**
**31**	Jolkinol D. (62820-14-8).	MDR reversal ability.	Antiproliferative in L5178Y mouse lymphoma cells (PAR cells) and in human MDR REVERSAL ABILITY1-gene transfected L5178Y mouse lymphoma cells (MDR REVERSAL ABILITY cells).[39,40]	P-gp.
**S51**	Jolkinoate A. (1303589-52-7).			
**S52**	Jolkinoate B. (1422351-40-3).			
**S53**	Jolkinoate C. (1422351-43-6).			
**S54**	Jolkinoate D. (1422351-44-7).			
**S55**	Jolkinoate E. (1422351-45-8).			
**S56**	Jolkinoate F. (1422351-46-9).			
**S57**	Jolkinoate G. (1422351-47-0).			
**100**	Jolkinoate I. (1422351-48-1).		Inhibition of Rhodamine-123 Efflux P-gp-Mediated.[39,40]	
**S58**	Jolkinoate J. (1422351-49-2).			
**S59**	Jolkinoate K. (1422351-50-5).			
**S60**	Jolkinoate L. (1422351-51-6).	Apoptosis induction	Induction of apoptosis in human gastric cancer cell lines EPG85-257P and EPG85-257RNOV.[41]	Caspase-3 activation.
		MDR reversal ability	Inhibition of Rhodamine-123 Efflux P-gp-Mediated.[39,40]	P-gp.
**S61**	Jolkinoate M. (1422351-52-7).			

[39] Reis, M.; Ferreira, R. J.; Santos, M. M. M.; Dos Santos, D. J. V. A.; Molnár, J.; Ferreira, M. J. U. Enhancing Macrocyclic Diterpenes as Multidrug-Resistance Reversers: Structure-Activity Studies on Jolkinol D Derivatives. *J. Med. Chem.* **2013**, *56* (3), 748–760; doi: 10.1021/jm301441w.

[40] Ferreira, M. J. U.; Duarte, N.; Reis, M.; Madureira, A. M.; Molnár, J. *Euphorbia* and *Momordica* Metabolites for Overcoming Multidrug Resistance. *Phytochem. Rev.* **2014**, *13* (4), 915–935; doi: 10.1007/s11101-014-9342-8.

[41] Reis, M. A.; Ahmed, O. B.; Spengler, G.; Molnár, J.; Lage, H.; Ferreira, M. J. U. Exploring Jolkinol D Derivatives to Overcome Multidrug Resistance in Cancer. *J. Nat. Prod.* **2017**, *80* (5), 1411–1420; doi: 10.1021/acs.jnatprod.6b01084.

Comp.	Name(s); (CAS RN)	Activity	Effect	Target
**S62**	Jolkinol.<br>Jolkinodiol.<br>(62820-24-0).			
**32**	Jolkinolate A.<br>(2093947-05-6).	MDR reversal ability	Impairment of ABCB1 transporter P-glycoprotein efflux.[41]	P-gp.
**33**	Jolkinolate B.<br>(2093947-06-7).			
**34**	Jolkinolate C.<br>(2093947-07-8).			
**35**	Jolkinolate D.<br>(2093947-08-9).			
**36**	Jolkinoate N.<br>(2093946-95-1).	MDR reversal ability	Impairment of ABCB1 transporter P-glycoprotein efflux.[41]	P-gp.
**37**	Jolkinoate O.<br>(2093946-96-2).			
**38**	Jolkinoate P.<br>(2093946-97-3).			
**39**	Jolkinoate Q.<br>(2093946-98-4).			
**40**	Jolkinoate R.<br>(2093946-99-5).			
**41**	Jolkinoate S.<br>(2093947-00-1).			
**42**	Jolkinoate T.<br>(209397-01-2).			
**S63**	Jolkinoate U.<br>(2093947-02-3).			
		Apoptosis induction	Induction of apoptosis in human gastric cancer cell line EPG85-257RNOV.[41]	Caspase-3 activation.
**142**	15-*O*-Cinammoyljolkinol.<br>(1303589-56-1).	Anti-viral.	Inhibition of HIV-1 replication.[37]	
**159**	15-*O*-Benzoyljolkinol.<br>(1303589-58-3).	Vascular-relaxing.	Vascular-relaxing activities against phenylephrine (PE)-	

Comp.	Name(s); (CAS RN)	Activity	Effect	Target
			induced vasoconstriction.[37]	
**S64**	Jolkinocarbonate A. (2093947-03-4).	MDR reversal ability	Impairment of ABCB1 transporter P-glycoprotein efflux.[41]	P-gp.
**S65**	Jolkinocarbonate B. (2093947-04-5).			
		Apoptosis induction	Induction of apoptosis in human pancreatic cancer cell lines EPP85-181RNOV and EPP85-181RDB.[41]	Caspase-3 activation.
**74**	Euphorbia factor L_{7a}. EFL7a. 15,17- di-O-acetyl-3-O-cinnamyl-17-hydroxyjolkinol. (93550-94-8).	Anti-inflammatory.	Inhibition of NO production in LPS-induced RAW 264.7 macrophages.[6,42]	
		MDR modulator.	Modulator of multidrug resistance on human breast cancer MCF-7/ADM cell line (Inhibition the efflux-pump activity).[23]	P-gp.
**106**	Euphorbia factor L_{29}. EFL29. (2269487-20-7).	Anti-inflammatory.	Inhibition of NO production.[6]	
**S66**	Euphorbia factor L_{10}. EFL10. (5*E*)-15-*O*-acetyl-3-*O*-hexanoyl-17-hydroxyjolkinol. (496776-71-7).	MDR reversal ability.	P-gp Inhibition.[43]	P-gp
		Anti-inflammatory.	Inhibition of NO production.[5]	
**96**	Jatropodagin A.	Cytotoxic.[44]	- Cytotoxic against human osteosarcoma cell lines MG-63 and Saos-2.<br>Apoptosis Induction.	

[42] Wang, Y.; Song, Z.; Guo, Y.; Xie, H.; Zhang, Z.; Sun, D.; Li, H.; Chen, L. Diterpenoids from the Seeds of *Euphorbia Lathyris* and Their Anti-Inflammatory Activity. *Bioorg. Chem.* **2021**, *112*, 104944; doi: 10.1016/j.bioorg.2021.104944.

[43] Appendino, G.; Porta, C. Della; Conseil, G.; Sterner, O.; Mercalli, E.; Dumontet, C.; Di Pietro, A. A New P-Glycoprotein Inhibitor from the Caper Spurge (*Euphorbia Lathyris*). *J. Nat. Prod.* **2003**, *66* (1), 140–142; doi: 10.1021/np0203537.

[44] Yuan, H. T.; Li, Q. F.; Tian, T.; Zhang, C. Y.; Huang, Z. Q.; Fan, C. X.; Mei, K.; Zhou, J.; Zhai, X. X.; Li, S. B.; et al. Lathyrane Diterpenoids from *Jatropha Podagrica* and Their Antitumor Activities in Human Osteosarcoma Cells. *Nat. Prod. Res.* **2020**, *35* (23), 5089–5095; doi: 10.1080/14786419.2020.1779719.

Comp.	Name(s); (CAS RN)	Activity	Effect	Target
**145**	ELAF12-2 (2S,3S,4S,8R,9S,11R,15R)-8,15-diacetoxy-3-isobutyroxy-14-oxolathyra-(5E),(12E)-diene.	NPC proliferation.[73]		PKC β
**148**	Euphlathyrinoid A. 15-*O*-acetyl-3-*O*-benzoyl-17-hydroxyjolkinol (2750896-73-0).	Anti-cholestasis.	Activation of Phase I, Phase I metabolism and phase III efflux transporters.[13]	Pregnane X receptor (PXR).
**149**	Euphlathyrinoid B. 15-*O*-benzoyl-17-hydroxyjolkinol (2750896-90-1).			
**108**	Euphorbia factor L_{17}. EFL17. (5*E*)-15,17-Di-*O*-acetyl-3-*O*-benzoyl-17-hydroxyjolkinol. (1613699-99-2).	Anti-inflammatory.	Inhibitory effect on NO production.[6,42]	
**109**	Euphorbia factor L_{22}. EFL22. (5*E*)-15-*O*-acetyl-3-*O*-cinnamyl-17-hydroxyjolkinol. (1613700-09-6).	Anti-inflammatory.	Inhibition of NO production.[5,6]	
**110**	Euphorbia factor L_{23}. EFL23. 15,17-di-*O*-acetyl-3-*O*-hexanoyl-17-hydroxyjolkinol. (1613700-11-0).	Cytotoxic.	Cytotoxic activity against MCF-7 cancer cell line (SAR).[15,14]	
		Anti-inflammatory.	Inhibition of NO production.[6]	
**117**	15-*O*-Acetyl-3-*O*-hexanoyl-17-hydroxyjolkinol. (2415155-50-7).	Anti-inflammatory.	Inhibition of NO production.[6]	
**126**	Euplarisan A. (5*E*)-15,17-di-*O*-acetyl-3-*O*-nicotinoyl-17-hydroxyjolkinol. (2677056-94-7).	Anti-inflammatory.[42]	- Inhibitory effect on NO production in LPS-induced RAW264.7 macrophage cells.<br>- Inhibition of inflammatory cytokines (IL-1β, IL-6 and TNF-α)).	iNOS, COX-2 and p-IκBα.
**127**	Euplarisan B. (5*E*)-15-*O*-acetyl-3-*O*-benzoyl-17-hydroxyjolkinol.	Anti-inflammatory.[42]	Inhibitory effect on NO production in LPS-induced RAW264.7 macrophage cells.	

Comp.	Name(s); (CAS RN)	Activity	Effect	Target
	(2677056-95-8).			
**S67**	Euphorbia factor L_{18}. EFL18. 15,17-di- O-acetyl-3-O-phenylacetyl-17-hydroxyjolkinol. (1613700-01-8).	Cytotoxic.	Cytotoxic activity against MCF-7 cancer cell line (SAR).[15]	
**128**	Euplarisan D. (5*E*,7*R*)-15,17-di-*O*-acetyl-3,7-di-*O*-benzoyl-7,17-dihydroxyjolkinol. (2677056-97-0).	Anti-inflammatory.	Inhibitory effect on NO production in LPS-induced RAW264.7 macrophage cells.[42]	

Section S8. Bioactive 15-deacyl jolkinol B (**S68**) derivatives.

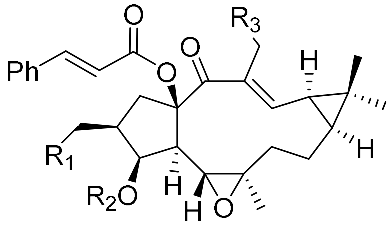

1-5, 7-9, 83, 89

Compound	R1	R2	R3	CAS RN	Common name
**1**	OAc	H	H	882041-54-5	Latilagascene A
**2**	OH	H	H	882041-55-6	Latilagascene B
**3**	OAc	Ac	H	882041-56-7	Latilagascene C
**4**	OBz	H	H	919350-68-8	Latilagascene D
**5**	OBz	H	OH	919521-87-2	Latilagascene E
**7**	OBz	Ac	H	1089146-74-6	Latilagascene G
**8**	OPr	Pr	H	1089146-75-7	Latilagascene H
**9**	OBu	*n*-Bu	H	1089146-76-8	Latilagascene I
**83**	H	H	H	62820-12-6	Jolkinol B
**89**	H	H	OH	62820-11-5	Jolkinol A

Ac= acetyl; *n*-Bu = *n*-butyryl; OAc = Acetate; OBu = butanoate; OBz = benzoate; Pr = propionyl.

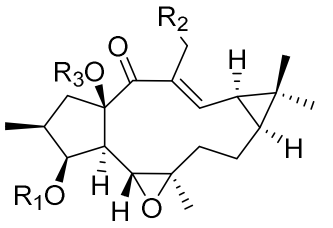

6, 87-88, 90, 99, 113, 124, 159, S68-S69

Compound	R_1	R_2	R_3	CAS RN	Common name
**6**	H	H	Bz	919521-89-4	Latilagascene F
**87**	H	H	*p*-OHCin	-	Euphofischer A
**88**	H	OAc	Cin	-	Euphofischer B
**90**	H	OH	H	2247683-39-0	Ebracteolata C
**99**	Ac	H	Bz	721940-31-4	-
**93**	Nic	H	Ac	88202-64-6	Euphorbia factor L_{26}
**123**	Bz	H	Ac	88202-63-5	Euphorbia factor L_{12}
**158**	Bz	H	H	1303589-49-2	-
**S68**	H	H	H	62820-26-2	15-Deacyl Jolkinol B
**S69**	H	H	Bz	1648739-94-9	Macrorilathyrone B

Ac = acetyl; Bz = benzoyl; Cin = cinnamoyl; *p*-OHCin = *p*-hydroxycinnamoyl; Nic = nicotinoyl.

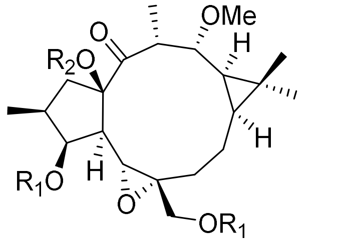

Methoxyboetirane A (R_1=R_2=Ac, **84**).
(CAS RN: 1809418-88-9)
Methoxyboetirane B (R_1=Bz, R_2=H, **85**).
(CAS RN: 1809418-89-0)
Methoxyboetirane C (R_1=*p*-CF_3PhCO, R_2=H **86**).
(CAS RN: 1809418-90-3)

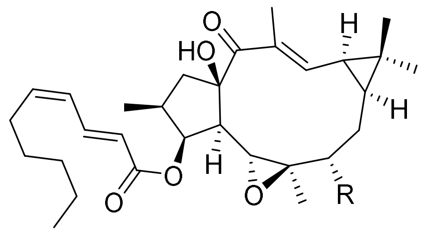

Euphstrachenol A (R=H, **97**)
Euphstrachenol B (R=OAc, **98**)

Table S8. Bioactive 15-deacyl jolkinol B (**S68**) derivatives: biological activities, observed effects and molecular targets (where available).

Comp.	Name(s); (CAS RN)	Activity	Effect	Target
**1**	Latilagascene A. (882041-54-5).	Anticancer.	MDR reversal ability inhibition.[45]	
		Apoptosis inductor.[47]		
**2**	Latilagascene B. (882041-55-6).	Anticancer.	Antiproliferative.[47]	
		MDR reversal ability	Modulation of P-glycoprotein.[47,45]	P-gp.
		Apoptosis inductor.[47]		
		Antitumor.	Over-expression MDR reversal ability.[48]	
**3**	Latilagascene C. (882041-56-7).	Anticancer.	MDR reversal ability.[45]	
			Apoptosis inductor.[47]	
		Antitumor.	Over-expression MDR reversal ability.[48]	P-gp.
			Anti-neoplastic activity.[46]	
**4**	Latilagascene D. (919350-68-8).	MDR reversal ability.	Inhibition of the efflux-pump activity.[47]	P-gp.
			MDR reversal ability human colon adenocarcinoma cells (COLO 320).[40]	
		Anticancer.	Apoptosis inductor.[47]	
		Antitumor.	Over-expression MDR reversal ability.[48]	P-gp.
			Antineoplastic activity.[46]	

[45] Duarte, N.; Gyémánt, N.; Abreu, P. M.; Molnár, J.; Ferreira, M. J. U. New Macrocyclic Lathyrane Diterpenes, from *Euphorbia Lagascae*, as Inhibitors of Multidrug Resistance of Tumour Cells. *Planta Med.* **2006**, *72* (2), 162–168; doi: 10.1055/s-2005-873196.
[46] Vasas, A.; Hohmann, J. *Euphorbia* Diterpenes: Isolation, Structure, Biological Activity, and Synthesis (2008-2012). *Chem. Rev.* **2014**, *114* (17), 8579–8612; doi: 10.1021/cr400541j.

Comp.	Name(s); (CAS RN)	Activity	Effect	Target
**5**	Latilagascene E. (919521-87-2).	MDR reversal ability.	Inhibition of the efflux-pump activity.[47]	P-gp.
**6**	Latilagascene F. (919521-89-4).			
**7**	Latilagascene G. (1089146-74-6).			
**8**	Latilagascene H. (1089146-75-7).	MDR reversal ability.	MDR reversal ability human colon adenocarcinoma cells (COLO 320).[40]	P-gp.
**9**	Latilagascene I. (1089146-76-8).			
**83**	Jolkinol B. EM-E-11-4. (62820-12-6).	MDR reversal ability.	Inhibition of the efflux-pump activity.[16,47]	P-gp.
		Apoptosis Inductor.[47]		
		Antitumoral/cytotoxic	Cytotoxic against multidrug resistant colon carcinoma variant HT-29RNOV.[48]	
			Accumulated human cytomegalovirus (CMV) immediate-early (IE) antigen expression decreases in lung cancer cells (A549) infected by CMV.[49]	
			Cytotoxicity against MV4-11 (leukemia), H460 (lung cancer) and Skvo3 (ovarian cancer) cell lines.[55]	
		Anti-MDR	Activity in paclitaxel-resistant tumor cells overexpressing either P-gp or βIII tubulin.[50]	P-gp and βIII tubulin.
**89**	Jolkinol A. (62820-11-5)	Anti-inflammatory	inhibitory effects on LPS-induced NO production in RAW	

[47] Duarte, N.; Varga, A.; Cherepnev, G.; Radics, R.; Molnár, J.; Ferreira, M. J. U. Apoptosis Induction and Modulation of P-Glycoprotein Mediated Multidrug Resistance by New Macrocyclic Lathyrane-Type Diterpenoids. *Bioorganic Med. Chem.* **2007**, *15* (1), 546–554; doi: 10.1016/j.bmc.2006.09.028.
[48] Lage, H.; Duarte, N.; Coburger, C.; Hilgeroth, A.; Ferreira, M. J. U. Antitumor Activity of Terpenoids against Classical and Atypical Multidrug Resistant Cancer Cells. *Phytomedicine* **2010**, *17* (6), 441–448; doi: 10.1016/j.phymed.2009.07.009.
[49] Pusztai, R.; Ferreira, M. J. U.; Duarte, N.; Engi, H.; Molnar, J. Macrocyclic Lathyrane Diterpenes as Antitumor Promoters. *Anticancer Res.* **2007**, *27* (1 A), 201–206 (PubMed ID: 17352233).
[50] Liu, Q.; Cai, P.; Guo, S.; Shi, J.; Sun, H. Identification of a Lathyrane-Type Diterpenoid EM-E-11-4 as a Novel Paclitaxel Resistance Reversing Agent with Multiple Mechanisms of Action. *Aging (Albany. NY).* **2020**, *12* (4), 3713–3729; doi: 10.18632/aging.102842.

Comp.	Name(s); (CAS RN)	Activity	Effect	Target
			264.7 macrophage cells.[6,51]	
		Cytotoxic/antitumoral	Inhition on mammosphere formation in human breast cancer MCF-7 cells 10 μM dose.[52,53]	
			Moderate dose-dependent growth inhibitory effect against the MCF-7 and NCI-H460 cell lines.[54]	
			Cytotoxicity against H460 (lung cancer) and Skvo3 (ovarian cancer) cell lines.[55]	
		Anti-viral.	Anti-respiratory syncytial virus (RSV).[56]	
**87**	Euphofischer A.	Cytotoxic.	Citoxicity against C4-2B cell line.[57]	
**88**	Euphofischer B.			
**90**	Ebracteolata C. (2247683-39-0).			
**93**	Euphorbia factor L_{26}. (2*S*,3*S*,4*R*,5*R*,6*R*,9*S*,11*R*,15*R*)-15-*O*-Acetyl-5,6-epoxy-3-*O*-nicotinoyljolkinol.	Anti-inflammatory.	Inhibition of NO production.[3,6]	

[51] Lee, J. W.; Lee, C.; Jin, Q.; Jang, H.; Lee, D.; Lee, H.-J.; Shin, J. W.; Han, S. B.; Hong, J. T.; Kim, Y.; et al. Diterpenoids from the Roots of *Euphorbia Fischeriana* with Inhibitory Effects on Nitric Oxide Production. *J. Nat. Prod.* **2016**, *79* (1), 126–131; doi: 10.1021/acs.jnatprod.5b00789.

[52] Jian, B.; Zhang, H.; Liu, J. Structural Diversity and Biological Activities of Diterpenoids Derived from *Euphorbia Fischeriana* Steud. *Molecules* **2018**, *23* (4), 935; doi: 10.3390/molecules23040935.

[53] Kuang, X.; Li, W.; Kanno, Y.; Yamashita, N.; Kikkawa, S.; Azumaya, I.; Nemoto, K.; Asada, Y.; Koike, K. Euphorins A–H: Bioactive Diterpenoids from *Euphorbia Fischeriana*. *J. Nat. Med.* **2016**, *70* (3), 412–422; 10.1007/s11418-016-0987-z.

[54] Valente, C.; Pedro, M.; Ascenso, J. R.; Abreu, P. M.; Nascimento, M. S. J.; Ferreira, M. J. U. Euphopubescenol and Euphopubescene, Two New Jatrophane Polyesters, and Lathyrane-Type Diterpenes from *Euphorbia Pubescens*. *Planta Med.* **2004**, *70* (3), 244–249; doi: 10.1055/s-2004-815542.

[55] Ye, Y.; Liu, G. H.; Dawa, D.; Ding, L. S.; Cao, Z. X.; Zhou, Y. Cytotoxic Diterpenoids from the Roots of *Euphorbia Stracheyi*. *Phytochem. Lett.* **2020**, *36* (December 2019), 183–187; doi: 10.1016/j.phytol.2020.03.002.

[56] Huang, C. S.; Luo, S. H.; Li, Y. L.; Li, C. H.; Hua, J.; Liu, Y.; Jing, S. X.; Wang, Y.; Yang, M. J.; Li, S. H. Antifeedant and Antiviral Diterpenoids from the Fresh Roots of *Euphorbia Jolkinii*. *Nat. Products Bioprospect.* **2014**, *4* (2), 91–100; doi: 10.1007/s13659-014-0009-3.

[57] Li, J.; He, J.; Yang, C.; Yan, X.; Yin, Z. Cytotoxic Lathyrane Diterpenoids from the Roots of *Euphorbia Fischeriana*. *Rec. Nat. Prod.* **2020**, *14* (4), 286–291; doi: 10.25135/rnp.167.19.12.1496.

Comp.	Name(s); (CAS RN)	Activity	Effect	Target
	(88202-64-6).[58]			
**123**	Euphorbia factor L_{12}. EFL_{12}. (2*S*,3*S*,4*R*,5*R**,6*R**,9*S*,11*R*,15*R*)-15-*O*-acetyl-3-*O*-benzoyljolkinol-5,6-oxide. (88202-63-5).	Cytotoxic.	Cytotoxic activity against MCF-7 cancer cell line (SAR).[14,15]	
**99**	(-)-(12*E*,2S,3*S*,4*R*,5R,6R,9*S*, 11*S*,15*R*)-3-Acetoxy-15-benzoyloxy-5,6-epoxy lathyr-12-en-14-one. (721940-31-4).	MDR reversal ability.	Inhibition of the efflux-pump activity.[16]	P-gp
		Vascular-relaxing.	Vascular-relaxing activities against phenylephrine (PE)-induced vasoconstriction.[37]	
**158**	(-)-(12*E*,2S,3*S*,4*R*,5R,6R,9*S*, 11*S*,15*R*)-3-benzoyloxy-5,6-epoxylathyr-12-en-14-one. (1303589-49-2).			
**S69**	Macrorilathyrone B. (1648739-94-9).	Cytotoxic.	Cytotoxicity to human oral epidermoid carcinoma (KB) and navelbine-selected ABCB1 overexpressiing (KBv200) cell lines.[38]	
		MDR reversal ability.	Inhibition of the efflux-pump activity.[38]	P-gp.
**84**	Methoxyboetirane A. (1809418-88-9).	MDR reversal ability.	- ABCB1 inhibitory activity and synergistic interaction with doxorubicin.[34] - Collateral sensitive effect and apoptosis via caspase-3 activation.[27]	
**85**	Methoxyboetirane B. (1809418-89-0).			
**86**	Methoxyboetirane C (1809418-90-3).			
**97**	Euphstrachenol A	Cytotoxic	Moderate cytotoxicity against MV4-11 cell line.[55]	
**98**	Euphstrachenol B			

58 Wrongly assigned as (2*S*,3*S*,4*R*,5*S*,6*R*,9*S*,11*R*,15*R*)-15-*O*-Acetyl-5,6-epoxy-3-*O*-nicotinoyljolkinol (2268691-23-0).

Section S9. Bioactive laurifolioside (**105**) derivatives.

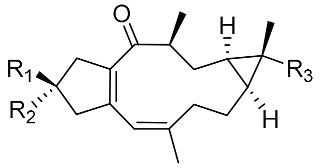

Laurifolioside (**105**)
R_1= CH_3, R_2= H; R_3= 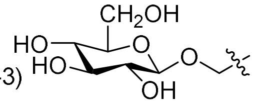

(CAS RN: 2015239-75-3)

2-*epi*-Laurifolioside (**S70**)
R_1= H, R_2= CH_3; R_3= 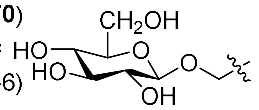

(CAS RN: 2015227-94-6)

Laurifolioside A (**143**)
R_1= CH_3, R_2= H; R_3= 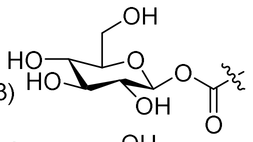

(CAS RN: 2173411-03-3)

2-*epi*-Laurifolioside A (**S71**)
R_1= H, R_2= CH_3; R_3= 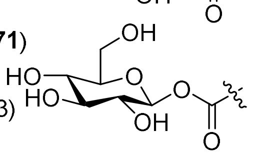

(CAS RN: 2173092-08-3)

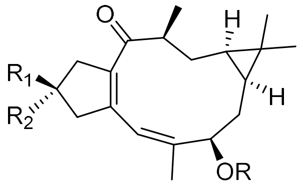

Laurifolioside B (**S72**)
R_1= CH_3, R_2= H; R_3= 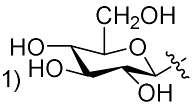

(CAS RN: 2231241-43-1)

2-*epi*-Laurifolioside B (**S73**)
R_1= H, R_2= CH_3; R_3= 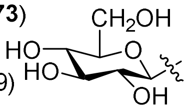

(CAS RN: 2231241-83-9)

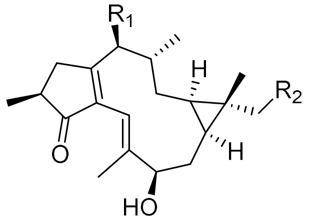

Kansuingol A (**133**)
R_2=OH; R_1= 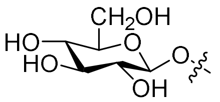

(CAS RN: 2226832-98-2)

Kansuingol B (**134**)
R_1=OH; R_2= 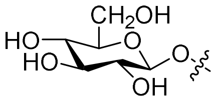

(CAS RN: 2226832-99-9)

Table S9. Bioactive Laurifolioside (**105**) derivatives: biological activities, observed effects and molecular targets (where available).

Comp.	Name(s); (CAS RN)	Activity	Effect	Target
**105**	Laurifolioside. (2015239-75-3)	Modulation of trafficking pathways	Modulation of clathrin activity on human prostrate cancer (PC-3) and human breast adenocarcinoma (MCF-7) cells.[59]	Clatrin heavy chain 1
		Cytotoxic	Cytotoxicity to human prostrate cancer (PC-3) and human breast adenocarcinoma (MCF-7).[59]	
		Anti hepatitis B virus (HBV) activity.[60]	Inhibitory effect on HBV-DNA replication.	
**S70**	2-*epi*-Laurifolioside. (2015227-94-6).			
**143**	Laurifolioside A. (2173411-03-3).		Activity against HBV surface antigen (HBsAg)	
**S71**	2-*epi*-Laurifolioside A. (2173092-08-3).		Inhibitory effect on HBV-DNA replication.	
**S72**	Laurifolioside B. (2231241-43-1).			
**S73**	2-*epi*-Laurifolioside B. (2231241-83-9).			
**133**	Kansuingol A. (2226862-98-2).	Anti-inflammatory.	Inhibitory activity of the production of pro-inflamatory cytokine interleukin 6 (IL-6), in human mast cell line (HMC-1), stimulated by PMA[61] and an ionophore (A23187).[62]	Interleukin 6 (IL-6).
**134**	Kansuingol B. (2226832-99-9).			

[59] Dal Piaz, F.; Vera Saltos, M. B.; Franceschelli, S.; Forte, G.; Marzocco, S.; Tuccinardi, T.; Poli, G.; Nejad Ebrahimi, S.; Hamburger, M.; De Tommasi, N.; et al. Drug Affinity Responsive Target Stability (DARTS) Identifies Laurifolioside as a New Clathrin Heavy Chain Modulator. *J. Nat. Prod.* **2016**, *79* (10), 2681–2692; dxoi: 10.1021/acs.jnatprod.6b00627.

[60] Li, S. F.; Jiao, Y. Y.; Zhang, Z. Q.; Chao, J. Bin; Jia, J.; Shi, X. L.; Zhang, L. W. Diterpenes from Buds of *Wikstroemia Chamaedaphne* Showing Anti-Hepatitis B Virus Activities. *Phytochemistry* **2018**, *151*, 17–25; doi: 10.1016/j.phytochem.2018.01.021.

[61] Phorbol 12-myristate 13-acetate (CAS RN: 16561-29-8).

[62] Kim, Y. M.; Ahn, J.; Chae, H. S.; Choi, Y. H.; Kim, J.; Chin, Y. W. Two New Lathyrane-Type Diterpenoid Glycosides with IL-6 Production Inhibitory Activity from the Roots of *Euphorbia Kansui*. *Bioorganic Med. Chem. Lett.* **2018**, *28* (7), 1207–1210; doi: 10.1016/j.bmcl.2018.02.050.

Section S10. Bioactive jatrogrossidion (**S78**) derivatives.

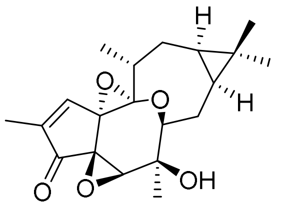

Jatrocurcasenone H (**138**)

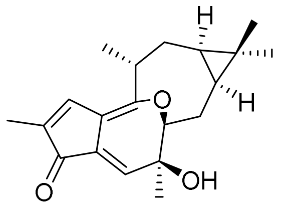

Jatrocurcasenone **I** (**139**)

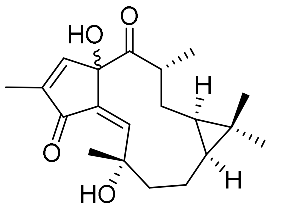

(4*Z*)-Jatrogrossidentadion
(R= β-OH, **154**)
(CAS RN: 143062-69-5)

15-*epi*-(4*Z*)-Jatrogrossidentadion
(R= α-OH, **155**)
(CAS RN: 143062-70-8)

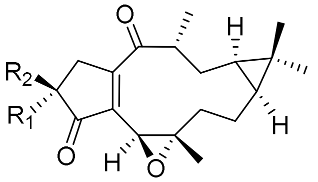

2-Hydroxyisojatrogrossidion
(R_1= OH, R_2= CH_3, **156**)
(CAS RN: 106644-32-0)

2-*epi*-Hydroxyisojatrogrossidion
(R_1= CH_3, R_2= OH, **157**)
(CAS RN: 143120-26-7)

Isojatrogrossidion
(R_1= H, R_2= CH_3, **S74**)
(CAS RN: 143022-03-1)

2-*epi*-Isojatrogrossidion
R_1= CH_3, R_2= H (**S75**)
(CAS RN: 143120-25-6)

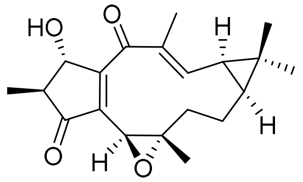

Jatrointelona K (**144**)
(CAS RN: 2225948-95-6)

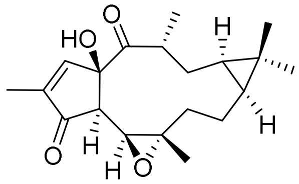

Jatrogrossidion **(S78)**
(CAS RN: 219494-36-7)

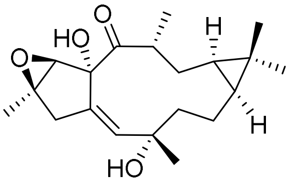

Japodagrin (**153**)
(CAS RN: 956357-91-8)

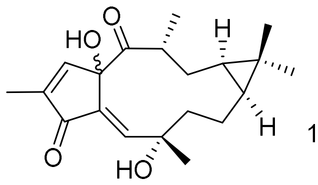

(4*E*)-Jatrogrossidentadion
(R= β-OH, **S76**)
(CAS RN: 143050-04-8)

15-*epi*-(4*E*)-Jatrogrossidentadion
(Japodagrol, R= α-OH, **S77**)
(CAS RN: 143120-24-5)

Table S10. Bioactive Jatrogrossidion (**S78**) derivatives: biological activities, observed effects and molecular targets (where available).

**Comp.**	**Name(s); (CAS RN)**	**Activity**	**Effect**	**Target**
**138**	Jatrocurcasenone H	Anti-inflammatory activity.	Inhibition of NO-production with LPS-stimulated RAW264.7 macrophages.	iNOS and COX-2.[63]
**139**	Jatrocurcasenone I			- iNOS and COX-2.<br>- Ptgs2 (pro-inflammatory enzyme).[63,64]
**144**	Jatrointelona K. (2225948-95-6).	Anti-viral	Anti-chikungunya (CHIKV) virus.[65]	
**153**	Japodagrin. (956357-91-8).	Antibacterial	-Antibacterial against *Bacillus subtillis* (ATCC 6051) and *Staphylococcus aureus* (ATCC 25923).[66]	
**154**	4Z-Jatrogrossidenta-dion. (143062-69-5).	Antibacterial	-Antibacterial against *Bacillus subtillis* (ATCC 6051) and *Staphylococcus aureus* (ATCC 25923).[66]	
		Cytotoxic	Antiproliferative activity against A549, MDAMB231 and HEPG2 and cancer cell lines.[67]	
			Antiproliferative activity against L5178Y mouse lymphoma cell line.[68]	
			Antiproliferative activity against A549 (human non-small-cell lung carcinoma), HL-60 (human myeloid leukemia), SMMC-7721 (hepatocellular carcinoma), MCF-7 (breast cancer) and SW480 (colon cancer) cancer cell lines.[69]	
**155**		Antibacterial	-Antibacterial against *Bacillus subtillis* (ATCC 6051) and	

[63] Huang, J.-D.; Zhang, C.; Xu, W.-J.; Lian, C.-L.; Liu, X.-M.; Wang, C.-F.; Liu, J.-Q. New lathyrane diterpenoids with anti-inflammatory activity isolated from the roots of *Jatropha curcas* L. *J. Ethnopharmacol.* **2021**, *268*, 113673, doi:https://doi.org/10.1016/j.jep.2020.113673.

[64] Ricciotti, E.; FitzGerald, G.A. Prostaglandins and Inflammation. *Arterioscler. Thromb. Vasc. Biol.* **2011**, *31*, 986–1000, doi:10.1161/atvbaha.110.207449 CO - ATVBFA.

[65] Remy, S.; Olivon, F.; Desrat, S.; Blanchard, F.; Eparvier, V.; Leyssen, P.; Neyts, J.; Roussi, F.; Touboul, D.; Litaudon, M. Structurally Diverse Diterpenoids from *Sandwithia guyanensis*. *J. Nat. Prod.* **2018**, *81*, 901–912, doi:10.1021/acs.jnatprod.7b01025.

Comp.	Name(s); (CAS RN)	Activity	Effect	Target
	15-*epi*-4Z-Jatrogrossidenta-dion. (143062-70-8).		*Staphylococcus aureus* (ATCC 25923).[66]	
		Cytotoxic	Antiproliferative activity against A549, HELA, MDAMB231, HEPG2 and HEPG2-DOX cancer cell lines.[67]	
			Antiproliferative activity against A549, HL-60, SMMC-7721, MCF-7 and SW480 cancer cell lines.[69]	
**156, 157**	2-Hydroxy-isojatrogrossidion (**156**). (106644-32-0). 2-*epi*-Hydroxy-isojatrogrossidion (**157**). (143120-26-7).	Antibacterial.	Antibacterial against *Bacillus subtillis* (ATCC 6051) and *Staphylococcus aureus* (ATCC 25923).[66]	
		Cytotoxic.	Antiproliferative activity against A549, HELA, MDAMB231, HEPG2 and HEPG2-DOX cancer cell lines.[67]	
			Antiproliferative activity against A549, HL-60, SMMC-7721, MCF-7 and SW480 cancer cell lines.[69]	
			Antiproliferative activity against L5178Y mouse lymphoma cell line.[68]	
**S74**	Isojatrogrossidion. (143022-03-1).	Cytotoxic.	Antiproliferative activity against A549, HL-60, SMMC-7721, MCF-7 and SW480 cancer cell lines.[69]	
**S75**	2-*epi*-Isojatrogrossidion. (143120-25-6).			
**S76**	(4*E*)-Jatrogrossidenta-dion. (143050-04-8).	Cytotoxic.	Antiproliferative activity against A549, HELA (human cervical carcinoma), MDAMB231, HEPG2	

[66] Aiyelaagbe, O. O.; Adesogan, K.; Ekundayo, O.; Gloer, J. B. Antibacterial Diterpenoids from *Jatropha Podagrica* Hook. *Phytochemistry* **2007**, *68* (19), 2420–2425; doi: 10.1016/j.phytochem.2007.05.021.
[67] Zhang, J.-S.; Zhang, Y.; Li, S.; Ahmed, A.; Tang, G.-H.; Yin, S. Cytotoxic Macrocyclic Diterpenoids from *Jatropha Multifida*. *Bioorg. Chem.* **2018**, *80*, 511–518; doi: 10.1016/j.bioorg.2018.06.025.
[68] Chianese, G.; Fattorusso, E.; Aiyelaagbe, O.O.; Luciano, P.; Schröder, H.C.; Müller, W.E.G.; Taglialatela-Scafati, O. Spirocurcasone, a Diterpenoid with a Novel Carbon Skeleton from *Jatropha curcas*. *Org. Lett.* **2011**, *13*, 316–319, doi:10.1021/ol102802u.

Comp.	Name(s); (CAS RN)	Activity	Effect	Target
			and HEPG2-DOX (human hepatocellular carcinoma, doxorubicin-resistant) cancer cell lines.[67]	
			Antiproliferative activity against L5178Y mouse lymphoma cell line.[68]	
**S77**	Japodagrol. 15-epi-(4*E*)-Jatrogrossidenta-dion. (143120-24-5).	Cytotoxic.	Antiproliferative activity against A549 (human non-small-cell lung carcinoma), MDAMB231 (human breast carcinoma) and HEPG2 (human hepatocellular carcinoma) cancer cell lines.[67]	
			Antiproliferative activity against L5178Y mouse lymphoma cell line.[68]	
**S78**	Jatrogrossidion. (219494-36-7).	Cytotoxic	Antiproliferative activity against A549 (human non-small-cell lung carcinoma), HL-60 (human myeloid leukemia), SMMC-7721 (hepatocellular carcinoma), MCF-7 (breast cancer) and SW480 (colon cancer) cancer cell lines.[69]	

[69] Liu, J. Q.; Yang, Y. F.; Xia, J. J.; Li, X. Y.; Li, Z. R.; Zhou, L.; Qiu, M. H. Cytotoxic Diterpenoids from *Jatropha curcas cv. Nigroviensrugosus* CY Yang Roots. *Phytochemistry* **2015**, *117*, 462–468; doi: 10.1016/j.phytochem.2015.07.002.

Section S11. Bioactive ingol (**S87**) derivatives.

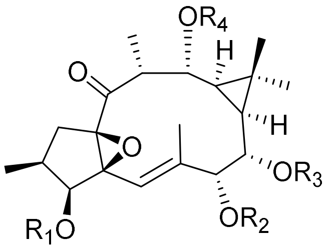

81, 101-103, 137-138,
141-142, 148, 165

Compound	R_1	R_2	R_3	R_4	CAS number	Common name
**81**	Ac	H	2-MeBu	Ac	1609953-48-1	Euphorantin N
**101**	Ac	Ang	Me	Ac	90027-10-4	-
**102**	H	Ang	Me	Ac	463297-88-3	-
**103**	Ac	H	Me	Ac	625830-44-6	-
**136**	Ac	H	Bz	Ac	124657-64-3	-
**137**	Tig	H	Bz	Ac	1151831-80-9	-
**140**	PhAc	Ac	Me	Ac	944799-48-8	-
**141**	Ac	H	Tig	Ac	58749-62-5	ELAC
**147**	Ac	Ac	Tig	Ac	1570052-92-4	AcELAC
**164**	Ac	Bz	Nic	Ac	462119-30-8	-

Ac = acetyl; Ang = angeloyl; Bz = benzoyl; PhAc = phenylacetyl. ;2-MeBu = 2-methylbutyryl; Tig = tigloyl.

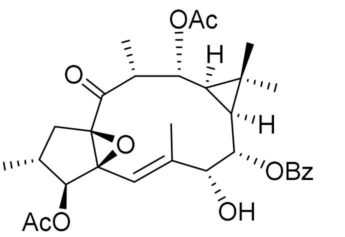

Euphorin D (**135**)
(CAS RN 2411265-90-0)

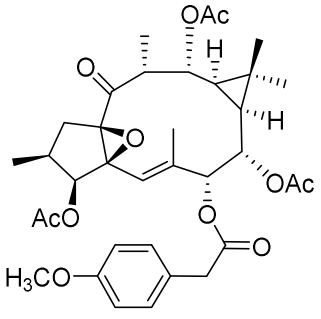

Euphorbia factor RL_4 /
EOF2 (**146**)
(CAS RN: 2230806-06-9)

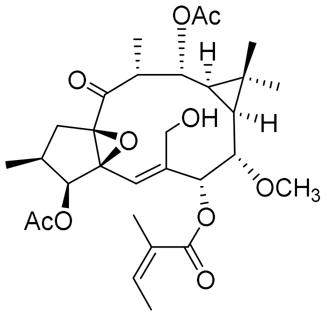

Euphorantin A (**163**)
(CAS RN: 1609953-25-4)

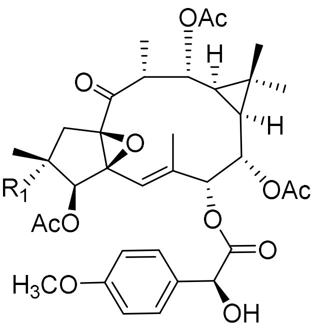

R_1=H, Euphorblin B (**165**)
(CAS RN: 2230805-88-4)
R_1=OH, Euphorblin D (**166**)
(CAS RN: 2230805-91-9)

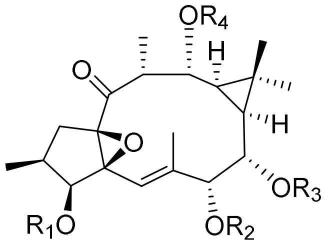

S79-S86

Compound	R_1	R_2	R_3	R_4	CAS RN	Common name
**S79**	2-MeBu	Ac	Ac	Ac	988298-74-2	
**S80**	Ac	2-MeBu	Ac	Ac	988298-75-3	
**S81**	Ac	Ac	2-MeBu	Ac	988298-76-4	
**S82**	Ac	Ac	Ac	2-MeBu	988298-77-5	
**S83**	2-MeBu	Ac	Me	Ac	988298-78-6	
**S84**	Ac	2-MeBu	Me	Ac	988298-79-7	
**S85**	Ac	Ac	Me	2-MeBu	988298-80-0	
**S86**	H	Ac	2-MeBu	Ac	1609953-45-8	Euphorantin M

Ac = acetyl; 2-MeBu = 2-methylbutyryl.

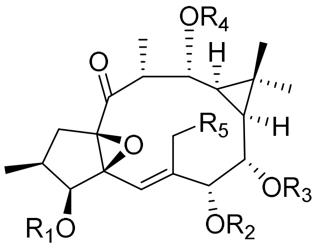

S(87-98)

Compound	R1	R2	R3	R4	CAS RN	Common name
**S87**	H	H	H	H	51847-86-0	Ingol
**S88**	Ac	Bz	Ac	Ac	1152414-02-2	-
**S89**	Ac	Tig	Ac	Ac	92910-93-5	-
**S90**	Ac	Bz	Me	Ac	2267305-62-2	-
**S91**	H	Ang	Me	Ac	463297-88-3	-
**S92**	H	Tig	Me	Ac	-	-
**S93**	H	Bz	Me	Ac	1609953-35-6	Euphorantin I
**S94**	H	Ang	Me	H	-	-
**S95**	Ac	H	Me	Ac	625830-44-6	-
**S96**	H	Ac	Bz	Ac	2416961-70-9	-
**S97**	Ac	Ac	Bz	Ac	462119-29-5	-
**S98**	Ac	Ang	Me	H	625830-43-5	-

Ac = acetyl; Ang = angeloyl; iBu = isobutyroyl; Bz = benzoyl; PhAc = phenylacetoyl; Nic = nicotinoyl; Tig = tigloyl.

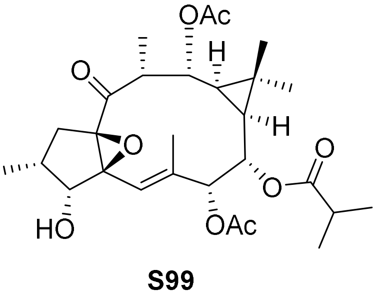

S99
(CAS RN 192825-65-3)

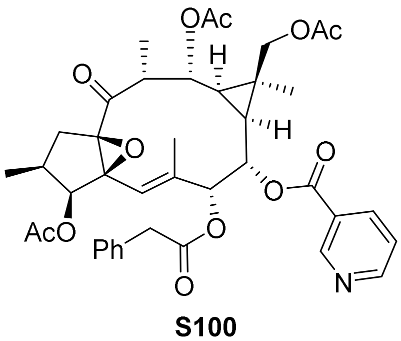

S100
(CAS RN 183480-86-6)

Table S11. Bioactive ingol (**S87**) derivatives: biological activities, observed effects and molecular targets (where available).

Number	Name(s); (CAS RN)	Activity	Effect	Target
**81**	Euphorantin N (1609953-48-1). 3,12-*O*-diacetyl-8-*O*-2-methylbutyrylingol	MDR reversal ability.	Inhibition of the efflux-pump activity.[18]	P-gp.
		Obesity and metabolic and cardiovascular disease	Inhibition of 11β-Hydroxysteroid Dehydrogenase Type 1 (11β-HDS1).[70]	11β-HDS1.
**101**	7-O-angeloyl-3,12-*O*-diacetyl-8-*O*-methylingol. (90027-10-4).	PGE_2 inhibition	PGE_2 inhibition.[79]	
		Cytotoxic	Cytotoxic activity against Colo 205, MT2, and CEM cell lines.[80]	
**102**	7-angeloyl-12-acetyl-8-methoxyingol. (463297-88-3).	Cytotoxic	-Antiproliferative activity against Colo 205, MT2, CEM cell lines.[80]	
		Prostaglandin E2 inhibitor.	PGE2 inhibition.[79]	
**103**	3,12-diacetyl-7-hydroxy-8-methoxyingol. (625830-44-6).	Cytotoxic against human tumor cell lines	-Antiproliferative activity against Colo 205, MT2, CEM cell lines.[80]	
**141**	3,12-*O*-diacetyl-8-*O*-tiglilingol. ELAC (58749-62-5)	Anti-osteoporosis towards BMM cells.	Inhibition of RANKL-induced osteoclastogenesis.[71]	
		HIV-1 reactivation.[72]	IκBα, JNK and ERK phosphorylation	PKC
			GFP induction	
		NPC proliferation.[73]		PKC β
		MDR reversal ability.	Inhibition of the efflux-pump activity.[18]	P-gp.
**136**	3,12-*O*-diacetyl-8-*O*-benzoylingol. (124657-64-3).			
		Anti-inflammatory	Inhibition the production of NO in LPC-induced murine	COX-2 protein.

[70] Qi, W.-Y.; Zhang, W.-Y.; Shen, Y.; Leng, Y.; Gao, K.; Yue, J.-M. Ingol-Type Diterpenes from *Euphorbia antiquorum* with Mouse 11β-Hydroxysteroid Dehydrogenase Type 1 Inhibition Activity. *J. Nat. Prod.* **2014**, *77*, 1452–1458, doi:10.1021/np5002237.
[71] Yin, Z.-Y.; Dai, Y.; Hua, P.; Sun, Z.-J.; Cheng, Y.-F.; Yuan, S.-H.; Chen, Z.-Y.; Gu, Q. Discovery of diverse diterpenoid scaffolds from *Euphorbia antiquorum* and their activity against RANKL-induced osteoclastogenesis. *Bioorg. Chem.* **2019**, *92*, 103292, doi:https://doi.org/10.1016/j.bioorg.2019.103292.
[72] Avila, L.; Perez, M.; Sanchez-Duffhues, G.; Hernández-Galán, R.; Muñoz, E.; Cabezas, F.; Quiñones, W.; Torres, F.; Echeverri, F. Effects of diterpenes from latex of *Euphorbia lactea* and *Euphorbia laurifolia* on human immunodeficiency virus type 1 reactivation. *Phytochemistry* **2010**, *71*, 243–248, doi:10.1016/j.phytochem.2009.10.005.
[73] Murillo-Carretero, M.; Geribaldi-Doldán, N.; Flores-Giubi, E.; García-Bernal, F.; Navarro-Quiroz, E.A.; Carrasco, M.; Macías-Sánchez, A.J.; Herrero-Foncubierta, P.; Delgado-Ariza, A.; Verástegui, C.; et al. ELAC (3,12-di-O-acetyl-8-O-tigloilingol), a plant-derived lathyrane diterpene, induces subventricular zone neural progenitor cell proliferation through PKCβ activation. *Br. J. Pharmacol.* **2017**, *174*, 2373–2392, doi:10.1111/bph.13846.

Number	Name(s); (CAS RN)	Activity	Effect	Target
			microglial BV-2 cells.[74]	
**135**	Euphorin D (2*R*)-8-*O*-benzoyl-3,12-*O*-diacetylingol. (2411265-90-0).	Anti-inflammatory.	-Inhibition the production of NO in LPC-induced murine microglial BV-2 cells.[74]	COX-2 protein.
**137**	12-*O*-acetyl-8-*O*-benzoyl-3-*O*-tigloylingol. (1151831-80-9).			
**140**	7,12-*O*-diacetyl-3-*O*-phenylacetyl-8-*O*-methylingol. (944799-46-6).	HIV-1 reactivation.[75]	- Cell-cycle arrest induction.<br>- HIV-1-LTR promotion.	PKC.
**146**	Euphorbia factor RL_4. Euphorblin R. EOF2. 3,8,12-*O*-triacetyl-7-(*p*-methoxy phenyl)acetylingol (2230806-06-9).	Induction of lysosomal biosynthesis.[76]		
		NPC differentiation.[77]	- Release of neuregulin 1.<br>- Neuroblast migration induction.	PKC θ
**147**	3,7,12-*O*-triacyl-8-*O*-tiglylingol. AcELAC (1570052-92-4).	NPC proliferation.[73]		PKC β
**163**	Euphorantin A. 7-*O*-Angeloyl-3,12-*O*-diacetyl-8-*O*-methylingol. (1609953-25-4).	Obesity and metabolic and cardiovascular disease	Inhibition of 11β-Hydroxysteroid Dehydrogenase Type 1 (11β-HDS1).[70]	11β-HDS1
**164**	3,12-diacetyl-7-benzoyl-8-nicotinylingol. (462119-30-8).			
**165**	Euphorblin B.	Induction of lysosomal biosynthesis.[76]		

[74] An, L.; Liang, Y.; Yang, X.; Wang, H.; Zhang, J.; Tuerhong, M.; Li, D.; Wang, C.; Lee, D.; Xu, J.; et al. NO inhibitory diterpenoids as potential anti-inflammatory agents from *Euphorbia antiquorum*. *Bioorg. Chem.* **2019**, *92*, 103237, doi:10.1016/j.bioorg.2019.103237.

[75] Daoubi, M.; Marquez, N.; Mazoir, N.; Benharref, A.; Hernández-Galán, R.; Muñoz, E.; Collado, I.G. Isolation of new phenylacetylingol derivatives that reactivate HIV-1 latency and a novel spirotriterpenoid from *Euphorbia officinarum* latex. *Bioorganic Med. Chem.* **2007**, *15*, 4577–4584, doi:10.1016/j.bmc.2007.04.009.

[76] Zhao, N.D.; Ding, X.; Song, Y.; Yang, D.Q.; Yu, H.L.; Adelakun, T.A.; Qian, W.D.; Zhang, Y.; Di, Y.T.; Gao, F.; et al. Identification of Ingol and Rhamnofolane Diterpenoids from *Euphorbia resinifera* and Their Abilities to Induce Lysosomal Biosynthesis. *J. Nat. Prod.* **2018**, *81*, 1209–1218, doi:10.1021/acs.jnatprod.7b00981.

[77] Domínguez-García, S.; Geribaldi-Doldán, N.; Gómez-Oliva, R.; Ruiz, F.A.; Carrascal, L.; Bolívar, J.; Verástegui, C.; Garcia-Alloza, M.; Macías-Sánchez, A.J.; Hernández-Galán, R.; et al. A novel PKC activating molecule promotes neuroblast differentiation and delivery of newborn neurons in brain injuries. *Cell Death Dis.* **2020**, *11*, doi:10.1038/s41419-020-2453-9.

Number	Name(s); (CAS RN)	Activity	Effect	Target
	(2230805-88-4).			
**166**	Euphorblin D. (2230805-91-9).			
**S79**	7,8,12-*O*-triacetyl-3-*O*-(2-methyl)butanoylingol (988298-74-2).	Cytotoxic.	Cytotoxic activity against human KB cells.[78]	
**S80**	3,8,12-*O*-triacetyl-7-*O*-(2-methyl)butanoylingol (928298-75-3).			
**S81**	3,7,12-*O*-triacetyl-8-*O*-(2-methyl)butanoylingol (929298-76-4).			
**S82**	3,7,8-*O*-triacetyl-12-*O*-(2-methyl)butanoylingol (928298-77-5).			
**S83**	7,12-*O*-diacetyl-3-*O*-(2-methyl)butanoyl-8-*O*-methylingol. (928298-78-6)).			
**S84**	3,12-*O*-diacetyl-7-*O*-(2-methyl)butanoyl-8-*O*-methylingol. (928298-79-7).			
**S85**	3,7-*O*-diacetyl-12-*O*-(2-methyl)butanoyl-8-*O*-methylingol. (928298-80-0).			
**S86**	Euphorantin M 7,12-*O*-diacetyl-8-*O*-(2-methyl)-butanoylingol. (1609953-45-8).	Obesity and metabolic and cardiovascular disease	Inhibition of 11β-Hydroxysteroid Dehydrogenase Type 1 (11β-HDS1).[70]	11β-HDS1
**S88**	3,8,12-*O*-triacetyl-7-*O*-benzoylingol (1152414-02-2).	MDR reversal ability.	Inhibition of the efflux-pump activity.[18]	P-gp.
**S89**	3,8,12-*O*-triacetyl-7-*O*-tigloylingol. (92910-93-5).			
**S90**	3,12-*O*-diacetyl-7-*O*-benzoyl- 8-*O*-methylingol. (2267305-62-2).	MDR reversal ability.	Inhibition of the efflux-pump activity.[18]	P-gp.

[78] Baloch, I.B.; Baloch, M.K.; Saqib, Q.N.U. Cytotoxic macrocyclic diterpenoid esters from *Euphorbia cornigera*. *Planta Med.* **2006**, *72*, 830–834, doi:10.1055/s-2006-946683.

Number	Name(s); (CAS RN)	Activity	Effect	Target
**S91**	12-*O*-acetyl-7-*O*-angeloyl-8-*O*-methylingol. (463297-88-3).	PGE_2 inhibition.	PGE_2 inhibition.[79]	
		Cytotoxic	Cytotoxic activity against Colo 205, MT2, and CEM cell lines.[80]	
		MDR reversal ability.	Inhibition of the efflux-pump activity.[18]	P-gp.
**S92**	12-*O*-acetyl-8-*O*-methyl7-*O*-tigloylingol.			
**S93**	Euphorantin I. 12-*O*-acetyl-7-*O*-benzoyl-8-*O*-methylingol. (1609953-35-6).			
**S94**	7-*O*-tiglyl-8-*O*-methylingol.			
**S95**	3,12-*O*-diacetyl-8-*O*-methylingol. (625830-44-6).	Cytotoxic	Antiproliferative activity against Colo 205, MT2, CEM cell lines.[80]	
**S96**	7-*O*-benzoyl-7,12-*O*-diacetyl-ingol. (2416961-70-9).	Anti-osteoporosis towards BMM cells	Inhibition of RANKL-induced osteoclastogenesis.[71]	
**S97**	7-O-benzoyl-3,7,12-O-triacetylingol. (462119-29-5).			
**S98**	3-*O*-acetyl-7-*O*-angeloyl-8-methoxyingol. (625830-43-5).	Cytotoxic	-Antiproliferative activity against Colo 205, MT2, CEM cell lines.[80]	
**S99**	7,12-di-*O*-acetyl-8-*O*-isobutyryl-2,3-di-*epi*-Ingol. (192825-65-3).[81]	Vascular activity	Contraction of rabbit basilar and carotid arteries.[82,83]	

[79] Ravikanth, V.; Niranjan Reddy, V.L.; Prabhakar Rao, T.; Diwan, P. V.; Ramakrishna, S.; Venkateswarlu, Y. Macrocyclic diterpenes from *Euphorbia nivulia*. *Phytochemistry* **2002**, *59*, 331–335, doi:10.1016/S0031-9422(01)00461-7.

[80] Ravikanth, V.; Lakshmi Niranjan Reddy, V.; Vijender Reddy, A.; Ravinder, K.; Prabhakar Rao, T.; Siva Ram, T.; Anand Kumar, K.; Prakesh Vamanarao, D.; Venkateswarlu, Y. Three new ingol diterpenes from *Euphorbia nivulia*: Evaluation of cytotoxic activity. *Chem. Pharm. Bull.* **2003**, *51*, 431–434, doi:10.1248/cpb.51.431.

[81] Wrongly indexed in CAS as 7,12-di-*O*-acetyl-8-butoxyl-2,3-di-*epi*-ingol (CAS number 207346-95-0) in reference 83; structure is correct in original paper (CAS RN 192825-65-3).

[82] Marco, J.A.; Sanz-Cervera, J.F.; Yuste, A. Ingenane and lathyrane diterpenes from the latex of Euphorbia canariensis. *Phytochemistry* **1997**, *45*, 563–570, doi:https://doi.org/10.1016/S0031-9422(97)00018-6.

[83] Miranda, FJ; Alabadi, JA; Orti, M.; Centeno, JM; Pión, M.; Yuste, A.; Sanz-Cervera, J.F.; Marco, J.A.; Alborch, E. Comparative Analysis of the Vascular Actions of Diterpenes Isolated from Euphorbia canariensis. *J. Pharm. Pharmacol.* **1998**, *50*, 237–241, doi:10.1111/j.2042-7158.1998.tb06182.x.

Number	Name(s); (CAS RN)	Activity	Effect	Target
**S100**	3,12,19-tri-*O*-acetyl-8-*O*-nicotinyl-7-*O*-phenylacetyl-19-hydroxyingol. (183480-86-6).	Cytotoxic against human tumor cell lines	Antiproliferative activity against lung carcinoma (A-549), breast carcinoma (MCF-7), colon adenocarcinoma (HT), kidney carcinoma (A-498), prostrate adenocarcinoma (PC-3) and pancreatic carcinoma (PACA-2).[84]	

[84] Fatope, M.O.; Zeng, L.; Ohayagha, J.E.; McLaughlin, J.L. New 19-acetoxyingol diterpenes from the latex of Euphorbia poisonii (Euphorbiaceae). *Bioorganic Med. Chem.* **1996**, *4*, 1679–1683, doi:10.1016/0968-0896(96)00157-5.
